# Irritability as a Transdiagnostic Construct Across Childhood and Adolescence: A Systematic Review and Meta-analysis

**DOI:** 10.1007/s10567-024-00512-4

**Published:** 2025-01-20

**Authors:** Miriam Chin, Davina A. Robson, Hannah Woodbridge, David J. Hawes

**Affiliations:** 1 School of Psychology, The University of Sydney, Sydney, NSW 2006 Australia; 2 School of Psychology, The University of Wollongong, Wollongong, NSW Australia; 3 School of Education, University of New South Wales, Sydney, NSW Australia

**Keywords:** Irritability, Emotion dysregulation, Transdiagnostic, Emotional reactivity, Externalizing problems, Internalizing problems

## Abstract

This meta-analytic review examined irritability across childhood and adolescence as it relates to symptoms of common mental health disorders in these periods. Of key interest was whether the relationship between irritability and symptom severity varies according to symptom domain. This was tested at the level of broad symptom dimensions (internalizing versus externalizing problems) as well as discrete diagnostic domains (e.g., anxiety, depression, oppositional defiant disorder, conduct disorder, attention deficit hyperactivity disorder; autism spectrum disorder). Following PRISMA guidelines, a systematic search of five databases was conducted to identify studies reporting on associations between irritability and mental health symptoms in samples of children aged 2–18 years. Meta-analytic tests based on random effects models examined concurrent and longitudinal associations between irritability and symptom severity. Meta-regression tested potential moderators including symptom domain, child age, sex, informant type, and study quality. 119 studies met inclusion criteria with a total of 122,456 participants. A significant and positive association was found between irritability and severity of concurrent overall psychopathology in the order of a moderate effect size, while small to moderate effect sizes characterized the association between irritability and later mental health outcomes in prospective data. Further variation in this association was seen across specific diagnostic domains and methodological moderators. Findings support the conceptualization of irritability as a transdiagnostic construct reflecting emotion dysregulation across diverse forms of psychopathology in childhood and adolescence. Further research into the risk mechanisms underlying irritability is needed, in addition to translational approaches to early intervention.

**Supplementary Information:**

The online version contains material available at 10.1007/s10567-024-00512-4.

## Introduction

Irritability has long featured in the clinical descriptions of many psychological disorders, however, it is only in recent years that the construct has been subject to extensive scientific investigation in its own right. Recent interest in the construct has arisen in part from assertions that it represents a key transdiagnostic construct implicated in diverse forms of psychopathology, with developmental origins early in life (Beauchaine & Tackett, 2020; Klein et al., 2021). Accordingly, disorders of childhood and adolescence have been a key focus of emerging research into irritability, which has spanned topics from measurement and diagnostic models, through to risk mechanisms and intervention targets (e.g., Leibenluft et al., 2024; Zachary & Jones, 2019). Moreover, there is growing recognition that irritability is one of the most common reasons for referral to mental health services in childhood (Evans et al., 2023). Irritability nonetheless remains a somewhat elusive construct, and researchers have just begun to synthesize the expansive evidence base that now exists, through systematic approaches such as meta-analysis (Brotman et al., 2017; Finlay-Jones et al., 2024; Vidal-Ribas et al., 2016).

Current definitions of irritability refer primarily to low frustration tolerance (Toohey & DiGiuseppe, 2017) or a low threshold for experiencing anger in response to slight provocation, incongruent with developmental age (Brotman et al., 2017). In diagnostic models, irritability has often been operationalized as a categorical symptom and conceptualized largely as a state-dependent mood lasting days to weeks, occurring in the absence of an obvious trigger. In literature on individual differences concerning temperament and personality, irritability has been conceptualized as the extreme expression of a dimensionally distributed trait that is heritable and relatively stable from early childhood to adulthood (Beauchaine & Tackett, 2020; Leibenluft & Stoddard, 2013).

Researchers have employed a wide range of measurement strategies to capture various aspects of irritability in child and adolescent samples, such as indexing mood versus behavior or persistent irritability versus temper outbursts. Common approaches have included the selection of specific symptom items from diagnostic interviews (e.g., Kiddie Schedule for Affective Disorders and Schizophrenia; Kaufman et al., 2000), subscales from temperament questionnaires (e.g., Child Behavior Questionnaire; Rothbart et al., 2001), along with instruments specifically designed to index irritability (e.g., Affective Reactivity Index; Stringaris et al., 2012). Research suggests that the prevalence and severity of irritability may significantly differ depending on whether the measure is self-report, parent-report, or teacher report (De Los Reyes et al., 2015; Evans et al., 2023). Moreover, from a developmental perspective, the age at which irritability is indexed can have important implications for conceptualization and measurement. There is support for the measurement of irritability as a distinct temperament dimension from infancy (Rothbart, 2011), however, studies of infants and toddlers have often operationalized irritability using non-specific indices (e.g., excessive crying) or broader dimensions such as negative emotionality that encompass other forms of distress (Finlay-Jones et al., 2024). As a specific mental health construct, irritability can present at clinically significant levels in children as young as 2 years (Camacho et al., 2019). Measures of irritability have often been used to operationalize the clinical expression of emotion dysregulation, which itself encompasses processes including emotional expressions and experiences that are inappropriate to context or excessive based on social norms; shifts in emotion that are rapid or poorly controlled; and the atypical allocation of attention to emotional stimuli (Evans et al., 2021a; Vogel et al., 2019; Shaw et al., 2014).

Evidence regarding irritability has important implications for the diagnosis and classification of child and adolescent psychopathology. The *Diagnostic and Statistical Manual of Mental Disorders* (DSM-5) (American Psychiatric Association; APA, 2013) and *The International Classification of Disease* (ICD-11) (World Health Organization, 2022) both include irritability as a symptom in the criteria for disorders including oppositional defiant disorder (ODD), major depressive disorder in children and adolescents, and generalized anxiety disorder. ODD, for example, includes a distinct symptom dimension reflecting chronic irritability, which is thought to account in part for findings that ODD in childhood is a common precursor to many other forms of psychopathology across adolescence and later life (e.g., depression, suicidality, substance use, psychosis) (Burke et al., 2021; Hawes et al., 2023). Recent revisions to the major diagnostic systems also reflect ongoing debate regarding the classification of chronic irritability (Evans et al., 2021a; Fristad, 2021; Runions et al., 2016). For example, DSM-5 included a new childhood mood disorder, Disruptive Mood Dysregulation Disorder (DMDD), whereas ICD-11 introduced chronic irritability as a specifier within ODD (See also: Copeland et al., 2013; Lochman et al., 2015).

Interest in irritability as a transdiagnostic construct has grown significantly in recent years, driving considerable research into associations between irritability and various symptom domains. In addition to testing these associations through methods such as factor analysis and network analysis (e.g., Burke et al., 2014; Tseng et al., 2023), meta-analysis has been used in two studies to date. Vidal-Ribas et al. (2016) analyzed data from 12 longitudinal studies of chronic non-episodic irritability as a predictor of future mental health disorders. Irritability was reported to be significantly associated with depression and anxiety but not conduct problems such as ODD and conduct disorder (CD), and research priorities were identified, including the need for studies using high-quality measures of irritability. Research in the area has since grown rapidly, and a number of such measures have been widely adopted, including the Affective Reactivity Index, which is recommended in DSM-5 as a cross cutting symptom measure for irritability in 6–17-year-olds (APA, 2013).

A more recent meta-analysis of 98 longitudinal studies focused specifically on indices of irritability prior to 5 years of age, as predictors of later disorders across childhood and adolescence. Results indicated a small but significant association between infant (0–12 months) and preschool/toddler (13–60 months) irritability and later internalizing and externalizing disorders (Finlay-Jones et al., 2024). The meta-analytic findings of Finlay-Jones et al. (2024) support early irritability during ages 0–5 years as a transdiagnostic neurodevelopmental vulnerability to later psychopathology. At the same time, there exists an extensive and largely separate body of research in which irritability and broader symptoms of psychopathology have been indexed across childhood and adolescence. It is this evidence base that is the focus of our review.

## Aims of the Current Review

Our major aim of the current review was to examine associations between irritability and psychopathology across childhood and adolescence (ages 2–18 years). Data on irritability across childhood and adolescence have important implications for understanding irritability as a feature of symptom clusters that covary together during these periods. By conducting the first meta-analytic test of these associations we aim to further inform diagnostic and transdiagnostic models of child and adolescent mental health. Given that Finlay-Jones et al. (2024) focused specifically on irritability in infancy and early childhood, the authors had good justification for including studies in which irritability was operationalized using indices such as fussy temperament, negative emotionality, and excessive crying. Conversely, many studies of irritability as a distinct construct could not be included by Finlay-Jones et al. (2024), because some of the most established and widely used measures of irritability, such as the Affective Reactivity Index, are designed for older children. These studies form the bulk of the evidence base examined in our review, which was limited to data from high quality measures of the specific construct of irritability.

A further aim was to examine how the relationship between irritability and mental health varies as a function of symptom domain. In addition to testing the relationship between irritability and overall symptoms of psychopathology, it was tested also according to broad symptom dimensions (internalizing versus externalizing problems), and a number of more discrete diagnostic domains (anxiety; depression; obsessive compulsive disorder; ODD; conduct disorder; attention deficit hyperactivity disorder [ADHD]; substance use disorder; suicidality/self-harm; tic disorders and autism spectrum disorder [ASD]). These associations were examined both concurrently and prospectively, based on follow-up testing across distinct developmental periods. Finally, in order to help resolve mixed findings from previous research, a number of moderators were tested, consisting of child age, sex, informant type, and study quality.

## Method

### Search Strategy

This meta-analysis was conducted in accordance with the Preferred Reporting Items for Systematic Reviews and Meta-analyses (PRISMA; Page et al., 2021; see Supplementary Table S1), and registered with Prospero (CRD42023404623). A search of five electronic databases was conducted in June 2024. The databases included were PubMed/MEDLINE, Web of Science, PsycINFO, PsycARTICLES, and Scopus. The search terms used were irritability [OR temperament OR negative affectivity] AND child [OR preschool OR early years OR adolescence] AND psychological [OR mental health OR psychopathology OR psychological disorder OR depression OR anxiety OR oppositional defiant disorder OR conduct disorder OR ADHD OR suicide OR autism OR internalizing OR externalizing OR substance OR abuse OR obsessive compulsive disorder OR eating disorder OR social functioning OR emotional functioning]. No date limits were set in the search or restrictions regarding language or the type of publication. A manual search was conducted on the reference list of studies that met inclusion criteria to identify relevant studies that were not included in the databases. In cases where studies did not report sufficient information to convert a score into a zero-order correlation, additional data were requested by email. A total of 58 authors were contacted for this purpose, with data provided by 20 (a response rate of 34%).

### Eligibility Criteria

Studies that reported on measures of irritability in children and adolescents between the ages of 2 and 18 years, in relation to mental health outcomes, were eligible for inclusion. Measures of irritability were assessed to determine that they operationalized irritability as *low frustration tolerance* (Toohey & DiGiuseppe, 2017). Based on this approach, some temperament subscales reflecting this definition of irritability were included. Self-report, parent-report and teacher-report measures of irritability were included, as were observational measures. No restrictions were placed on sample type (e.g., clinical, community-based). Review papers, conference abstracts, books, and case studies, were excluded, as were studies not available in English.

### Study Selection

**Fig. 1:**
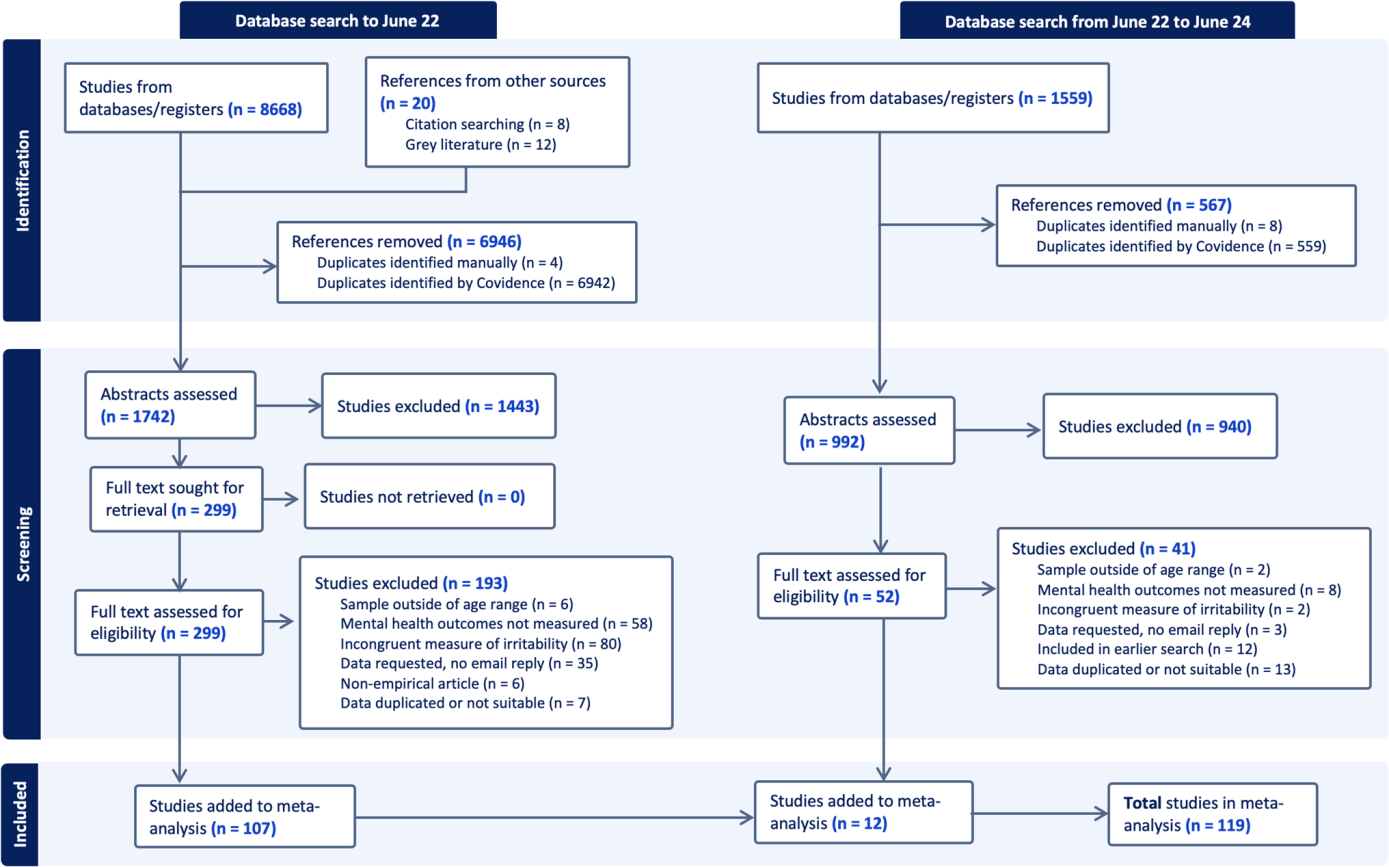
Flow diagram of study selection procedure

Two researchers independently screened the titles and abstracts of retrieved studies against the inclusion criteria. Full text manuscripts were then independently assessed by two reviewers and any discrepancies were resolved through discussion. Inter-rater reliability (Cohen’s kappa = 0.84) was calculated for the first 100 studies screened, indicating good agreement. Figure 1 summarizes the screening procedure.

### Data Extraction

Data extraction was performed by two researchers independently and then cross checked to resolve conflicts via discussion with the research team. The information extracted included study design, total sample size, country where the study was conducted, sex of participants (% male), mean age and standard deviation (or midpoint of age range), measure of irritability, informant type, and mental health outcomes. The effect size metric extracted was the zero-order correlation coefficient (r). When this was not reported, other statistics (e.g., odds ratios, means and standard deviations) were converted to correlation coefficient (r) prior to analysis. This conversion was performed using the formulas provided in the Comprehensive Meta-Analysis 3.0 statistical software program (Borenstein et al., 2014).

Data were extracted for 24 mental health outcomes that had been correlated with irritability in childhood and adolescences. These outcomes were grouped into three broad categories: internalizing, externalizing and neurodevelopmental. Internalizing outcomes included: anxiety disorders (subcomponents: generalized anxiety, social anxiety, separation anxiety, obsessive–compulsive disorder, phobias), depressive disorders, affect dysregulation, suicidality and non-suicidal self-harm. Externalizing outcomes included: disruptive behavior disorders (subcomponents: oppositional defiant disorder, conduct disorder), attention-deficit/hyperactivity disorder, peer problems, antisocial behavior, substance use disorder, and disruptive behavior symptoms (subcomponents: argumentative/defiant, hurtfulness/vindictiveness, aggression, delinquency/rule breaking, callous-unemotional traits/limited prosocial emotions). Neurodevelopmental outcomes included: ASD and tic disorders.

#### Quality Assessment

The methodological quality of included studies was rated independently by two researchers using the AXIS tool (Downes et al., 2016). This 20-item scale indexes the quality of non-experimental studies across a range of domains including sample size justification, sample representativeness, use of validated measures, overview of analytic methods, information regarding non-response bias, and reporting of conflicts of interest and funding. Studies were assigned a score ranging from 0 to 20, with higher scores reflecting higher quality. Conflicts between the two independent raters were resolved in consultation with members of the research team. Details of the quality ratings are available in supplementary Table S2.

#### Meta-Analytic Strategy

Meta-analytic tests were conducted based on a random effects model. The inverse-variance weighted random effects model gives more weight to the mean of the effect sizes calculated from the more precise studies, based on the inverse of its variance (Borenstein et al., 2011). This assumes that the true effect from each study varies due to more than expected sample population differences and is best used in meta-analyses with heterogeneous studies.

Publication bias was assessed using the asymmetry depicted in a funnel plot graphic. The funnel plot maps the sample size against the effect size, and publication bias can be seen in asymmetry around the true effect size (Egger et al., 1997). Additionally, Egger’s regression asymmetry test was used to detect potential publication bias. This method uses a linear regression to assess asymmetry in the funnel plot (Egger et al., 1997). A significant value for Egger's regression asymmetry test (two tailed) indicates meaningful publication bias. Publication bias can be due to a number of reasons, for example, reporting biases and editorial decisions favoring larger statistically significant studies (Egger et al., 1997; Rothstein, 2008).

Heterogeneity, whereby studies differ from each other beyond expected random error, was measured using the *I*^*2*^ statistic. If studies are high in heterogeneity, it is reflected in a score of 75 or more; moderate heterogeneity in a score of around 50; low heterogeneity 25 (Higgins & Thompson, 2002). Thus, *I*^*2*^ above 50 and a significant *Q* statistic (hypothesis test of expected variation between studies) indicates the need to explain this effect by testing moderators (Gonzalez-Mulé & Aguinis, 2018). Moderation analysis was conducted using random-effects meta-regression, when *k* was greater than 10 and heterogeneity was evident; Borenstein et al., 2011).

Six models were planned, one incorporating concurrent data, and five longitudinal models, each based on separate datasets. The models were based on the developmental time interval between assessment of irritability and outcome measures. Model 2 included studies in which irritability was measured in preschool age (2–5 years) and outcomes in middle childhood (5–13 years); model 3 included middle childhood to later childhood/adolescence (8–18 years); model 4 included middle childhood to adulthood (18 +); model 5 included early adolescence (13–18 years) to late adolescence; model six included adolescence to adulthood. Theorized moderators included child age (mean age), sex (percentage of boys in the sample), informant type, (parent, teacher, or self-report) and study quality (AXIS tool score).

## Results

### Characteristics of Included Studies

Overall, data from 119 studies were analyzed, with 541 effect sizes. There were 77 studies reporting concurrent associations between irritability and mental health symptoms, with 417 effect sizes (*n* = 66,326). The grand mean age for concurrent associations was 10.6 (± 3.0) years. There were 42 studies reporting longitudinal associations, with 124 effect sizes (*n* = 56,130). Longitudinal model 2 (preschool to childhood; *n* = 13,964), included 15 studies representing 65 effect sizes. The grand mean age was 3.9 (± 1.2) years at baseline and 9.2 (± 3.1) years at follow-up. Longitudinal model 3 (childhood to later childhood/adolescence; *n* = 36,201) included 20 studies representing 39 effect sizes. The grand mean age was 8.1 (± 2.6) years at baseline and 12.4 (± 2.7) years at follow-up. Longitudinal model 4 (childhood to adulthood) included only one study, and therefore could not be conducted (the data from this study were included in model 6). Longitudinal model 5 (adolescence to adolescence; *n* = 3851) included 4 studies representing 11 effect sizes. The grand mean age was 14.5 (± 0.5) years at baseline and 16.5 (± 1.3) years at follow-up. Longitudinal model 6 (adolescence to adulthood; *n* = 1840) included 4 studies representing 9 effect sizes. The grand mean age was 14.4 (± 2.8) years at baseline and 22.1 (± 4.0) years at follow-up.

There was a total of 122,456 participants, including 57,593 females (47.0%) and 64,872 males (53.0%), with 4674 sex unknown (3.8%). The samples were from North and South America (*n* = 74), Europe (*n* = 28), Australia (*n* = 7), Asia (*n* = 7), and multicontinental (*n* = 3). There were 36 measures of irritability in the form of parent, self, and teacher reports. The mean score for study quality was 16.8 (± 1.5; range = 12–20), suggesting high reporting standards (Table S2). Characteristics of the included studies are displayed in Table 1.

**Table 1:** Characteristics of included studies

Study	*N*	Study design	Country	Child sex (% boys)	Age range or M (SD)			Measure of irritability	Mental health variables	Study quality	Model
					Baseline	Time 2	Time3				
Aebi et al. (2016)	158	Cross-sectional	Switzerland	Boys	16.89 (1.13)	–	–	Self: MINI-KID	Affective disorders Anxiety disorders Conduct disorder ADHD Suicidality	18	1
Aebi et al. (2013)	1031	Cross-sectional	Switzerland	Both (50%)	13.85 (1.63)	29.6 (1.63)	–	Self: CBCL-YSR Parent: CBCL	Anxious/depressed Attention problems Delinquent behavior Depression Any crime	18	1
Ali et al. (2022)	T1:409 T2: 394 T3: 365	Longitudinal	England	Both (49%)	3.43 (0.30)	5.93	8.59	Observation: LAB-TAB Irritability	Internalizing Externalizing Depression ODD ADHD Conduct disorder	16	2
Althoff et al. (2014)	S1: 2029 S2: 2076 S3: 399 Total: 4504	Cross-sectional	America Dutch	S1: Both (53%) S2: Both (49%) S3: Both (53%)	S1: 6–18 S2: 4–16 S3: 10.88 (3.06)	–	–	Parent: CBCL	ODD Conduct disorder Anxiety ADHD	15	1
Ambrosini et al. (2013)	500	Cross-sectional	America	Both (73%)	6–18	–	–	Self: K-SADS-P	Major depressive disorder ODD MDDD ADHD (combined) ADHD (inattentive)	15	1
Arana et al. (2021)	S1: 220 S2: 252 Total: 472	Cross-sectional	Netherlands	S1: Both (55%) S2: Both (50%)	S1: 11.54 (0.49) S2: 10.85 (0.57)	–	–	Parent: HiPIC – Neg. affectivity subscale	Internalizing Externalizing Attention problems Anxiety	17	1
Barclay et al. (2022)	T1: 231 T3: 192	Longitudinal	America	Both (68%)	7.4 (1.1)	9.72 (1.32)	12.09 (1.36)	Teacher: DBD Parent: DBD	Internalizing Externalizing	16	3
Barker & Salekin (2012)	5923	Longitudinal	America	Both (51%)	8	10	–	Parent: DAWBA	Internalizing CU traits Conduct problems Emotional difficulties Callous attitude Peer victimization	15	3
Baweja et al. (2021)	226	Cross-sectional	America	Both (73%)	7.6 (1.96)	–	–	Parent: DBDRS	Inattention Hyperactive-Impulsivity Inattentive-overactive-impulsivity Oppositional-defiant Emotional lability	17	1
Bell et al. (2023)	491	Cross-sectional	Australia	Boys	12–17			Self: BiTe	Conduct Problems	18	1
Benarous et al. (2020a)	30	Cross-sectional	Canada	Both (87%)	6–16	–	–	Parent: K-SADS-PL	Externalizing Internalizing Depression	15	1
Benarous et al. (2020b)	163	Cross-sectional	Canada	Both (61%)	7–17	–	–	Parent: K-SADS-PL	Anxiety disorders Substance use disorder	17	1
Bielas et al. (2016)	130	Cross-sectional	Switzerland	Boys	13–18	–	–	Self: CIS	Adverse child experience ADHD DBD SUD Suicidality Depressive disorders Anxiety disorders PTSD	16	1
Bolhuis et al. (2017)	T1: 6209 T2: 4724	Longitudinal	Netherlands	Both (50%)	6	10	–	Parent: CBCL	Physical aggression Oppositional behavior Disobedient behavior Rule-breaking behavior Callous traits	17	3
Brandes et al. (2019)	695	Cross-sectional	America	Both (48%)	7–13	–	–	Parent: CBCL	Internalizing symptoms Externalizing symptoms	16	1
Burke (2012)	T1:177 T2: 162	Longitudinal	America	Boys	7–12	17	–	Parent: DISC Parent: CSI-IV Parent: DAWBA	Depression Anxiety Conduct disorder ADHD symptoms	15	3
Burke et al., (2014)	S1: 1517 S2: 2451 S3: 7420 Total: 11,388	Cross-sectional Cross-sectional Cross-sectional	America America UK	Boys Girls Both (50%)	7 5–8 10–11	–	–	Parent: DISC Parent: CSI-IV Parent: DAWBA	Oppositional behavior	15	1
Busch et al. (2023)	194	Cross-sectional	Germany	Both (60%)	12.52 (2.69)	–	–	Self: BiTe	Anxiety Depression	17	1
Caprara et al. (2017)	109	Cross-sectional	Italy	Both (74%)	11–18	–	–	Parent: CIS	Aggression Emotional instability Hostile rumination Moral disengagement Agreeableness	17	1
Cardinale et al. (2021)	489	Cross-sectional	America	Both (55%)	12.03 (2.41)	–	–	Parent: ARI	ADHD Inattentive ADHD (Hyp/Imp)	17	1
Cardinale et al. (2019)	331	Cross-sectional	America	Both (46%)	13.57 (2.69)	–	–	Parent: ARI Self: ARI	ADHD Anxiety DMDD	17	1
Carter-Leno et al. (2021)	52	Cross-sectional	England	Both (60%)	13–17	–	–	Parent: ARI	Autism	17	1
Chad-Friedman et al. (2023)	605	Longitudinal	America	Both (54%)	3.52	9.23	15.25 (.40)	Parent: CBCL	Depression Anxiety Defiance ADHD	17	2
Chad-Friedman et al. (2022)	605	Longitudinal	America	Both (48%)	4.17	6.07	–	Parent: Daily diary of mood & affect	Depressive symptoms Anxiety symptoms ADHD symptoms ODD symptoms Global impairment	20	2
Chen et al. (2021)	131	Cross-sectional	America	Both (40%)	13 (2.79)	–	–	Self: ARI	Anxiety symptoms Bullying perpetration Peer victimization	17	1
Copeland et al. (2015)	1420	Cross-sectional	America	Both (50%)	9–16	–	–	Parent & Self: CAPA	Depression Anxiety Any impairment	15	1
Courbet et al. (2021)	170	Cross-sectional	France	Both (76%)	6–11	–	–	Parent: ARI	ADHD	19	1
Craig et al. (2021)	179	Longitudinal	Canada	Both (54%)	15.34 (1.53)	18	–	Self: OCHS	Attachment anxiety Attachment avoidance ODD symptoms Affect dysregulation	19	6
DeGroot et al. (2024)	2548	Cross-sectional	America	Both (69%)	12.21 (3.3)	–	–	Parent: CBCL Teacher: CASI	Depressive disorder Anxiety disorder ADHD (combined) ADHD (inattentive) ODD/CD Suicidality	17	1
Déry et al. (2017)	276	Longitudinal	Canada	Both (60%)	8.5 (0.9)	9–12	–	Parent: DISC	Depression Anxiety ADHD ODD (vindictive) ODD (defiant)	18	3
Doerfler et al. (2020)	310	Cross-sectional	America	Both (35%)	10.65 (3.4)	–	–	Parent: K-SADS-P	Depression Anxious/depressed Internalizing symptoms Aggressive behavior Externalizing symptoms Global impairment Total aggression	17	1
Dougherty et al. (2015)	446	Longitudinal	America	Both (55%)	3.51 (.26)	9.25 (.42)	–	Parent: PAPA	Depressive disorder Anxiety Disorder ADHD DBD	18	2
Dougherty et al. (2016)	473	Longitudinal	America	Both (54%)	6.10 (0.43)	9.21 (0.38)	–	Parent: PAPA	Depressive disorder Anxiety Disorder ADHD DBD	18	3
Dougherty et al. (2013)	462	Longitudinal	America	Both (54%)	3.6 (0.3)	6.1 (0.4)	–	Parent: PAPA	Global impairment Depressive disorder Anxiety disorder ADHD ODD	18	2
Drabick & Gadow (2012)	S1: 546 S2: 614 Total: 1160	Cross-sectional	America	S1: Both (73%) S2: Both (67%)	S1:6–11 S2: 12–18	–	–	Parent: CASI-4R, ASI-4R Teacher: CASI-4R, ASI-4R	ODD ADHD (Inattentive) ADHD (Hyp/Imp): Conduct disorder GAD Social anxiety disorder Major depressive disorder Manic symptoms	17	1
Dugre & Potvin (2020)	4898	Cross-sectional	Canada	Both (52%)	5	–	–	Parent: CBCL	Anxiety Hyperactivity Physical aggression	17	1
Elvin et al. (2021)	93	Cross-sectional	Australia	Both (56%)	9.96 (0.36)	–	–	Self: ARI	Self-regulation positive Self-regulation negative Behavioural control Prosocial behavior Peer problems Anxiety Depression Conduct Problems	17	1
Elvin et al., (2023)	84	Cross-sectional	Australia	Both (45%)	9.31 (2.44)	–	–	Parent: CBCL	Anxiety	16	1
Evans et al., (2021a, 2021b)	237	Cross-sectional	America	Both (64%)	9.14 (3.51)	–	–	Parent/youth (6–18): ARI Parent (3–5): ARI	Anxiety Depression Inattention Hyperactivity Suicidality Aggression	16	1
Evans et al. (2020a)	238	Cross-sectional	America	Both (52%)	8.9	–	–	Self: ARI	Anxiety Depressive symptoms Reactive aggression Oppositional behavior	17	1
Evans et al. (2020b)	1030	Cross-sectional	America	Both (58%)	10.2 (2.4)	–	–	Parent: CBCL Youth: CBCL-YSR	Internalizing Problems Externalizing Problems Anxious/Depressed Withdrawn/Depressed Somatic Complaints Social Problems Thought Problems Attention Problems Rule-Breaking DSM Affective Problems DSM Anxiety Problems DSM Somatic Problems DSM ADHD Problems DSM Conduct Problems	18	1
Evans et al. (2020c)	346	Longitudinal	America	Both (49%)	5–8	8–11	–	Teacher: DBDC Youth DBDC	Defiance Hyperactivity Inattention Depression	18	3
Evans et al. (2022)	206	Cross-sectional	America	Both (52%)	10.73 (2.40)	–	–	Caregiver: CBCL Youth: CBCL-YSR	Emotional Lability Anger coping Anger Dysregulation Internalizing Externalizing Attention	17	1
Evans et al. (2016)	706	Cross-sectional	America	Both (51%)	5–12	–	–	Teacher: DBD	Defiant Hyperactive-Impulsive Inattentive Proactive Aggression Reactive aggression Relational aggression Physical aggression Depression	16	1
Eyre et al. (2019)	4874	Longitudinal	England	Both (57%)	7–9	10–16	–	Parent: DAWBA	Neurodevelopmental difficulties Depression	18	3
Eyre et al. (2017)	696	Cross-sectional	UK	Both (84%)	10.9 (2.99)	–	–	Parent: CAPA	Anxiety Depression	17	1
Ezpeleta et al. (2012)	622	Cross-sectional	Spain	Both (51%)	3.0 (0.18)	–	–	Parent: DICA-PPYC	Disruptive ADHD Mood disorders Minor depression Anxiety disorders SAD Specific phobia Social phobia	17	1
Ezpeleta et al. (2019)	614	Cross-sectional	Spain	Both (50.2%)	3.8(0.33)	7	–	Parent: SDQ	Affective problems Anxiety problems ODD problems	19	1
Ezpeleta et al. (2020a)	T1: 471 T2: 454	Longitudinal	Spain	T1: Both (49%) T2: Both (49%)	7.7 (0.36)	11.6 (0.34)	–	Teacher: ARI	Anger SDQ-Conduct SDQ-Emotional SDQ-Hyperactivity SDQ-Peer SDQ-Prosocial SDQ-Externalizing (Conduct + Hyper) SDQ-Internalizing (Emotional + Peer) CAS-Verbal Aggression CAS-Aggression against objects/animals CAS-Physical aggression CAS-Use of weapons CAS-Aggression to peers CAS Total CBCL-Withdrawn/Depressed CBCL-Anxious/Depressed CBCL-Attention problems CBCL-Aggressive behavior CBCL-Rule-Breaking ERC-Lability-Negativity ERC-Emotion regulation YSR-Withdrawn/Depressed YSR-Anxious/depressed YSR-Attention problems YSR-Aggressive behavior YSR-Rule-Breaking AQ-Tolerance to frustration AQ-External expression AQ-Anger control	19	3
Ezpeleta et al. (2020b)	Cl1: 332 Cl2: 55 CI3: 108 Total: 495	Longitudinal	Spain	Cl1: Both (52%) Cl2: Both (53%) Cl3: Both (47%)	3	11–12	–	Parent: SDQ	ASR-Anxious/depressed ASR-withdrawn ASR-somatic complaints ASR-thought problems ASR-Attention problems ASR-Aggressive behavior ASR-rule-breaking ASR-Intrusive ASR-internalizing ASR-externalizing SDQ-emotion SDQ-conduct SDQ- hyperactivity SDQ-peer SDQ-prosocial Functional impairment	18	2
Ezpeleta et al. (2022)	563	Cross-sectional	Spain	Both (51%)	6–13	–	–	Parent: SDQ	Trajectory-Defiant Trajectory-OCP	18	1
Farchione et al. (2007)	Target: 300 Control: 169	Cross-sectional	America	Target: Both (52%) Control: Both (44%)	Target: 12.1 (3.6) Control: 11.6 (3.5)	–	–	Parent: CHI Self: CHI	Aggression Hostility	16	1
Fernandez de la Cruz et al. (2015)	579	Cross-sectional	America	Both (80%)	8.5 (0.8)	–	–	Parent SNAP	Withdrawn Somatic complaint Anxious/depression Social problems Thought problems Attention problems Aggressive behavior Internalizing scale Externalizing scale	15	1
Filippi et al. (2020)	291	Cross-sectional	America	Both (46%)	2–12	–	–	Parent: ARI (12y) Self: ARI (12y)	Social Anxiety	15	1
Gadow & Drabick (2012)	1127	Cross-sectional	America	Both (70%)	12.1 (3.4)	–	–	Parent: CASI-4R Teacher: CASI-4R	ODD ADHD (inattentive) ADHD (hyperactive) CD GAD OCD Social anxiety Depressive symptom Manic	16	1
Galano et al. (2023)	T1: 120 T2: 71 T3: 68	Longitudinal	America	T1: Both (50%) T2: Both (54%) T3: Both (57%)	4.94 (0.85)	+ 6–8 months	12.46 (1.77)	Parent: CBCL	Internalizing Externalizing Emotion Regulation Prosocial Behaviour	18	3
Grabell et al. (2020)	79	Cross-sectional	America	Both (52%)	3–7	–	–	Parent: MAP-DB	Externalizing	14	1
Guzick et al. (2021)	161	Cross-sectional	America	Both (51%)	7–17	–	–	Parent: CBCL	Depressive symptoms Anxiety OCD	16	1
Harima et al. (2022)	116	Cross-sectional	Japan	Boys	11.8 (2.6)	–	–	Self & Parent Clinical Interview	Internalizing Externalizing Anxiety/Depression	20	1
Hawes et al. (2020)	T1: 941 T2: 941 T3: 816	Longitudinal	America	T1: Both (43%) T2: Both (43%) T3: Both (41%)	16.6 (1.2)	24	30	Self: K-SADS	Depressive disorder Anxiety disorders Substance use disorder	18	6
Kahle et al. (2021)	T1:108 T2: 80	Longitudinal	America	T1: Both (67%) T2: Both (60%)	12–16	13–17	–	Parent: CPRS-3	Hyperactivity /Impulsivity Inattention	19	5
Kalvin et al. (2021)	81	Cross-sectional	America	Both (76%)	8–16	–	–	Parent: ARI	Autism	16	1
Kessel et al. (2021)	541	Longitudinal	America	Both (57%)	3.55 (0.43)	12.75 (0.50)	–	Parent: PAPA	Internalizing Externalizing	17	2
Kessel et al., (2017)	338	Cross-sectional	America	Both (56%)	3	–	–	Parent: PAPA	Depression Anxiety Disruptive Behavior Disorder	17	1
Kessel et al. (2016)	T1: 541 T2: 304	Longitudinal	America	Both (57%)	3.55 (0.43)	9.14 (0.32)	–	Parent: PAPA	Internalizing Externalizing	16	2
Khurana et al. (2023)	142	Cross-sectional	America	Both (58%)	10.51	–	–	Self: ARI	Depression Suicidality	19	1
Kishida et al. (2022)	1867	Cross-sectional	Japan	Both (53%)	10.53 (2.63)	–	–	Parent: ARI	Emotional symptoms Conduct problems Hyperactivity/Inattention Peer relationship problems Prosocial behavior	17	1
Kolko et al., (2007)	242	Cross-sectional	America	Both (80%)	9.1 (2.0)	–	–	Parent: SCAR-H Teacher: SCAR-S	Aggression	17	1
Kolko & Pardini (2010)	177	Cross-sectional	America	Both (81%)	6–11	–	–	Parent & Self: K-SADS	Global functioning Overall impairment ADHD Conduct Disorder CU traits ODD (hurtful) ODD (headstrong)	17	1
Krieger et al. (2013)	2514	Cross-sectional	Brazil	Both (54%)	6–12	–	–	DAWBA	Emotional disorders Anxiety disorder Major depression Conduct Disorder ADHD Peer problems Pro-sociality	18	1
Leadbeater & Ames (2017)	662	Longitudinal	Canada	Both (48%)	15.5(1.9)	26.8	–	Parent: BCFPI	Internalizing symptoms Conduct problems ADHD symptoms	19	6
Lee et al. (2023)	285	Longitudinal	Taiwan	Both (49%)	9.9 (0.6)	6 months later	9 months later	Parent: CBCL	Anxiety	19	3
Legenbauer et al. (2018)	91	Cross-sectional	Germany	Both (30%)	13.98 (1.31)	26.8(2.0)	–	Self: ARI	Affective dysregulation	17	1
Leibenluft et al. (2006)	T1: 776 T2: 776 T3: 717	Longitudinal	America	Both (50%)	13.8 (2.6)	16.2 (2.8)	–	Parent: DISC Self: DISC	ADHD Conduct Disorder ODD Depression GAD OCD Simple phobia Social phobia Mania	17	5
Leigh et al. (2020)	T1: 165 T2: 156	Cross-sectional	England	Both (57%)	12–14	–	–	Self: ARI	Depressive rumination Angry rumination	14	1
Lengua (2006)	T1: 214 T2: 204	Longitudinal	America	Both (45%)	9.48 (1.01)	+ 3 years	–	Parent: EATQ	Internalizing Externalizing	17	3
Lengua & Kovacs (2005)	92	Longitudinal	America	Both (54%)	7.8–11.9	9–13	–	Self: EATQ Parent: EATQ	Internalizing symptoms Externalizing symptoms	16	3
Levy et al. (2020)	1516	Cross-sectional	Canada	Both (74%)	9.00 (2.19)	–	–	Parent: OCHS-R	Suicidality	16	1
Liu et al. (2024)	535	Cross-sectional	China	Both (51%)	10.01 (1.42)	–	–	Self: EATQ-R	Depression	18	1
Loram et al., (2021)	82	Cross-sectional	Australia	Both (88%)	14.33 (1.38)	–	–	Parent: ARI Self: ARI	ADHD	18	1
Maire et al. (2020)	98	Cross-sectional	France	Both (81%)	7–11	–	–	Parent: CPRS-3	Inattention Hyperactivity Opposition Anxiety	16	1
Martin et al. (2017)	139	Cross-sectional	America	Both (76%)	4–5	–	–	Parent: DIPA	Emotionally reactive Anxious/depressed Withdrawn Attention problems Aggressive behavior ODD MDD ADHD Anxiety disorder PTSD	16	1
Mikolajewski et al. (2017)	2450	Longitudinal	America	Both (49%)	11.38	17	–	Parent: DICA-R	Internalizing symptoms MDD Externalizing symptoms Specific phobia Panic disorder Adult antisocial behavior Alcohol use disorder	17	3
Mulraney et al. (2014)	62	Cross-sectional	Australia	Both (39%)	15.29 (1.32)	–	–	Parent: ARI	Emotional problems Conduct problems Hyperactivity Peer problems Prosocial	12	1
Mulraney et al. (2017)	140	Longitudinal	Australia	Both (89%)	5–13	12–17	–	Parent: ARI Self: ARI	ADHD	14	3
Naim et al. (2021)	109	Cross-sectional	America	Both (72%)	8–18	–	–	Parent: ARI Self: ARI	DMDD ADHD Anxiety	16	1
Nelson et al. (2018)	69	Cross-sectional	America	Both (37%)	9–14	–	–	Parent: ARI Self: ARI	Anxiety Depression Stress	17	1
Pan & Yeh (2019)	97	Cross-sectional	Taiwan	Both (73%)	6–17	–	–	Parent: ARI Self: ARI	Aggression Anxiety/depression Social problems	15	1
Perhamus & Ostrov (2021)	300	Longitudinal	America	Both (56%)	3.7(0.37)	7	–	Parent: CBQ-TF	Reactive physical aggression Proactive physical aggression	18	2
Poznanski et al. (2018)	435	Cross-sectional	America	Both (47%)	12.7 (3.0)	–	–	Parent: CBCL	Anxiety severity Depressive disorder severity Sleep problems ADHD/DBD severity	17	1
Rappaport et al. (2020)	374	Cross-sectional	America	Not reported	9–14	–	–	Self: ARI	Depression GAD Separation anxiety Social anxiety Panic disorder symptoms	14	1
Rice et al. (2017)	337	Cross-sectional	England	Both (42%)	12.4 (2.00)	14	–	CAPA	Depression	15	1
Rowe et al. (2010)	1420	Longitudinal	America	Both (56%)	9–13	13–16	–	Parent: CAPA	Conduct Disorder ODD Substance use disorder Anxiety Depression	16	3
Rubens et al. (2017)	285	Cross-sectional	America	Both (48%)	8–11	–	–	Self: ARI	Anxiety Depression Emotion dysregulation Delinquency Reactive aggression Proactive aggression	15	1
Silver et al. (2021)	550	Longitudinal	America	Girls	14.37 (0.62)	+ 3 years	–	Self: IPIP, BPAQ, TAI	Depression Panic disorder GAD Social phobia Specific phobia ODD Conduct Disorder Substance use disorder	16	3
Silver et al. (2024)	418	Cross-sectional	America	Both (57%)	12.66 (0.46)	15.25 (0.41)	18.37 (0.54)	Self: ARI Parent: ARI	Suicidal ideation Depressive disorder Anxiety disorder DBD ADHD	20	1
Smith et al. (2019)	731	Longitudinal	America	Both (51%)	2–5	7.5 – 10.5	–	Parent: CBCL	Externalizing Internalizing ODD GAD MDD	14	2
Sorcher et al. (2022)	212	Longitudinal	America	Both (53%)	3.51 (0.26)	15.25 (0.40)	–	Parent: PAPA	Depression Anxiety Specific phobia Social phobia Separation anxiety GAD ADHD DBD Self-harm Suicidal ideation Global functioning	16	2
Srinivasan et al., (2024)	7225	Longitudinal	England	Both (49%)	3	5	14	SDQ CSBQ	Depressive symptoms	18	2
Stoddard et al. (2017)	115	Cross-sectional	England	Both (56%)	8–17	–	–	Parent: ARI	Anxiety	16	1
Stringaris & Goodman (2009a)	Total:18,298 4278 (Parent) 14,020 (Teacher)	Cross-sectional	England	Both (57%) Both (50%)	10.4 (3.3) 10.6 (3.2)	–	–	Parent: DAWBA Teacher: DAWBA	Emotional problems Hyperactivity Conduct problem Peer problems	18	1
Stringaris & Goodman (2009b)	7912	Longitudinal	England	Both (52%)	10.2 (3.3)	13.2 (3.3)	–	Parent: DAWBA	Internalizing disorders ADHD Conduct Disorder Distress disorders	16	3
Stringaris et al. (2012)	306	Longitudinal	England	Both (42%)	15	17	–	Self ASEBA	Depression Delinquency	18	5
Theriault et al. (2018)	58	Longitudinal	Canada	Both (85%)	10.3 (2.6)	19.7 (2.5)	–	Parent: DSM-IV clinician interview	Chronic tic and anxiety Chronic tic and compulsive symptoms	18	6
Ucar & Vural (2018)	86	Cross-sectional	Turkey	Both (68%)	13.72 (1.34)	–	–	Parent: ARI Self: ARI	ADHD	16	1
Valencia et al. (2021)	470	Longitudinal	Spain	Both (50%)	8	11	–	Parent: SDQ	Internalizing symptoms	17	2
Vogel et al. (2019)	302	Longitudinal	America	Both (52%)	3–5	16–19	–	Parent: PAPA	ADHD ODD Conduct disorder	14	3
Wakschlag et al. (2015)	497	Longitudinal	America	Both (50%)	2.9—6	3.1 – 7.7	3.8 – 8.5	Parent: MAP-DB Temper loss scale	ODD symptoms CD symptoms ADHD symptoms Depression symptoms GAD symptoms Social anxiety symptoms	19	2
Wakschlag et al. (2020)	151	Cross-sectional	America	Both (53%)	4.82 (0.60)	–	–	Parent: MAP-DB Temper loss scale	Internalizing Externalizing	15	1
Wang et al. (2023)	323	Longitudinal	China	Both (50%)	8.29 (0.47)	1 year later	2 years later	Parent: CBQ	GAD	18	3
Waschbusch et al. (2020)	219	Cross-sectional	America	Both (79%)	9.53 (1.57)	–	–	Parent & Teacher (Combined): DBDRS	Psychopathology Aggression Peer problems Impairment	14	1
Waxmonsky et al. (2022)	48	Cross-sectional	America	Both (69%)	8.08 (2.09)	–	–	Parent: ARI	ADHD ODD CD Callous-unemotional	17	1
Waxmonsky et al. (2017)	S1: 665 S2: 784 Total: 1449	Cross-sectional	America	S1: Both (53%) S2: Both (68%)	6–12	–	–	Parent: PBS	Oppositional behavior Hyperactive-Impulsive Conduct problem Anxiety Depression Inattention	17	1
Whelan et al. (2015)	3963	Longitudinal	United Kingdom	Not reported	2	10	13	Mother & Teacher: DAWBA	Anxiety/depression Conduct problems Depression at 16y	19	3
Wiggins et al. (2023)	425	Longitudinal	America	Both (49%)	4.7 (0.85)	7.1 (1.1)	9.3 (0.79)	Parent: MAPS-TL	Any disorder Externalizing Internalizing	16	2
Wilson et al. (2022)	115	Cross-sectional	America	Both (48%)	5.56 (1.7) 3–8	–	–	Parent: ARI	Anxiety Anxious/depressed Aggression Attention	17	1
Zendarski et al. (2023)	51	Cross-sectional	Australia	Both (53%)	11.17 (2.5)			Parent: ARI Self: ARI	Internalizing Externalizing ADHD ASD	18	1
Zhou et al. (2009)	S1: 382 S2: 322 Total: 704	Cross-Sectional	Chinese/USA	S1: Both (44%) S2: Both (47%)	S1: 11.6 (0.6) S2: 9.6 (1.0)	–	–	S1 Parent/ Teacher: CBQ S2 Parent/ Teacher: EATQ	Attention Externalizing Internalizing	18	1
Zik et al. (2022)	195	Cross-sectional	America	Both (59%)	13.16 (2.48)	–	–	Parent: ARI Self: ARI	Aggression Anger	16	1

*ARI* Affective Reactivity Index, *ASEBA Achenbach System of Empirically Based Assessment*, *BCFPI* Brief Child and Family Phone Interview, *BiTe* Brief Irritability Test, *BPAQ* Buss-Perry Aggression Questionnaire, *CAPA* The Child and Adolescent Psychiatric Assessment, *CASI* Child and Adolescent Symptom Inventory, *CBCL* Child Behavior Checklist, *CBCL-YSR* Child Behavior Checklist Youth Self Report, *CBQ-TF* Children's Behavior Questionnaire—Short Form, *CHI* Children Hostility Inventory, *CIS* Caprara Irritability Scale, *CPRS-R* Conners’ Parent Rating Scale, *CSBQ* Child Social Behavior Questionnaire, *CSI-IV* Child Symptom Inventory, *DAWBA* Development and Well-Being Assessment*, DBDC* Disruptive Behavior Disorder Checklist, *DBDRS* The Disruptive Behavior Disorders Rating Scale, *DICA-R* Diagnostic Interview for Children and Adolescents, *DIPA* Diagnostic Infant and Preschool Assessment, *DISC* Diagnostic Interview Schedule for Children, *EATQ* Early Adolescent Temperament Questionnaire, *HiPIC* The Hierarchical Personality Inventory for Children, IPIP = International Personality Item Pool, *K-SADS-P* Schedule for affective disorders and schizophrenia for school-age children-present episode, *K-SADS-PL* Schedule for affective disorders and schizophrenia for school-age children-present and lifetime version, *LAB-TAB* Laboratory Temperament Assessment Battery, *MAP-DP* Multidimensional Assessment of Preschool Disruptive Behavior, *MINI-KID* The Mini-International Neuropsychiatric Interview for Children and Adolescents, *OCHS-R* The Ontario Child Health Survey Scales-Revised, *PAPA* The Preschool Age Psychiatric Assessment, *PBS* Pediatric Behavior Scale, *SCAR* Screen for Children Affective Reactivity, *SDQ* Strengths and Difficulties Questionnaire, *SNAP* Swanson, Nolan, and Pelham rating scale, *TAI* Temperament and Affectivity Inventory. *Model Key: 1* Concurrent (all childhood), *2* Longitudinal (preschool to childhood), *3* Longitudinal (childhood to later childhood/adolescence), *4* Longitudinal (childhood to adulthood), *5* Longitudinal (adolescence to adolescence), *6* Longitudinal (adolescence to adulthood)

### Concurrent Associations (Model 1)

***Irritability and overall mental health symptoms.*** The analysis of studies reporting concurrent data showed a positive association between irritability and overall mental health symptoms, *k* = 417, *r* = 0.33 (95% CI 0.29, 0.36). Forest and Funnel plots are available as supplementary materials (Fig. S6a and S6b). Egger’s regression was non-significant, indicating no publication bias, *t*(415) = 1.23, *p* = 0.22. Heterogeneity was found across the models, *I*^*2*^ > 98, with a significant *Q* statistic, thereby supporting further tests of potential moderators. For studies reporting on concurrent associations, study quality was significant *k* = 417, *χ*^*2*^(1) = 4.45, *p* = 0.03, *R*^*2*^ = 0.01, indicating that as the study quality increased, the association between irritability and mental health decreased (see Supplementary file, Figure S6c). Other moderators were non-significant: age (*k* = 417, *χ*^*2*^(1) = 0.33, *p* = 0.56, *R*^*2*^ = 0.00); informant type (*k* = 417, *χ*^*2*^(5) = 9.92, *p* = 0.07, *R*^*2*^ = 0.02), and sex (*k* = 412, *χ*^*2*^(1) = 1.79, *p* = 0.18, *R*^*2*^ = 0.00). A sensitivity analysis was conducted to examine whether results varied according to measure of irritability. The fixed effects model (*k* = 417, *r* = 0.31 (95% CI 0.31, 0.31)) and the random effect models (*k* = 417, *r* = 0.28 (95% CI 0.26, 0.29)) did not differ significantly (*p* = 0.64), indicating that the measure of irritability did not significantly influence the results.

***Irritability and externalizing/internalizing symptoms*.** Analysis of concurrent data showed a positive association between irritability and internalizing symptoms *k* = 189, *r* = 0.29 (95% CI 0.27, 0.32), and a positive association with externalizing symptoms, *k* = 181, *r* = 0.37 (95% CI 0.31, 0.43). In meta-regression testing symptom dimensions as moderators, the effect size for externalizing symptoms was significantly greater than for internalizing symptoms *k* = 370, *χ*^2^(1) = 4.21, *p* = 0.04, *R*^*2*^ = 0.01 (see Supplementary file, Fig. S6d).

***Irritability and diagnostic domains.*** Subgroup analysis conducted on effect sizes for concurrent associations between irritability and diagnostic domains were as follows: anxiety, *k* = 73, *r* = 0.22 (95% CI 0.21, 0.23); depression, *k* = 47, *r* = 0.28 (95% CI 0.27, 0.29); ADHD, *k* = 77, *r* = 0.26 (95% CI 0.20, 0.31); ASD, *k* = 6, *r* = 0.10 (95% CI − 0.01, 0.21); CD, *k* = 23, *r* = 0.36 (95% CI 0.35, 0.37); ODD,* k* = 29, *r* = 0.63 (95% CI 0.63, 0.64); SUD *k* = 2, *r* = 0.49 (95% CI− 0.40, 0.58); and OCD,* k* = 5, *r* = 0.14 (95% CI 0.09, 0.18). Meta-regression showed that the effect size for ODD was significantly greater than other diagnostic domains, *k* = 260, *χ*^2^(6) = 94.25, *p* = 0.00, *R*^*2*^ = 0.28 (see Supplementary file, Fig. S6e).

### Longitudinal Associations: Preschool to Middle Childhood (Model 2)

***Irritability and overall mental health symptoms.*** The analysis of longitudinal studies showed a positive association between preschool irritability and overall mental health symptoms in middle childhood, *k* = 65, *r* = 0.21 (95% CI 0.18, 0.24). Forest and Funnel plots are available as supplementary materials (Figure S6f and S6g). Egger’s regression was significant, indicating publication bias, *t*(63) = 7.39, *p* = 0.00. Heterogeneity was found across the models, *I*^2^ > 85 and the *Q* statistic significant, thereby supporting tests of potential moderators. Age (*k* = 65, *χ*^2^(1) = 0.03, *p* = 0.87, *R*^*2*^ = 0.00), sex (*k* = 65, *χ*^2^(1) = 0.58, *p* = 45, *R*^*2*^ = 0.0), study quality (*k* = 65, *χ*^2^(1) = 1.27, *p* = 0.32, *R*^*2*^ = 0.01) and informant type (*k* = 65, *χ*^2^(3) = 5.60, *p* = 0.13, *R*^*2*^ = 0.01) were tested as moderators and found to be non-significant. Due to the small number of measures of irritability in these studies, a moderation analysis, rather than sensitivity analysis, was conducted to examine variation based on type of measure. The model was also non-significant (*k* = 65, *χ*^2^(6) = 11.32, *p* = 0.08, *R*^*2*^ = 0.18), indicating that factors other than measurement type, age, sex, study quality, and informant type may account for heterogeneity in the model.

***Irritability and externalizing/internalizing symptoms*.** A subgroup analysis of preschool irritability and mental health outcomes in middle childhood indicated comparable effect sizes for internalizing symptoms *k* = 26, *r* = 0.18 (95% CI 0.13, 0.22) and externalizing symptoms, *k* = 30, *r* = 0.23 (95% CI 0.18, 0.27). Meta-regression analysis testing these symptom dimensions as moderators showed no significant difference (*k* = 56, *χ*^2^(1) = 2.64, *p* = 0.10, *R*^*2*^ = 0.05).

***Irritability and diagnostic domains*.** In a subgroup analysis conducted on the association between preschool irritability and middle childhood diagnostic domains, effect sizes were as follows: anxiety* k* = 8, *r* = 0.11 (95% CI 0.07, 0.14); depression, *k* = 9, *r* = 0.13 (95% CI 0.06, 0.20); ADHD, *k* = 9, *r* = 0.16 (95% CI 0.10, 0.23); CD, *k* = 6, *r* = 0.22 (95% CI 0.10, 0.35); and ODD,* k* = 6, *r* = 0.25 (95% CI 0.19, 0.31). Meta-regression found a significant difference across diagnostic domains (*k* = 38, *χ*^2^(1) = 11.59, *p* = 0.02, *R*^*2*^ = 0.29), whereby effect sizes for CD were larger than for anxiety (see Supplementary file, Fig. S6h).

### Longitudinal Associations: Middle Childhood to Late Childhood/Adolescence (Model 3)

***Irritability and overall mental health symptoms*.** This analysis showed a positive association between irritability in middle childhood and mental health symptoms in later childhood/adolescence, *k* = 39, *r* = 0.14 (95% CI 0.11, 0.17). Forest and Funnel plots are available as supplementary materials (Figure S6i and S6j). Egger’s regression was significant, indicating publication bias, *t*(37) = 2.54, *p* = 0.00. Heterogeneity was found across the models, *I*^*2*^ > 85 and the *Q* statistic significant, thereby supporting tests of potential moderators. Age (*k* = 39, *χ*^2^(1) = 0.56, *p* = 0.46, *R*^*2*^ = 0.02), sex (*k* = 38, *χ*^2^(1) = 0.52, *p* = 0.47, *R*^*2*^ = 0.00), study quality (*k* = 39, *χ*^2^(1) = 3.63, *p* = 0.06, *R*^*2*^ = 0.11), and informant type (*k* = 34, *χ*^2^(1) = 3.88, *p* = 0.27, *R*^*2*^ = 0.08), were tested as moderators and found to be non-significant. Due to the small number of measures of irritability in these studies, a moderation analysis, rather than sensitivity analysis, was conducted to examine variation based on type of measure. The model was significant (*k* = 29, *χ*^2^(9) = 26.64, *p* = 0.00, *R*^*2*^ = 0.55), indicating that effect sizes for studies using the Affective Reactivity Index (ARI) were significantly larger than those using the CBQ, DAWBA, DICA, DISC, the Disruptive Behavior Disorder Checklist (DBDC), the Child and Adolescent Psychiatric Assessment (CAPA), and the Strengths and Difficulties Questionnaire (SDQ) (see Supplementary file, Figure S6k).

***Irritability and externalizing/internalizing symptoms***. Effect sizes were as follows for externalizing, *k* = 10, *r* = 0.13 (95% CI 0.03, 0.22), and internalizing symptom dimensions, *k* = 25, *r* = 0.14 (95% CI 0.10, 0.18). Meta-regression analysis testing these symptom dimensions as moderators showed no significant difference (*k* = 35, *χ*^2^(1) = 0.04, *p* = 0.85, *R*^*2*^ = 0.00).

***Irritability and diagnostic domains***. In a subgroup analysis conducted on the association between middle childhood irritability and diagnostic domains in later childhood/adolescence, effect sizes were as follows: anxiety,* k* = 12, *r* = 0.14 (95% CI 0.08, 0.19); depression, *k* = 13, *r* = 0.15 (95% CI 0.09, 0.20); CD, *k* = 4, *r* = 0.14 (95% CI − 16., 0.41); and ODD,* k* = 4, *r* = 0.16 (95% CI 0.04, 0.26). Meta-regression indicated no significant differences between diagnoses *k* = 36, *χ*^2^(5) = 0.30, *p* = 0.99, *R*^*2*^ = 0.01.

### Longitudinal Associations: Middle Childhood to Adulthood (Model 4)

***Irritability and overall mental health symptoms***. This model could not be run due to insufficient data. Available data were integrated with model 6.

### Longitudinal Associations: Early Adolescence to Late Adolescence (Model 5)

***Irritability and overall mental health symptoms*.** A positive association was found between irritability in early adolescence and mental health symptoms in later adolescence, *k* = 11, *r* = 0.21 (95% CI 0.15, 0.28). Forest and Funnel plots are available as Supplementary materials (Fig. S6l and S6m). Egger’s regression was significant, indicating potential publication bias, *t*(9) = 2.95, *p* = 0.02. Heterogeneity across the models,* I*^*2*^ > 60, and a significant *Q* statistic, supported moderation analysis. Age was found to be a significant moderator, *k* = 11, *χ*^2^(1) = 12.00, *p* = 0.00, *R*^*2*^ = 0.93 (see Supplementary file, Fig. S6n), such that as age increased, the effect size for overall mental health symptoms was larger. Informant type (youth self-report; parent report) was significant, *k* = 11, *χ*^2^(1) = 12.00, *p* = 0.00, *R*^*2*^ = 0.93 (see Supplementary file, Fig. S6o), whereby effect sizes for youth self-reports were larger than parent-reports. A significant moderation effect was also found for sex, *k* = 11, *χ*^2^(1) = 8.35, *p* = 0.00, *R*^*2*^ = 0.88, indicating smaller effects for samples with more boys (see Supplementary file, Fig. S6p). No moderator effect was found for study quality (*k* = 11, *χ*^2^(1) = 1.00, *p* = 0.32, *R*^*2*^ = 0.27).

Due to the small number of measures of irritability in these studies, a moderation analysis, rather than sensitivity analysis, was conducted to examine variation based on type of measure. The model was significant (*k* = 11, *χ*^2^(2) = 12.02, *p* = 0.00, *R*^2^ = 0.93), indicating that effect sizes for studies using the ASEAB were significantly greater than those using the DISC in this age group (see Supplementary file, Figure S6q).

***Irritability and externalizing/internalizing symptoms*.** Effect sizes were as follows for externalizing, *k* = 4, *r* = 0.27 (95% CI 0.23, 0.31) and internalizing symptom dimensions, *k* = 5, *r* = 0.17 (95% CI 0.03, 0.31). The number of available studies was not sufficient for meta-regression analysis.

***Irritability and diagnostic domains***. In a subgroup analysis conducted on the association between irritability and diagnostic domains, effect sizes were as follows: anxiety, *k* = 3, *r* = 0.11 (95% CI − 0.02, 0.38); depression, *k* = 2, *r* = 0.27 (95% CI 0.09, 0.43); ADHD *k* = 2, *r* = 0.17 (95% CI − 0.06, 0.38); CD, *k* = 1, *r* = 0.19 (95% CI − 16, 0.41); and ODD,* k* = 2, *r* = 0.25 (95% CI − 0.02, 0.49). A meta-regression conducted with ODD as the intercept indicated a significant difference compared to anxiety *k* = 10, *χ*^2^(4) = 19.16, *p* = 0.01, *R*^*2*^ = 1.00 (see Supplementary File S6r).

### Longitudinal Associations: Adolescence to Adulthood (Model 6)

***Irritability and overall mental health symptoms*.** A positive association was found between irritability in adolescence and mental health symptoms in adulthood, *k* = 9, *r* = 0.25 (95% CI 0.12, 0.37). Forest and Funnel plots are available as Supplementary materials (Figure S6s and S6t). Egger’s regression was significant, suggesting publication bias, *t*(7) = 2.23, *p* = 0.06. The number of available studies was not sufficient for meta-regression analysis.

***Irritability and externalizing/internalizing symptoms*.** Effect sizes were as follows for externalizing, *k* = 3, *r* = 0.39 (95% CI 0.32, 0.46), internalizing symptom dimensions, *k* = 5, *r* = 0.05 (95% CI − 0.00, 0.09). The number of available studies was not sufficient for meta-regression analysis.

***Irritability and diagnostic domains*.** No subgroup analysis was conducted due to insufficient data.

## Discussion

Evidence regarding irritability and mental health has grown rapidly in recent years, and the current meta-analysis is the first to examine irritability across childhood and adolescence as it relates to mental health symptoms during these periods. Findings demonstrated a significant and positive association between irritability and severity of concurrent overall psychopathology in the order of a moderate effect size, while small to moderate effect sizes characterized the association between irritability and later mental health outcomes in studies with prospective designs. Notwithstanding the significant relationship between irritability and overall severity of mental health symptoms, this association was also found to differ somewhat according to symptom type. This was evident both at the level of broad dimensions of internalizing versus externalizing symptoms, as well as symptom domains based on more specific diagnostic categories.

The association between irritability and concurrent problem severity was most pronounced for externalizing problems, which demonstrated a significantly greater association with irritability than internalizing problems in concurrent data. Data on concurrent associations are particularly relevant to the conceptualization of irritability in diagnostic models, especially models of disorders in which irritability forms a core phenotypic feature (Evans et al., 2021a). When externalizing problems were broken down further into more distinct diagnostic domains, symptoms of ODD showed the most pronounced association with irritability, relative to other diagnostic domains including CD. This is understandable given that diagnostic criteria for ODD include an angry/irritable mood symptom dimension (APA, 2013), and factor analytic research has provided strong support for the inclusion of this dimension in the overall structure of ODD (Burke et al., 2014).

Prospectively, irritability was associated with greater mental health symptoms across each of the developmental periods examined. Consistent with the data on concurrent associations, and the conceptualization of irritability as a transdiagnostic construct, these positive longitudinal associations were seen for both externalizing and internalizing symptoms. Prospective associations between irritability and each of these broad symptom dimensions were highly comparable, yet these prospective associations were once again more prominent for diagnostic features of externalizing problems, in terms of ODD and CD specifically. This is noteworthy given that current diagnostic criteria for CD do not explicitly refer to irritability. It is nonetheless consistent with evidence regarding the highly overlapping nature of the symptoms represented by ODD and CD, which have been conceptualized as part of a broader externalizing spectrum also encompassing ADHD, substance use disorder, and antisocial personality disorder. Although irritability is often not explicitly named in the diagnostic criteria for these disorders, all are understood to be associated with irritability. Moreover, irritability is implicated in the common liabilities from which these disorders are thought to arise, such as trait impulsivity (Lahey et al., 2011). In terms of the neurodevelopmental underpinnings of such impulsivity, it has been proposed that irritability reflects a chronically aversive mood state driven by subcortical dysfunction in the mesolimbic dopamine system, which in turn motivates individuals to engage in reward-seeking behaviors (Beauchaine et al., 2017).

As a transdiagnostic factor that is understood to reflect emotion dysregulation, irritability may help explain the high rates of comorbidity between diverse forms of internalizing and externalizing disorders in the population. CD for example, frequently co-occurs with depression and anxiety, particularly among girls (Fairchild et al., 2019). Emerging research has pointed to mechanisms that may account for the diverse forms of psychopathology that arise from the common starting point of irritability, reflecting the principle of multifinality in developmental psychopathology. For example, variations in stress system functioning (diurnal cortisol slopes) have been found to differentiate developmental trajectories of early childhood irritability that lead to internalizing versus externalizing symptoms in early adolescence (Kessel et al., 2021).

Interestingly, in our data the specific pattern of prospective associations with diagnostic features varied somewhat across distinct developmental periods, such that early childhood irritability was more strongly associated with features of CD across middle childhood, whereas irritability in early adolescence was most strongly associated with features of ODD across adolescence. This raises important questions regarding developmental changes in the relationship between irritability and distinct risk pathways over time. These findings could be seen as analogous to the notion that distinct dimensions of ODD symptoms, including irritable mood, may vary in relation to one another across development, as has been subject to some speculation (Burke & Loeber, 2010). Although research testing this directly has generally supported the structural invariance of ODD dimensions across development (Jungersen & Lonigan, 2022; Lavigne et al., 2015), further investigation is warranted. There is a particular need for further longitudinal studies of irritability in late childhood and early adolescence as a predictor of subsequent mental health symptoms across adolescence and early adulthood, given that the number of such studies currently available precluded tests of moderation by symptom type across these periods.

In addition to the developmental perspective provided by these results, our tests of age as a moderator variable provided some evidence that irritability may become more strongly associated with overall mental health problems as children and adolescents get older. Specifically, within adolescence (age range 13–18 years), an older age was associated with a larger effect size. This should be interpreted with caution, however, as it may in part reflect that the testing interval for older adolescents would have been shorter than for younger adolescents in this model. Based on the broad range of neurocognitive domains that have been implicated in irritability-related risk mechanisms (e.g., cognitive control; reward processing; Elvin et al., 2024), it is important to consider how cog-nitive development may influence the relationship between irritability and symptoms of psychopathology. Recent research has identified developmental trajectories of irritability that appear to be differentially associated with clinical outcomes, and there is a need for further developmental research into the multilevel (e.g., cognitive, genetic, environment) mechanisms underlying these trajectories (Leibenluft et al., 2024).

Our search indicated a need for research to investigate irritability as a distinct treatment outcome variable, which remains limited to date. Considerable support is nonetheless available for the effects of parenting interventions on irritability within the symptom profile of ODD (Hawes et al., 2023, Stringaris et al., 2018). Recent research has also supported the potential for such intervention, and cognitive behavior therapy, to reduce transdiagnostic features of irritability in the context of modular approaches (Evans et al., 2021b). Emerging evidence regarding interplay between child characteristics and environmental factors such as quality of parenting further suggests that irritability may be implicated in a temperament profile associated with differential susceptibility to the environment (Belsky et al., 2007). For example, children with a profile characterized by temperamental traits of high irritability, approach, activity, and impulsivity appear to be more vulnerable to externalizing problems when exposed to negative maternal parenting, compared to children without this profile, but these same children also appear to benefit more than other children when exposed to positive maternal parenting (Hentges et al., 2023). Moreover, irritability may in some instances reflect a vantage sensitivity, accounting for individual differences in response to parenting interventions (de Villiers et al., 2018).

The findings of our meta-analysis should be interpreted in light of some limitations. First, the studies from which data were extracted were conducted largely in Western, educated, industrialized, rich, and democratic (WEIRD) populations (Henrich et al., 2010), and therefore generalizability of results across cultures and regions is unclear. Populations from Asia and Africa were particularly under-represented. Second, the funnel plots produced for our models indicated some risk of publication bias, suggesting that the overall effect sizes produced may be somewhat inflated. Third, like the meta-analytic review by Finlay-Jones et al. (2024), our literature search was limited to English-language publications. Finally, heterogeneity estimates were high for many of the models tested, which may be due to diverse measures of irritability used across studies, in addition to inconsistent approaches to controlling for factors such as age, sex, and baseline mental health symptoms. Notwithstanding these limitations, our design and methodology reflects strengths including a specific focus on studies that have indexed irritability in isolation from overlapping constructs such as negative emotionality, and the examination of the construct across childhood and adolescence. One of the most common measures of irritability across these studies was the Affective Reactivity Index, as recognized in DSM-5 as a gold-standard measure of the construct, and our meta-analysis is the first to include the extensive child and adolescent research that has been conducted with this measure in the past decade.

## Conclusions

In conclusion, our findings demonstrate a positive association between child and adolescent irritability and symptoms spanning internalizing, externalizing, and neurodevelopmental disorders, supporting conceptualizations of irritability as a transdiagnostic form of emotion dysregulation (Beauchaine & Tackett, 2020; Klein et al., 2021; Leibenluft et al., 2024). These findings are also consistent with recent meta-analytic evidence regarding irritability in infancy and early childhood, which was used to argue that early irritability represents a marker for neurodevelopmental vulnerability to mental health problems later in life (Finlay-Jones et al., 2024). Our findings regarding the relative association between irritability and distinct mental health symptoms, particularly features of ODD and CD, have important implications for developmental and diagnostic models of psychopathology, and the conceptualization of emotion dysregulation in such problems. At the same time, much remains to be learned about the mechanisms through which irritability confers risk for psychopathology across development, including the transactional dynamics by which irritability and contextual factors may shape one another over time, as well as intervention practices for targeting irritability in its own right.

## Supplementary Information

Below is the link to the electronic supplementary material.

Supplementary file1 (DOCX 6797 KB)
