# Supplementary material for: Irritability as a Transdiagnostic Construct Across Childhood and Adolescence: A Systematic Review and Meta-analysis
Source: Clin Child Fam Psychol Rev. 2025 Jan 20;28(1):101–24. doi: 10.1007/s10567-024-00512-4 (PMC11885387; doi:10.1007/s10567-024-00512-4)
Supplement: Supplementary file 1 — Supplementary file1 (DOCX 6797 KB) [file 10567_2024_512_MOESM1_ESM.docx]

Supplementary Materials

**Irritability and Psychopathology Across Childhood and Adolescence: A Systematic Review and Meta-analysis**

This document contains the following materials:

Table S1: PRISMA 2020 Checklist

Table S2: Quality of study

Table S3: Excluded studies

Table S4: Funding declarations for included studies

Table S5: Search strategies and results from individual databases

Figures S6: Figures of Forrest Plots, Funnel Plots, and Scatterplots

References for included studies.

**Table S1:**

*PRISMA 2020 Checklist*

| Section and Topic | Item # | Checklist item | Included |  |  |
| --- | --- | --- | --- | --- | --- |
| TITLE | | |  |  |  |
| Title | 1 | Identify the report as a systematic review. | ☑ |  |  |
| ABSTRACT | | |  |  |  |
| Abstract | 2 | See the PRISMA 2020 for Abstracts checklist. | ☑ |  |  |
| INTRODUCTION | | |  |  |  |
| Rationale | 3 | Describe the rationale for the review in the context of existing knowledge. | ☑ |  |  |
| Objectives | 4 | Provide an explicit statement of the objective(s) or question(s) the review addresses. | ☑ |  |  |
| METHODS | | |  |  |  |
| Eligibility criteria | 5 | Specify the inclusion and exclusion criteria for the review and how studies were grouped for the syntheses. | ☑ |  |  |
| Information sources | 6 | Specify all databases, registers, websites, organisations, reference lists and other sources searched or consulted to identify studies. Specify the date when each source was last searched or consulted. | ☑ |  |  |
| Search strategy | 7 | Present the full search strategies for all databases, registers and websites, including any filters and limits used. | ☑ |  |  |
| Selection process | 8 | Specify the methods used to decide whether a study met the inclusion criteria of the review, including how many reviewers screened each record and each report retrieved, whether they worked independently, and if applicable, details of automation tools used in the process. | ☑ |  |  |
| Data collection process | 9 | Specify the methods used to collect data from reports, including how many reviewers collected data from each report, whether they worked independently, any processes for obtaining or confirming data from study investigators, and if applicable, details of automation tools used in the process. | ☑ |  |  |
| Data items | 10a | List and define all outcomes for which data were sought. Specify whether all results that were compatible with each outcome domain in each study were sought (e.g. for all measures, time points, analyses), and if not, the methods used to decide which results to collect. | ☑ |  |  |
|  | 10b | List and define all other variables for which data were sought (e.g. participant and intervention characteristics, funding sources). Describe any assumptions made about any missing or unclear information. | ☑ |  |  |
| Study risk of bias assessment | 11 | Specify the methods used to assess risk of bias in the included studies, including details of the tool(s) used, how many reviewers assessed each study and whether they worked independently, and if applicable, details of automation tools used in the process. | ☑ |  |  |
| Effect measures | 12 | Specify for each outcome the effect measure(s) (e.g. risk ratio, mean difference) used in the synthesis or presentation of results. | ☑ |  |  |
| Synthesis methods | 13a | Describe the processes used to decide which studies were eligible for each synthesis (e.g. tabulating the study intervention characteristics and comparing against the planned groups for each synthesis (item #5)). | ☑ |  |  |
|  | 13b | Describe any methods required to prepare the data for presentation or synthesis, such as handling of missing summary statistics, or data conversions. | ☑ |  |  |
|  | 13c | Describe any methods used to tabulate or visually display results of individual studies and syntheses. | ☑ |  |  |
|  | 13d | Describe any methods used to synthesize results and provide a rationale for the choice(s). If meta-analysis was performed, describe the model(s), method(s) to identify the presence and extent of statistical heterogeneity, and software package(s) used. | ☑ |  |  |
|  | 13e | Describe any methods used to explore possible causes of heterogeneity among study results (e.g. subgroup analysis, meta-regression). | ☑ |  |  |
|  | 13f | Describe any sensitivity analyses conducted to assess robustness of the synthesized results. | ☑ |  |  |
| Reporting bias assessment | 14 | Describe any methods used to assess risk of bias due to missing results in a synthesis (arising from reporting biases). | ☑ |  |  |
| Certainty assessment | 15 | Describe any methods used to assess certainty (or confidence) in the body of evidence for an outcome. | ☑ |  |  |
| RESULTS | | |  |  | ☑ |
| Study selection | 16a | Describe the results of the search and selection process, from the number of records identified in the search to the number of studies included in the review, ideally using a flow diagram. | ☑ |  |  |
|  | 16b | Cite studies that might appear to meet the inclusion criteria, but which were excluded, and explain why they were excluded. | ☑ |  |  |
| Study characteristics | 17 | Cite each included study and present its characteristics. | ☑ |  |  |
| Risk of bias in studies | 18 | Present assessments of risk of bias for each included study. | ☑ |  |  |
| Results of individual studies | 19 | For all outcomes, present, for each study: (a) summary statistics for each group (where appropriate) and (b) an effect estimate and its precision (e.g. confidence/credible interval), ideally using structured tables or plots. | ☑ |  |  |
| Results of syntheses | 20a | For each synthesis, briefly summarise the characteristics and risk of bias among contributing studies. | ☑ |  |  |
|  | 20b | Present results of all statistical syntheses conducted. If meta-analysis was done, present for each the summary estimate and its precision (e.g. confidence/credible interval) and measures of statistical heterogeneity. If comparing groups, describe the direction of the effect. | ☑ |  |  |
|  | 20c | Present results of all investigations of possible causes of heterogeneity among study results. | ☑ |  |  |
|  | 20d | Present results of all sensitivity analyses conducted to assess the robustness of the synthesized results. | ☑ |  |  |
| Reporting biases | 21 | Present assessments of risk of bias due to missing results (arising from reporting biases) for each synthesis assessed. | ☑ |  |  |
| Certainty of evidence | 22 | Present assessments of certainty (or confidence) in the body of evidence for each outcome assessed. | ☑ |  |  |
| DISCUSSION | | |  |  | ☑ |
| Discussion | 23a | Provide a general interpretation of the results in the context of other evidence. | ☑ |  |  |
|  | 23b | Discuss any limitations of the evidence included in the review. | ☑ |  |  |
|  | 23c | Discuss any limitations of the review processes used. | ☑ |  |  |
|  | 23d | Discuss implications of the results for practice, policy, and future research. | ☑ |  |  |
| OTHER INFORMATION | | |  |  |  |
| Registration and protocol | 24a | Provide registration information for the review, including register name and registration number, or state that the review was not registered. | ☑ |  |  |
|  | 24b | Indicate where the review protocol can be accessed, or state that a protocol was not prepared. | ☑ |  |  |
|  | 24c | Describe and explain any amendments to information provided at registration or in the protocol. | ☑ |  |  |
| Support | 25 | Describe sources of financial or non-financial support for the review, and the role of the funders or sponsors in the review. | ☑ |  |  |
| Competing interests | 26 | Declare any competing interests of review authors. | ☑ |  |  |
| Availability of data, code and other materials | 27 | Report which of the following are publicly available and where they can be found: template data collection forms; data extracted from included studies; data used for all analyses; analytic code; any other materials used in the review. | ☑ |  |  |

**Table S2:**

*Quality of Study*

|  | Questions (scored as 0 ‘no’ or 1 ‘yes’) | | | | | | | | | | | | | | | | | | | |  |
| --- | --- | --- | --- | --- | --- | --- | --- | --- | --- | --- | --- | --- | --- | --- | --- | --- | --- | --- | --- | --- | --- |
| Authors | 1 | 2 | 3 | 4 | 5 | 6 | 7 | 8 | 9 | 10 | 11 | 12 | 13 | 14 | 15 | 16 | 17 | 18 | 19 | 20 | Total |
| Aebi et al. (2016) | 1 | 1 | 1 | 1 | 1 | 1 | 1 | 1 | 1 | 1 | 1 | 1 | 0 | 1 | 1 | 1 | 1 | 1 | 0 | 1 | 18 |
| Aebi et al. (2013) | 1 | 1 | 1 | 1 | 1 | 1 | 1 | 1 | 1 | 1 | 1 | 1 | 1 | 1 | 1 | 1 | 1 | 1 | 0 | 0 | 18 |
| Ali et al. (2022) | 1 | 1 | 1 | 1 | 1 | 1 | 0 | 1 | 1 | 1 | 1 | 1 | 0 | 0 | 1 | 1 | 1 | 1 | 1 | 0 | 16 |
| Althoff et al. (2014) | 1 | 1 | 1 | 1 | 1 | 1 | 0 | 1 | 1 | 1 | 1 | 0 | 0 | 0 | 1 | 1 | 1 | 1 | 0 | 1 | 15 |
| Ambrosini et al. (2013) | 1 | 1 | 1 | 1 | 1 | 1 | 0 | 1 | 1 | 1 | 1 | 1 | 0 | 0 | 1 | 1 | 1 | 1 | 0 | 0 | 15 |
| Arana et al. (2021) | 1 | 1 | 1 | 1 | 1 | 1 | 1 | 1 | 1 | 1 | 1 | 1 | 0 | 0 | 1 | 1 | 1 | 1 | 0 | 1 | 17 |
| Barclay et al. (2022) | 1 | 1 | 1 | 1 | 1 | 1 | 0 | 1 | 1 | 1 | 1 | 1 | 0 | 0 | 1 | 1 | 1 | 1 | 0 | 1 | 16 |
| Barker & Salekin (2012) | 1 | 1 | 1 | 1 | 1 | 1 | 0 | 1 | 1 | 1 | 1 | 0 | 0 | 0 | 1 | 1 | 1 | 1 | 0 | 1 | 15 |
| Baweja et al. (2021) | 1 | 1 | 1 | 1 | 1 | 1 | 0 | 1 | 1 | 1 | 1 | 1 | 0 | 0 | 1 | 1 | 1 | 1 | 1 | 1 | 17 |
| Bell et al. (2023) | 1 | 1 | 1 | 1 | 1 | 1 | 0 | 1 | 1 | 1 | 1 | 1 | 1 | 0 | 1 | 1 | 1 | 1 | 1 | 1 | 18 |
| Benarous et al. (2020a) | 1 | 1 | 0 | 1 | 1 | 1 | 0 | 1 | 1 | 1 | 1 | 1 | 0 | 0 | 1 | 1 | 1 | 1 | 1 | 0 | 15 |
| Benarous et al. (2020b) | 1 | 1 | 1 | 1 | 1 | 1 | 0 | 1 | 1 | 1 | 1 | 1 | 0 | 0 | 1 | 1 | 1 | 1 | 1 | 1 | 17 |
| Bielas et al. (2016) | 1 | 1 | 1 | 1 | 0 | 0 | 1 | 1 | 1 | 1 | 1 | 1 | 0 | 1 | 1 | 1 | 1 | 1 | 0 | 1 | 16 |
| Bolhius et al. (2017) | 1 | 1 | 1 | 1 | 1 | 1 | 0 | 1 | 1 | 1 | 1 | 1 | 0 | 0 | 1 | 1 | 1 | 1 | 1 | 1 | 17 |
| Brandes et al. (2019) | 1 | 1 | 1 | 1 | 1 | 1 | 0 | 1 | 1 | 1 | 1 | 1 | 0 | 0 | 1 | 1 | 1 | 1 | 0 | 1 | 16 |
| Burke (2012) | 1 | 1 | 1 | 1 | 1 | 1 | 0 | 1 | 1 | 1 | 1 | 0 | 0 | 0 | 1 | 1 | 1 | 1 | 0 | 1 | 15 |
| Burke et al (2014) | 1 | 1 | 1 | 1 | 1 | 1 | 0 | 1 | 1 | 1 | 1 | 1 | 0 | 0 | 1 | 1 | 1 | 1 | 0 | 0 | 15 |
| Busch et al. (2023) | 1 | 1 | 1 | 1 | 1 | 1 | 0 | 1 | 1 | 1 | 1 | 1 | 1 | 0 | 1 | 1 | 1 | 1 | 1 | 0 | 17 |
| Caprara et al. (2017) | 1 | 1 | 1 | 1 | 1 | 1 | 1 | 1 | 1 | 1 | 1 | 1 | 0 | 0 | 1 | 1 | 1 | 1 | 0 | 1 | 17 |
| Cardinale et al. (2021) | 1 | 1 | 1 | 1 | 1 | 1 | 0 | 1 | 1 | 1 | 1 | 1 | 0 | 0 | 1 | 1 | 1 | 1 | 1 | 1 | 17 |
| Cardinale et al. (2019) | 1 | 1 | 1 | 1 | 1 | 1 | 0 | 1 | 1 | 1 | 1 | 1 | 1 | 0 | 0 | 1 | 1 | 1 | 1 | 1 | 17 |
| Carter-Leno et al. (2021) | 1 | 1 | 1 | 1 | 1 | 1 | 0 | 1 | 1 | 1 | 1 | 1 | 0 | 0 | 1 | 1 | 1 | 1 | 1 | 1 | 17 |
| Chad-Friedman et al. (2023) | 1 | 1 | 1 | 1 | 1 | 1 | 0 | 1 | 1 | 1 | 1 | 1 | 0 | 0 | 1 | 1 | 1 | 1 | 1 | 1 | 17 |
| Chad-Friedman et al. (2022) | 1 | 1 | 1 | 1 | 1 | 1 | 1 | 1 | 1 | 1 | 1 | 1 | 1 | 1 | 1 | 1 | 1 | 1 | 1 | 1 | 20 |
| Chen et al. (2021) | 1 | 1 | 1 | 1 | 1 | 1 | 0 | 1 | 1 | 1 | 1 | 1 | 0 | 0 | 1 | 1 | 1 | 1 | 1 | 1 | 17 |
| Copeland et al. (2015) | 1 | 1 | 1 | 1 | 1 | 1 | 0 | 1 | 1 | 1 | 1 | 0 | 0 | 0 | 1 | 1 | 1 | 0 | 1 | 1 | 15 |
| Courbet et al. (2021) | 1 | 1 | 1 | 1 | 1 | 1 | 1 | 1 | 1 | 1 | 1 | 1 | 0 | 1 | 1 | 1 | 1 | 1 | 1 | 1 | 19 |
| Craig et al. (2021) | 1 | 1 | 1 | 1 | 1 | 1 | 1 | 1 | 1 | 1 | 1 | 1 | 0 | 1 | 1 | 1 | 1 | 1 | 1 | 1 | 19 |
| Degroot et al. (2024) | 1 | 1 | 1 | 1 | 1 | 1 | 0 | 1 | 1 | 1 | 1 | 1 | 1 | 0 | 1 | 1 | 1 | 1 | 1 | 0 | 17 |
| Déry et al. (2017) | 1 | 1 | 1 | 1 | 1 | 1 | 1 | 1 | 1 | 1 | 1 | 1 | 0 | 1 | 1 | 1 | 1 | 1 | 0 | 1 | 18 |
| Doerfler et al. (2020) | 1 | 1 | 1 | 1 | 1 | 1 | 0 | 1 | 1 | 1 | 1 | 1 | 0 | 0 | 1 | 1 | 1 | 1 | 1 | 1 | 17 |
| Dougherty et al. (2015) | 1 | 1 | 1 | 1 | 1 | 1 | 1 | 1 | 1 | 1 | 1 | 1 | 0 | 1 | 1 | 1 | 1 | 1 | 0 | 1 | 18 |
| Dougherty et al. (2016) | 1 | 1 | 1 | 1 | 1 | 1 | 1 | 1 | 1 | 1 | 1 | 1 | 0 | 1 | 1 | 1 | 1 | 1 | 0 | 1 | 18 |
| Dougherty et al. (2013) | 1 | 1 | 1 | 1 | 1 | 1 | 1 | 1 | 1 | 1 | 1 | 1 | 0 | 1 | 1 | 1 | 1 | 1 | 0 | 1 | 18 |
| Drabick & Gadow (2012) | 1 | 1 | 1 | 1 | 1 | 1 | 0 | 1 | 1 | 1 | 1 | 0 | 1 | 0 | 1 | 1 | 1 | 1 | 1 | 1 | 17 |
| Dugre & Potvin, (2020) | 1 | 1 | 1 | 1 | 1 | 1 | 1 | 1 | 1 | 1 | 1 | 0 | 1 | 1 | 1 | 1 | 1 | 1 | 0 | 0 | 17 |
| Elvin et al. (2021) | 1 | 1 | 1 | 1 | 1 | 1 | 0 | 1 | 1 | 1 | 1 | 1 | 0 | 0 | 1 | 1 | 1 | 1 | 1 | 1 | 17 |
| Elvin, Waters & Modecki (2023) | 1 | 1 | 1 | 1 | 1 | 1 | 0 | 1 | 1 | 1 | 1 | 1 | 0 | 0 | 1 | 1 | 1 | 1 | 0 | 1 | 16 |
| Evans et al. (2021) | 1 | 1 | 1 | 1 | 1 | 1 | 0 | 1 | 1 | 1 | 1 | 1 | 0 | 0 | 1 | 1 | 1 | 1 | 0 | 1 | 16 |
| Evans et al. (2020a) | 1 | 1 | 1 | 1 | 1 | 1 | 1 | 1 | 1 | 1 | 1 | 1 | 0 | 0 | 1 | 1 | 1 | 1 | 0 | 1 | 17 |
| Evans et al. (2020b) | 1 | 1 | 1 | 1 | 1 | 1 | 1 | 1 | 1 | 1 | 1 | 1 | 0 | 0 | 1 | 1 | 1 | 1 | 1 | 1 | 18 |
| Evans et al. (2020c) | 1 | 1 | 1 | 1 | 1 | 1 | 1 | 1 | 1 | 1 | 1 | 1 | 0 | 1 | 1 | 1 | 1 | 1 | 1 | 0 | 18 |
| Evans et al. (2023) | 1 | 1 | 1 | 1 | 1 | 1 | 0 | 1 | 1 | 1 | 1 | 1 | 0 | 0 | 1 | 1 | 1 | 1 | 1 | 1 | 17 |
| Evans et al. (2016) | 1 | 1 | 1 | 1 | 1 | 1 | 0 | 1 | 1 | 1 | 1 | 1 | 0 | 0 | 1 | 1 | 1 | 1 | 0 | 1 | 16 |
| Eyre et al. (2019) | 1 | 1 | 1 | 1 | 1 | 1 | 1 | 1 | 1 | 1 | 1 | 1 | 0 | 1 | 1 | 1 | 1 | 1 | 0 | 1 | 18 |
| Eyre et al. (2017) | 1 | 1 | 1 | 1 | 1 | 1 | 1 | 1 | 1 | 1 | 0 | 0 | 1 | 0 | 1 | 1 | 1 | 1 | 1 | 1 | 17 |
| Ezpeleta et al. (2012) | 1 | 1 | 1 | 1 | 1 | 1 | 0 | 1 | 1 | 1 | 1 | 1 | 0 | 0 | 1 | 1 | 1 | 1 | 1 | 1 | 17 |
| Ezpeleta et al. (2019) | 1 | 1 | 1 | 1 | 1 | 1 | 1 | 1 | 1 | 1 | 1 | 0 | 1 | 1 | 1 | 1 | 1 | 1 | 1 | 1 | 19 |
| Ezpeleta et al. (2020a) | 1 | 1 | 1 | 1 | 1 | 1 | 1 | 1 | 1 | 1 | 1 | 0 | 1 | 1 | 1 | 1 | 1 | 1 | 1 | 1 | 19 |
| Ezpeleta et al. (2020b) | 1 | 1 | 1 | 1 | 1 | 1 | 1 | 1 | 1 | 1 | 1 | 0 | 1 | 1 | 1 | 1 | 1 | 0 | 1 | 1 | 18 |
| Ezpeleta et al. (2022) | 1 | 1 | 1 | 1 | 1 | 1 | 1 | 1 | 1 | 1 | 1 | 0 | 1 | 1 | 1 | 1 | 1 | 0 | 1 | 1 | 18 |
| Farchione et al. (2007) | 1 | 1 | 1 | 1 | 1 | 1 | 0 | 1 | 1 | 1 | 1 | 1 | 0 | 0 | 1 | 1 | 1 | 1 | 0 | 1 | 16 |
| Fernandez et al. (2015) | 1 | 1 | 1 | 1 | 1 | 1 | 0 | 1 | 1 | 1 | 1 | 0 | 0 | 0 | 1 | 1 | 1 | 1 | 1 | 0 | 15 |
| Filippi et al. (2020) | 1 | 1 | 1 | 1 | 0 | 0 | 1 | 1 | 1 | 1 | 1 | 1 | 0 | 1 | 1 | 1 | 1 | 1 | 0 | 0 | 15 |
| Gadow & Drabick (2012) | 1 | 1 | 1 | 1 | 1 | 1 | 0 | 1 | 1 | 1 | 1 | 1 | 0 | 0 | 1 | 1 | 1 | 1 | 0 | 1 | 16 |
| Galano et al. (2023) | 1 | 1 | 1 | 1 | 1 | 1 | 1 | 1 | 1 | 1 | 1 | 1 | 1 | 1 | 1 | 1 | 1 | 1 | 0 | 0 | 18 |
| Grabell et al. (2020) | 1 | 1 | 0 | 1 | 1 | 1 | 0 | 1 | 1 | 1 | 1 | 1 | 0 | 0 | 1 | 1 | 1 | 1 | 0 | 0 | 14 |
| Guzick et al. (2021) | 1 | 1 | 1 | 1 | 1 | 1 | 0 | 1 | 1 | 1 | 1 | 0 | 0 | 0 | 1 | 1 | 1 | 1 | 1 | 1 | 16 |
| Harima et al. (2022) | 1 | 1 | 1 | 1 | 1 | 1 | 1 | 1 | 1 | 1 | 1 | 1 | 1 | 1 | 1 | 1 | 1 | 1 | 1 | 1 | 20 |
| Hawes et al. (2020) | 1 | 1 | 1 | 1 | 1 | 1 | 1 | 1 | 1 | 1 | 1 | 1 | 0 | 1 | 1 | 1 | 1 | 1 | 0 | 1 | 18 |
| Kahle et al. (2021) | 1 | 1 | 1 | 1 | 1 | 1 | 1 | 1 | 0 | 1 | 1 | 1 | 1 | 1 | 1 | 1 | 1 | 1 | 1 | 1 | 19 |
| Kalvin et al. (2021) | 1 | 1 | 0 | 1 | 1 | 1 | 0 | 1 | 1 | 1 | 1 | 1 | 0 | 0 | 1 | 1 | 1 | 1 | 1 | 1 | 16 |
| Kessel et al. (2021) | 1 | 1 | 1 | 1 | 1 | 1 | 1 | 1 | 1 | 1 | 1 | 0 | 1 | 0 | 1 | 1 | 1 | 1 | 0 | 1 | 17 |
| Kessel et al. (2017) | 1 | 1 | 1 | 1 | 1 | 1 | 0 | 1 | 1 | 1 | 1 | 0 | 1 | 0 | 1 | 1 | 1 | 1 | 1 | 1 | 17 |
| Kessel et al. (2016) | 1 | 1 | 1 | 1 | 1 | 1 | 0 | 1 | 1 | 1 | 1 | 0 | 1 | 0 | 1 | 1 | 1 | 1 | 0 | 1 | 16 |
| Khurana et al. (2023) | 1 | 1 | 1 | 1 | 1 | 1 | 1 | 1 | 1 | 1 | 1 | 1 | 1 | 0 | 1 | 1 | 1 | 1 | 1 | 1 | 19 |
| Kishida et al. (2022) | 1 | 1 | 1 | 1 | 1 | 1 | 0 | 1 | 1 | 1 | 1 | 1 | 0 | 0 | 1 | 1 | 1 | 1 | 1 | 1 | 17 |
| Kolko et al., (2007) | 1 | 1 | 1 | 1 | 1 | 1 | 1 | 1 | 1 | 1 | 1 | 1 | 1 | 0 | 1 | 1 | 1 | 1 | 0 | 0 | 17 |
| Kolko & Pardini (2010) | 1 | 1 | 1 | 1 | 1 | 1 | 1 | 1 | 1 | 1 | 1 | 1 | 1 | 0 | 1 | 1 | 1 | 1 | 0 | 0 | 17 |
| Krieger et al. (2013) | 1 | 1 | 1 | 1 | 1 | 1 | 1 | 1 | 1 | 1 | 0 | 1 | 0 | 1 | 1 | 1 | 1 | 1 | 1 | 1 | 18 |
| Leadbeater & Ames (2017) | 1 | 1 | 1 | 1 | 1 | 1 | 1 | 1 | 1 | 1 | 1 | 1 | 0 | 1 | 1 | 1 | 1 | 1 | 1 | 1 | 19 |
| Lee et al. (2023) | 1 | 1 | 1 | 1 | 1 | 1 | 1 | 1 | 1 | 1 | 1 | 1 | 1 | 0 | 1 | 1 | 1 | 1 | 1 | 1 | 19 |
| Legenbauer et al. (2018) | 1 | 1 | 1 | 1 | 1 | 1 | 0 | 1 | 1 | 1 | 1 | 1 | 0 | 0 | 1 | 1 | 1 | 1 | 1 | 1 | 17 |
| Leibenluft et al. (2006) | 1 | 1 | 1 | 1 | 1 | 1 | 0 | 1 | 1 | 1 | 1 | 1 | 0 | 0 | 1 | 1 | 1 | 1 | 1 | 1 | 17 |
| Leigh et al. (2020) | 1 | 1 | 1 | 1 | 0 | 0 | 0 | 1 | 1 | 1 | 1 | 1 | 0 | 0 | 1 | 1 | 1 | 1 | 0 | 1 | 14 |
| Lengua (2006) | 1 | 1 | 1 | 1 | 1 | 1 | 1 | 1 | 1 | 1 | 1 | 1 | 0 | 0 | 1 | 1 | 1 | 1 | 0 | 1 | 17 |
| Lengua & Kovacs (2005) | 1 | 1 | 0 | 1 | 1 | 1 | 1 | 1 | 1 | 1 | 1 | 1 | 0 | 0 | 1 | 1 | 1 | 1 | 0 | 1 | 16 |
| Levy et al. (2020) | 1 | 1 | 1 | 1 | 1 | 1 | 0 | 1 | 1 | 1 | 1 | 1 | 0 | 0 | 1 | 1 | 1 | 1 | 0 | 1 | 16 |
| Liu et al. (2024) | 1 | 1 | 1 | 1 | 1 | 1 | 1 | 1 | 1 | 1 | 1 | 1 | 1 | 0 | 1 | 1 | 1 | 1 | 1 | 0 | 18 |
| Loram et al., (2021) | 1 | 1 | 1 | 1 | 1 | 1 | 0 | 1 | 1 | 1 | 1 | 1 | 1 | 0 | 1 | 1 | 1 | 1 | 1 | 1 | 18 |
| Maire et al. (2020) | 1 | 1 | 1 | 1 | 1 | 1 | 0 | 1 | 1 | 1 | 1 | 1 | 0 | 0 | 1 | 1 | 1 | 1 | 0 | 1 | 16 |
| Martin et al. (2016) | 1 | 1 | 1 | 1 | 1 | 1 | 0 | 1 | 1 | 1 | 1 | 1 | 0 | 0 | 1 | 1 | 1 | 1 | 0 | 1 | 16 |
| Mikolajewski et al. (2017) | 1 | 1 | 1 | 1 | 1 | 1 | 1 | 1 | 1 | 1 | 1 | 0 | 0 | 0 | 1 | 1 | 1 | 1 | 1 | 1 | 17 |
| Mulraney et al (2014) | 1 | 1 | 1 | 1 | 0 | 0 | 0 | 1 | 1 | 1 | 0 | 1 | 0 | 0 | 1 | 1 | 1 | 1 | 0 | 0 | 12 |
| Mulraney et al. (2017) | 1 | 1 | 1 | 1 | 1 | 1 | 0 | 1 | 1 | 1 | 1 | 0 | 0 | 0 | 1 | 1 | 1 | 1 | 0 | 0 | 14 |
| Naim et al. (2021) | 1 | 1 | 1 | 1 | 1 | 1 | 0 | 1 | 1 | 1 | 1 | 1 | 0 | 0 | 1 | 1 | 1 | 1 | 0 | 1 | 16 |
| Nelson et al. (2018) | 1 | 1 | 1 | 1 | 1 | 1 | 1 | 1 | 1 | 1 | 1 | 0 | 0 | 1 | 1 | 1 | 1 | 1 | 1 | 0 | 17 |
| Pan & Yeh (2019) | 1 | 1 | 1 | 1 | 1 | 1 | 0 | 1 | 1 | 0 | 1 | 1 | 0 | 0 | 1 | 1 | 1 | 1 | 0 | 1 | 15 |
| Perhamus & Ostrov (2021) | 1 | 1 | 1 | 1 | 1 | 1 | 1 | 1 | 1 | 1 | 1 | 0 | 0 | 1 | 1 | 1 | 1 | 1 | 1 | 1 | 18 |
| Poznanski et al. (2018) | 1 | 1 | 1 | 1 | 1 | 1 | 0 | 1 | 1 | 1 | 1 | 0 | 0 | 1 | 1 | 1 | 1 | 1 | 1 | 1 | 17 |
| Rappaport et al. (2020) | 1 | 1 | 1 | 1 | 1 | 1 | 0 | 1 | 1 | 0 | 1 | 0 | 0 | 0 | 0 | 1 | 1 | 1 | 1 | 1 | 14 |
| Rice et al. (2017) | 1 | 1 | 1 | 1 | 1 | 1 | 0 | 1 | 1 | 1 | 0 | 0 | 0 | 0 | 1 | 1 | 1 | 1 | 1 | 1 | 15 |
| Rowe et al. (2010) | 1 | 1 | 1 | 1 | 1 | 1 | 0 | 1 | 1 | 1 | 1 | 1 | 0 | 0 | 1 | 1 | 1 | 1 | 0 | 1 | 16 |
| Rubens et al. (2017) | 1 | 1 | 1 | 1 | 1 | 1 | 0 | 1 | 1 | 1 | 1 | 0 | 0 | 0 | 1 | 1 | 1 | 1 | 0 | 1 | 15 |
| Silver et al. (2021) | 1 | 1 | 1 | 1 | 1 | 1 | 0 | 1 | 1 | 1 | 1 | 1 | 0 | 0 | 1 | 1 | 1 | 1 | 0 | 1 | 16 |
| Silver et al. (2024) | 1 | 1 | 1 | 1 | 1 | 1 | 1 | 1 | 1 | 1 | 1 | 1 | 1 | 1 | 1 | 1 | 1 | 1 | 1 | 1 | 20 |
| Smith et al. (2019) | 1 | 1 | 1 | 1 | 1 | 1 | 0 | 1 | 1 | 1 | 1 | 1 | 0 | 0 | 1 | 1 | 1 | 0 | 0 | 0 | 14 |
| Sorcher et al. (2022) | 1 | 1 | 1 | 1 | 1 | 1 | 0 | 1 | 1 | 1 | 1 | 1 | 0 | 0 | 1 | 1 | 1 | 1 | 1 | 0 | 16 |
| Srinivasan et al. (2024) | 1 | 1 | 1 | 1 | 1 | 1 | 0 | 1 | 1 | 1 | 1 | 1 | 1 | 0 | 1 | 1 | 1 | 1 | 1 | 1 | 18 |
| Stoddard et al. (2017) | 1 | 1 | 1 | 1 | 1 | 1 | 0 | 1 | 1 | 0 | 1 | 1 | 0 | 0 | 1 | 1 | 1 | 1 | 1 | 1 | 16 |
| Stringaris & Goodman (2009a) | 1 | 1 | 1 | 1 | 1 | 1 | 0 | 1 | 1 | 1 | 1 | 1 | 1 | 0 | 1 | 1 | 1 | 0 | 0 | 1 | 16 |
| Stringaris & Goodman (2009b) | 1 | 1 | 1 | 1 | 1 | 1 | 0 | 1 | 1 | 1 | 1 | 1 | 1 | 0 | 1 | 1 | 1 | 1 | 1 | 1 | 18 |
| Stringaris et al. (2012) | 1 | 1 | 1 | 1 | 1 | 1 | 1 | 1 | 1 | 0 | 1 | 1 | 1 | 1 | 1 | 1 | 1 | 1 | 0 | 1 | 18 |
| Theriault et al. (2018) | 1 | 1 | 0 | 1 | 1 | 1 | 1 | 1 | 1 | 1 | 1 | 1 | 1 | 1 | 1 | 1 | 1 | 1 | 0 | 1 | 18 |
| Ucar & Vural (2018) | 1 | 1 | 1 | 1 | 1 | 1 | 0 | 1 | 1 | 1 | 1 | 1 | 0 | 0 | 1 | 1 | 1 | 1 | 0 | 1 | 16 |
| Valencia et al. (2021) | 1 | 1 | 1 | 1 | 1 | 1 | 1 | 1 | 1 | 1 | 1 | 0 | 0 | 1 | 1 | 1 | 1 | 1 | 0 | 1 | 17 |
| Vogel et al. (2019) | 1 | 1 | 1 | 1 | 1 | 1 | 0 | 1 | 1 | 1 | 1 | 0 | 0 | 0 | 1 | 1 | 1 | 0 | 1 | 0 | 14 |
| Wakschlag et al. (2015) | 1 | 1 | 1 | 1 | 1 | 1 | 1 | 1 | 1 | 1 | 1 | 1 | 0 | 1 | 1 | 1 | 1 | 1 | 1 | 1 | 19 |
| Wakschlag et al. (2020) | 1 | 1 | 1 | 1 | 1 | 1 | 1 | 1 | 1 | 1 | 1 | 1 | 0 | 0 | 1 | 1 | 1 | 0 | 0 | 0 | 15 |
| Wang et al. (2023) | 1 | 1 | 1 | 1 | 1 | 1 | 0 | 1 | 1 | 1 | 1 | 1 | 1 | 0 | 1 | 1 | 1 | 1 | 1 | 1 | 18 |
| Waschbusch et al. (2020) | 1 | 1 | 1 | 1 | 0 | 0 | 0 | 1 | 1 | 1 | 1 | 1 | 0 | 0 | 1 | 1 | 1 | 1 | 0 | 1 | 14 |
| Waxmonsky et al. (2022) | 1 | 1 | 1 | 1 | 1 | 1 | 0 | 1 | 1 | 1 | 1 | 1 | 0 | 0 | 1 | 1 | 1 | 1 | 1 | 1 | 17 |
| Waxmonsky et al. (2017) | 1 | 1 | 1 | 1 | 1 | 1 | 0 | 1 | 1 | 1 | 1 | 1 | 0 | 0 | 1 | 1 | 1 | 1 | 1 | 1 | 17 |
| Whelan et al. (2015) | 1 | 1 | 1 | 1 | 1 | 1 | 1 | 1 | 1 | 1 | 1 | 1 | 0 | 1 | 1 | 1 | 1 | 1 | 1 | 1 | 19 |
| Wiggins et al. (2023) | 1 | 1 | 1 | 1 | 1 | 1 | 0 | 1 | 1 | 1 | 1 | 0 | 1 | 0 | 1 | 1 | 1 | 1 | 1 | 0 | 16 |
| Wilson et al. (2022) | 1 | 1 | 1 | 1 | 1 | 1 | 0 | 1 | 1 | 1 | 1 | 1 | 0 | 0 | 1 | 1 | 1 | 1 | 1 | 1 | 17 |
| Zendarskiet al. (2023) | 1 | 1 | 1 | 1 | 1 | 1 | 0 | 1 | 1 | 1 | 1 | 1 | 1 | 0 | 1 | 1 | 1 | 1 | 1 | 1 | 18 |
| Zhou et al. (2009) | 1 | 1 | 1 | 1 | 1 | 1 | 1 | 1 | 1 | 1 | 1 | 1 | 0 | 1 | 1 | 1 | 1 | 1 | 0 | 1 | 18 |
| Zik et al. (2022) | 1 | 1 | 1 | 1 | 1 | 1 | 0 | 1 | 1 | 0 | 1 | 1 | 0 | 0 | 1 | 1 | 1 | 1 | 1 | 1 | 16 |

**Table S3**

*Excluded Studies at Full-Text Review with Reasons for Exclusion*

| **First Author** | **Year Published** | **Reason for exclusion** | **Reference** |
| --- | --- | --- | --- |
| Aebi | 2010 | Mental health outcomes not measured | Aebi, M., Müller, U. C., Asherson, P., Banaschewski, T., Buitelaar, J., Ebstein, R., Eisenberg, J., Gill, M., Manor, I., Miranda, A., Oades, R. D., Roeyers, H., Rothenberger, A., Sergeant, J., Sonuga-Barke, E. J. S., Thompson, M., Taylor, E., Faraone, S. V., & Steinhausen, H. C. (2010). Predictability of oppositional defiant disorder and symptom dimensions in children and adolescents with ADHD combined type. *Psychological Medicine*, *40*(12), 2089-2100. doi:10.1017/s0033291710000590 |
| Aebi | 2016 | Mental health outcomes not measured | Aebi, M., Van Donkelaar, M. M., Poelmans, G., Buitelaar, J. K., Sonuga‐Barke, E. J., Stringaris, A., Consortium, I., Faraone, S. V., Franke, B., Steinhausen, H. C., & van Hulzen, K. J. (2016). Gene‐set and multivariate genome‐wide association analysis of oppositional defiant behavior subtypes in attention‐deficit/hyperactivity disorder. *American Journal of Medical Genetics Part B, Neuropsychiatric Genetics*, *171B*(5), 573-588. doi:10.1002/ajmg.b.32346 |
| Anderson | 2011 | Irritability not appropriately measured | Anderson, D. K., Maye, M. P., & Lord, C. (2011). Changes in maladaptive behaviors from mid-childhood to young adulthood in autism spectrum disorder. *American Journal on Intellectual and Developmental Disabilities*, *116*(5), 381-397. doi:10.1352/1944-7558-116.5.381 |
| Bajaj | 2021 | Mental health outcomes not measured | Bajaj, S., Blair, K. S., Bashford-Largo, J., Zhang, R., Mathur, A., Schwartz, A., Elowsky, J., Dobbertin, M., Hwang, S., Leibenluft, E., & Blair, R. J. R. (2021). Network-wise surface-based morphometric insight into the cortical neural circuitry underlying irritability in adolescents. *Translational Psychiatry, 11*(1), 581. doi:10.1038/s41398-021-01710-2 |
| Baji | 2012 | Irritability not appropriately measured | Baji, I., Gádoros, J., Kiss, E., Mayer, L., Kovács, E., Benák, I., & Vetró, A. (2012). Symptoms of depression in children and adolescents in relation to psychiatric comorbidities. *Neuropsychiatrie de l'Enfance et de l'Adolescence, 60*(5), S167. doi:10.1016/j.neurenf.2012.04.240 |
| Baweja | 2021 | Mental health outcomes not measured | Baweja, R., Waxmonsky, J. G., Babinski, D., Fosco, W., & Waschbusch, D. (2021). Measurement of irritability: role of cross-informants and correlation with ODD symptoms. *Journal of the American Academy of Child & Adolescent Psychiatry, 60*(10), S199. doi:10.1016/j.jaac.2021.09.210 |
| Becht | 2016 | Irritability not appropriately measured | Becht, A. I., Prinzie, P., Deković, M., Van den Akker, A. L., & Shiner, R. L. (2016). Child personality facets and overreactive parenting as predictors of aggression and rule-breaking trajectories from childhood to adolescence. *Development and Psychopathology*, *28*(2), 399-413. doi:10.1017/s0954579415000577 |
| Bertocci | 2019 | Irritability not appropriately measured | Bertocci, M. A., Hanford, L., Manelis, A., Iyengar, S., Youngstrom, E. A., Gill, M. K., Monk, K., Versace, A., Bonar, L., Bebko, G., Ladouceur, C. D., Perlman, S. B., Diler, R., Horwitz, S. M., Arnold, L. E., Hafeman, D., Travis, M. J., Kowatch, R., Holland, S. K., Fristad, M. A., Findling, R. L., Birmaher, B., & Phillips, M. L. (2019). Clinical, cortical thickness and neural activity predictors of future affective lability in youth at risk for bipolar disorder: initial discovery and independent sample replication. *Molecular Psychiatry*, *24*(12), 1856-1867. doi:10.1038/s41380-018-0273-4 |
| Bezek | 2021 | Mental health outcomes not measured | Bezek, J., Cardinale, E. M., Morales, S., Filippi, C., Smith, A. R., Haller, S., Valadez, E., Harrewijn, A., Phillips, D., Chronis-Tuscano, A., Fox, N., Pine, D., Leibenluft, E., & Kircanski, K. (2021). Mapping anxiety and irritability trajectories over time: associations with brain response during cognitive conflict. *Biological Psychiatry*, *89*(9), S203-S204. doi:10.1016/j.biopsych.2021.02.516 |
| Birmaher | 2009 | Irritability not appropriately measured | Birmaher, B., Axelson, D., Strober, M., Gill, M. K., Yang, M., Ryan, N., Goldstein, B., Hunt, J., Esposito-Smythers, C., Iyengar, S., Goldstein, T., Chiapetta, L., Keller, M., & Leonard, H. (2009). Comparison of manic and depressive symptoms between children and adolescents with bipolar spectrum disorders. *Bipolar Disorders, 11*(1), 52-62. doi:10.1111/j.1399-5618.2008.00659.x |
| Birmaher | 2013 | Irritability not appropriately measured | Birmaher, B., Goldstein, B. I., Axelson, D. A., Monk, K., Hickey, M. B., Fan, J., Iyengar, S., Ha, W., Diler, R. S., Goldstein, T., Brent, D., Ladouceur, C. D., Sakolsky, D., & Kupfer, D. J. (2013). Mood lability among offspring of parents with bipolar disorder and community controls. *Bipolar Disorders, 15*(3), 253-263. doi:10.1111/bdi.12060 |
| Blader | 2016 | Irritability not appropriately measured | Blader, J. C., Pliszka, S. R., Kafantaris, V., Sauder, C., Posner, J., Foley, C. A., Carlson, G. A., Crowell, J. A., & Margulies, D. M. (2016). Prevalence and treatment outcomes of persistent negative mood among children with attention-deficit/hyperactivity disorder and aggressive behavior. *Journal of Child and Adolescent Psychopharmacology, 26*(2), 164-173. doi:10.1089/cap.2015.0112 |
| Blair | 2020 | Data unavailable | Blair, R. J. R., Bashford-Largo, J., Zhang, R., Lukoff, J., Elowsky, J. S., Leibenluft, E., Hwang, S., Dobbertin, M., & Blair, K. S. (2020). Temporal discounting impulsivity and its association with conduct disorder and irritability. *Journal of Child and Adolescent Psychopharmacology, 30*(9), 542-548. doi:10.1089/cap.2020.0001 |
| Blanken | 2021 | Irritability not appropriately measured | Blanken, T. F., Courbet, O., Franc, N., Albajara Sáenz, A., Van Someren, E. J., Peigneux, P., & Villemonteix, T. (2021). Is an irritable ADHD profile traceable using personality dimensions? Replicability, stability, and predictive value over time of data-driven profiles. *European Child & Adolescent Psychiatry, 30*, 633-645. doi:10.1007/s00787-020-01546-z |
| Bohaterewicz | 2020 | Irritability not appropriately measured | Bohaterewicz, B., Nowicka, M., Sobczak, A. M., Plewka, A. A., Gaszczyk, P., & Marek, T. (2020). Clinical and psychosocial characteristics of adolescent pediatric patients hospitalized after different types of suicidal behaviors—A preliminary study. *International Journal of Environmental Research and Public Health*, *17*(15), 5568. doi:10.3390/ijerph17155568 |
| Bufferd | 2017 | Irritability not appropriately measured | Bufferd, S. J., Dougherty, L. R., & Olino, T. M. (2017). Mapping the frequency and severity of depressive behaviors in preschool-aged children. *Child Psychiatry & Human Development*, *48*, 934-943. doi:10.1007/s10578-017-0715-2 |
| Burk | 2011 | Irritability not appropriately measured | Burk, L. R., Armstrong, J. M., Park, J.-H., Zahn-Waxler, C., Klein, M. H., & Essex, M. J. (2011). Stability of early identified aggressive victim status in elementary school and associations with later mental health problems and functional impairments. *Journal of Abnormal Child Psychology, 39*(2), 225-238. doi:10.1007/s10802-010-9454-6 |
| Burke | 2010 | Data duplicated or not suitable | Burke, J. D., Hipwell, A. E., & Loeber, R. (2010). Dimensions of oppositional defiant disorder as predictors of depression and conduct disorder in preadolescent girls. *Journal of the American Academy of Child and Adolescent Psychiatry, 49*(5), 484–492. doi:10.1016/j.jaac.2010.01.016 |
| Carlson | 2016 | Data unavailable | Carlson, G. A., Danzig, A. P., Dougherty, L. R., Bufferd, S. J., & Klein, D. N. (2016). Loss of temper and irritability: the relationship to tantrums in a community and clinical sample. *Journal of Child and Adolescent Psychopharmacology, 26*(2), 114-122. doi:10.1089/cap.2015.0072 |
| Carpenter | 2019 | Data unavailable | Carpenter, K. L. H., Baranek, G. T., Copeland, W. E., Compton, S., Zucker, N., Dawson, G., & Egger, H. L. (2019). Sensory over-responsivity: An early risk factor for anxiety and behavioral challenges in young children. *Journal of Abnormal Child Psychology, 47*(6), 1075-1088. doi:10.1007/s10802-018-0502-y |
| Cavanagh | 2017 | Irritability not appropriately measured | Cavanagh, M., Quinn, D., Duncan, D., Graham, T., & Balbuena, L. (2017). Oppositional defiant disorder is better conceptualized as a disorder of emotional regulation. *Journal of Attention Disorders*, *21*(5), 381-389. doi:10.1177/1087054713520221 |
| Chaarani | 2020 | Data unavailable | Chaarani, B., Kan, K.-J., Mackey, S., Potter, A., Banaschewski, T., Millenet, S., Bokde, A. L. W., Bromberg, U., Büchel, C., Conrod, P. J., Flor, H., Gowland, P., Heinz, A., Martinot, J.-L., Nees, F., Walter, H., Whelan, R., Higgins, S. T., Schumann, G., . . . Stedman, A. (2020). Neural correlates of adolescent irritability and its comorbidity with psychiatric disorders. *Journal of the American Academy of Child and Adolescent Psychiatry, 59*(12), 1371-1379. doi:10.1016/j.jaac.2019.11.028 |
| Chacón-Cuberos | 2018 | Irritability not appropriately measured | Chacón-Cuberos, R., Castro-Sánchez, M., González-Campos, G., & Zurita-Ortega, F. (2018). Victimization in school, digital leisure and irritability: Analysis using structural equations. *Revista Electrónica de Investigación y Evaluación Educativa*, *24*(3). doi:10.7203/relieve.24.1.12614 |
| Chan | 2011 | Irritability not appropriately measured | Chan, J., Stringaris, A., & Ford, T. (2011). Bipolar disorder in children and adolescents recognised in the UK: A clinic‐based study. *Child and Adolescent Mental Health*, *16*(2), 71-78. doi:10.1111/j.1475-3588.2010.00566.x |
| Colpan | 2018 | Mental health outcomes not measured | Çolpan, M., Eray, Ş., Eren, E., & Vural, A. P. (2018). Perceived expressed emotion, emotional and behavioral problems and self-esteem in obese adolescents: a case-control study. *Journal of Clinical Research in Pediatric Endocrinology*, *10*(4), 357. doi:10.4274/jcrpe.0101 |
| Comer | 2012 | Irritability not appropriately measured | Comer, J. S., Pincus, D. B., & Hofmann, S. G. (2012). Generalized anxiety disorder and the proposed associated symptoms criterion change for DSM‐5 in a treatment‐seeking sample of anxious youth. *Depression and Anxiety*, *29*(12), 994-1003. doi:10.1002/da.21999 |
| Cooke | 2009 | Irritability not appropriately measured | Cooke, L. S., & Jones, S. H. (2009). An evaluation of cognitions, mood and behaviours in late adolescents: A study of associations with risk for bipolar disorder. *Personality and Individual Differences, 46*(3), 314-318. doi:10.1016/j.paid.2008.10.021 |
| Cordell | 2015 | Irritability not appropriately measured | Cordell, K., & Snowden, L. (2015). Emotional distress dispositions and crisis intervention for children treated for mental illness. *Journal of Child and Family Studies*, *24*, 2699-2709. doi:10.1007/s10826-014-0072-8 |
| Cornacchio | 2016 | Data unavailable | Cornacchio, D., Crum, K. I., Coxe, S., Pincus, D. B., & Comer, J. S. (2016). Irritability and severity of anxious symptomatology among youth with anxiety disorders. *Journal of the American Academy of Child & Adolescent Psychiatry, 55*(1), 54-61. doi:10.1016/j.jaac.2015.10.007 |
| Crowe | 2006 | Irritability not appropriately measured | Crowe, M., Ward, N., Dunnachie, B., & Roberts, M. (2006). Characteristics of adolescent depression. *International Journal of Mental Health Nursing*, *15*(1), 10-18. doi:10.1111/j.1447-0349.2006.00399.x |
| Crum | 2021 | Mental health outcomes not measured | Crum, K. I., Hwang, S., Blair, K. S., Aloi, J. M., Meffert, H., White, S. F., Tyler, P. M., Leibenluft, E., Pope, K., & Blair, R. J. R. (2021). Interaction of irritability and anxiety on emotional responding and emotion regulation: a functional MRI study. *Psychological Medicine*, *51*(16), 2778-2788. doi:10.1017/s0033291720001397 |
| Damme | 2022 | Data duplicated or not suitable | Damme, K. S. F., Norton, E. S., Briggs-Gowan, M. J., Wakschlag, L. S., & Mittal, V. A. (2022). Developmental Patterning of Irritability Enhances Prediction of Psychopathology in Preadolescence: Improving RDoC With Developmental Science. Journal of Psychopathology and Clinical Science, 131(6), 556–566. doi:10.1037/abn0000655 |
| Dennis | 2019 | Mental health outcomes not measured | Dennis, E. L., Humphreys, K. L., King, L. S., Thompson, P. M., & Gotlib, I. H. (2019). Irritability and brain volume in adolescents: cross-sectional and longitudinal associations. *Social Cognitive and Affective Neuroscience, 14*(7), 687-698. doi:10.1093/scan/nsz053 |
| Derella | 2020 | Data unavailable | Derella, O. J., Burke, J. D., Stepp, S. D., & Hipwell, A. E. (2020). Reciprocity in undesirable parent-child behavior? Verbal aggression, corporal punishment, and girls' oppositional defiant symptoms. *Journal of Clinical Child and Adolescent Psychology, 49*(3), 420-433. doi:10.1080/15374416.2019.1603109 |
| Derella | 2019 | Mental health outcomes not measured | Derella, O. J., Johnston, O. G., Loeber, R., & Burke, J. D. (2019). CBT-Enhanced emotion regulation as a mechanism of improvement for childhood irritability. *Journal of Clinical Child and Adolescent Psychology, 48*(sup1), S146-S154. doi:1080/15374416.2016.1270832 |
| De Pauw | 2009 | Irritability not appropriately measured | De Pauw, S. S. W., Mervielde, I., & Van Leeuwen, K. G. (2009). How are traits related to problem behavior in preschoolers? similarities and contrasts between temperament and personality. *Journal of Abnormal Child Psychology, 37*(3), 309-325. doi:10.1007/s10802-008-9290-0 |
| Derella | 2024 | Data unavailable | Derella, O. J., Butler, E. J., Seymour, K. E., & Burke, J. D. (2024). Frustration Response and Regulation Among Irritable Children: Contributions of Chronic Irritability, Internalizing, and Externalizing Symptoms. Journal of Clinical Child and Adolescent Psychology, 53(2), 199–215. doi:10.1080/15374416.2023.2246557 |
| Deveney | 2017 | Mental health outcomes not measured | Deveney, C., Pagliaccio, D., Estabrook, R., Burns, J., Voss, J., Zobel, E., Brotman, M., Briggs-Gowan, M. & Wakschlag, L. (2017). Temporally sensitive neural measures of inhibition in preschool children with varying irritability symptoms. *Biological Psychiatry, 81*(10), S17. doi:10.1016/j.biopsych.2017.02.050 |
| Deveney | 2019 | Mental health outcomes not measured | Deveney, C. M., Briggs‐Gowan, M. J., Pagliaccio, D., Estabrook, C. R., Zobel, E., Burns, J. L., Norton, E. S., Pine, D. S., Brotman, M. A., Leibenluft, E., & Wakschlag, L. S. (2019). Temporally sensitive neural measures of inhibition in preschool children across a spectrum of irritability. *Developmental Psychobiology*, *61*(2), 216-227. doi:10.1002/dev.21792 |
| Deveney | 2013 | Irritability not appropriately measured | Deveney, C. M., Connolly, M. E., Haring, C. T., Bones, B. L., Reynolds, R. C., Kim, P., Pine, D. S. & Leibenluft, E. (2013). Neural mechanisms of frustration in chronically irritable children. *American Journal of Psychiatry, 170*(10), 1186-1194. doi:10.1176/appi.ajp.2013.12070917 |
| Deveney | 2020 | Mental health outcomes not measured | Deveney, C. M., Grasso, D., Hsu, A., Pine, D. S., Estabrook, C. R., Zobel, E., Burns, J. L., Wakschlag, L. S., & Briggs-Gowan, M. J. (2020). Multi‐method assessment of irritability and differential linkages to neurophysiological indicators of attention allocation to emotional faces in young children. *Developmental Psychobiology*, *62*(5), 600-616. doi:10.1002/dev.21930 |
| Diaz-Stransky | 2020 | Data unavailable | Diaz-Stransky, A., Rowley, S., Zecher, E., Grodberg, D., & Sukhodolsky, D. G. (2020). Tantrum tool: development and open pilot study of online parent training for irritability and disruptive behavior. *Journal of Child and Adolescent Psychopharmacology, 30*(9), 558-566. doi:10.1089/cap.2020.0089 |
| Dickstein | 2016 | Non-empirical article | Dickstein, D. P. (2016). Mechanisms distinguishing irritability in children and adolescents. *The American Journal of Psychiatry, 173*(7), 653-654. doi:10.1176/appi.ajp.2016.16040429 |
| Diler | 2007 | Irritability not appropriately measured | Diler, R. S., Daviss, W. B., Lopez, A., Axelson, D., Iyengar, S., & Birmaher, B. (2007). Differentiating major depressive disorder in youths with attention deficit hyperactivity disorder. *Journal of Affective Disorders, 102*(1-3), 125-130. doi:10.1016/j.jad.2007.01.002 |
| Dissanayake | 2024 | Data duplicated or not suitable | Dissanayake, A. S., Dupuis, A., Arnold, P. D., Burton, C. L., Crosbie, J., Schachar, R. J., & Levy, T. (2024). Is irritability multidimensional: Psychometrics of The Irritability and Dysregulation of Emotion Scale (TIDES-13). European Child & Adolescent Psychiatry. doi:10.1007/s00787-023-02350-1 |
| Dönmez | 2021 | Irritability not appropriately measured | Dönmez, Y. E., & Uçur, Ö. (2021). Frequency of anxiety, depression, and irritability symptoms in children during the COVID-19 outbreak and potential risk factors associated with these symptoms. *The Journal of Nervous and Mental Disease, 209*(10), 727-733. doi:10.1097/nmd.0000000000001364 |
| Dougherty | 2017 | Data duplicated or not suitable | Dougherty, L. R., Barrios, C. S., Carlson, G. A., & Klein, D. N. (2017). Predictors of later psychopathology in young children with disruptive mood dysregulation disorder. *Journal of Child and Adolescent Psychopharmacology, 27*(5), 396–402. doi:10.1089/cap.2016.0144 |
| Dougherty | 2018 | Mental health outcomes not measured | Dougherty, L. R., Schwartz, K. T., Kryza-Lacombe, M., Weisberg, J., Spechler, P. A., & Wiggins, J. L. (2018). Preschool-and school-age irritability predict reward-related brain function. *Journal of the American Academy of Child & Adolescent Psychiatry, 57*(6), 407-417. doi:10.1016/j.jaac.2018.03.012 |
| Dugré | 2022 | Data duplicated or not suitable | Dugré, J. R., & Potvin, S. (2022). Multiple developmental pathways underlying conduct problems: A multitrajectory framework. Development and Psychopathology, 34(3), 1115–1124. doi:10.1017/S0954579420001650 |
| Dyce | 2020 | Irritability not appropriately measured | Dyce, L., Sassi, R. B., & Boylan, K. (2020). Examining the predictive association of irritability with borderline personality disorder in a clinical sample of female adolescents. *Personality and Mental Health, 14*(2), 167-174. doi:10.1002/pmh.1469 |
| Eisenberg | 2001 | Irritability not appropriately measured | Eisenberg, N., Cumberland, A., Spinrad, T. L., Fabes, R. A., Shepard, S. A., Reiser, M., Murphy, B. C., Losoya, S. H., & Guthrie, I. K. (2001). The relations of regulation and emotionality to children's externalizing and internalizing problem behavior. *Child Development, 72*(4), 1112-1134. doi:10.1111/1467-8624.00337 |
| Estes | 2007 | Irritability not appropriately measured | Estes, A. M., Dawson, G., Sterling, L., & Munson, J. (2007). Level of intellectual functioning predicts patterns of associated symptoms in school-age children with autism spectrum disorder. *American Journal on Mental Retardation, 112*(6), 439-449. doi:10.1352/0895-8017(2007)112[439:Loifpp]2.0.Co;2 |
| Euler | 2015 | Irritability not appropriately measured | Euler, F., Jenkel, N., Stadler, C., Schmeck, K., Fegert, J. M., Kölch, M., & Schmid, M. (2015). Variants of girls and boys with conduct disorder: Anxiety symptoms and callous-unemotional traits. *Journal of Abnormal Child Psychology, 43*, 773-785. doi:10.1007/s10802-014-9946-x |
| Evans | 2020 | Data unavailable | Evans, S. C., Weisz, J. R., Carvalho, A. C., Garibaldi, P. M., Bearman, S. K., & Chorpita, B. F. (2020). Effects of standard and modular psychotherapies in the treatment of youth with severe irritability. *Journal of Consulting and Clinical Psychology, 88*(3), 255. doi:10.1037/ccp0000456 |
| Eyre | 2019 | Data duplicated or not suitable | Eyre, O., Riglin, L., Leibenluft, E., Stringaris, A., Collishaw, S., & Thapar, A. (2019). Irritability in ADHD: association with later depression symptoms. *European Child & Adolescent Psychiatry, 28*(10), 1375-1384. doi:10.1007/s00787-019-01303-x |
| Ezpeleta | 2016 | Data unavailable | Ezpeleta, L., Granero, R., de la Osa, N., Trepat, E., & Domenech, J. M. (2016). Trajectories of oppositional defiant disorder irritability symptoms in preschool children. *Journal of Abnormal Child Psychology, 44*(1), 115-128. doi:10.1007/s10802-015-9972-3 |
| Ezpeleta | 2022 | Mental health outcomes not measured | Ezpeleta, L., Penelo, E., Navarro, J. B., de la Osa, N., Trepat, E., & Wichstrøm, L. (2022). Reciprocal relations between dimensions of Oppositional defiant problems and callous-unemotional traits. *Research on Child and Adolescent Psychopathology, 50*(9), 1179-1190. doi:10.1007/s10802-022-00910-8 |
| Fergus | 2003 | Irritability not appropriately measured | Fergus, E. L., Miller, R. B., Luckenbaugh, D. A., Leverich, G. S., Findling, R. L., Speer, A. M., & Post, R. M. (2003). Is there progression from irritability/dyscontrol to major depressive and manic symptoms? A retrospective community survey of parents of bipolar children. *Journal of Affective Disorders, 77*(1), 71-78. doi:10.1016/s0165-0327(02)00176-3 |
| Fichter | 2009 | Irritability not appropriately measured | Fichter, M. M., Kohlboeck, G., Quadflieg, N., Wyschkon, A., & Esser, G. (2009). From childhood to adult age: 18-year longitudinal results and prediction of the course of mental disorders in the community. *Social Psychiatry and Psychiatric Epidemiology, 44*, 792-803. doi:10.1007/s00127-009-0501-y |
| Fidan | 2011 | Irritability not appropriately measured | Fidan, T. (2011). Assessment of mental symptoms and risk factors in children and adolescents who admitted to the child-adolescent psychiatry clinic. *Konuralp Medical Journal*, 3(1), 1-8. |
| Fishburn | 2019 | Mental health outcomes not measured | Fishburn, F. A., Hlutkowsky, C. O., Bemis, L. M., Huppert, T. J., Wakschlag, L. S., & Perlman, S. B. (2019). Irritability uniquely predicts prefrontal cortex activation during preschool inhibitory control among all temperament domains: A LASSO approach. *Neuroimage, 184*, 68-77. doi:10.1016/j.neuroimage.2018.09.023 |
| Fongaro | 2022 | Data duplicated or not suitable | Fongaro, E., Picot, M. C., Stringaris, A., Belloc, C., Verissimo, A. S., Franc, N., & Purper-Ouakil, D. (2022). Parent training for the treatment of irritability in children and adolescents: a multisite randomized controlled, 3-parallel-group, evaluator-blinded, superiority trial. BMC Psychology, 10(1), 1–273. doi:10.1186/s40359-022-00984-5 |
| Forte | 2021 | Sample outside age range | Forte, A., Orri, M., Turecki, G., Galera, C., Pompili, M., Boivin, M., Tremblay, R. E., Cote, S. M., & Geoffroy, M. C. (2021). Identifying environmental pathways between irritability during childhood and suicidal ideation and attempt in adolescence: Findings from a 20‐year population‐based study. *Journal of Child Psychology and Psychiatry, 62*(12), 1402-1411. doi:10.1111/jcpp.13411 |
| Fowler | 2013 | Irritability not appropriately measured | Fowler, S., & Szabó, M. (2013). The emotional experience associated with worrying in adolescents. *Journal of Psychopathology and Behavioral Assessment, 35*(1), 65-75. doi:10.1007/s10862-012-9316-3 |
| Fraser | 2018 | Irritability not appropriately measured | Fraser, A., Cooper, M., Agha, S. S., Collishaw, S., Rice, F., Thapar, A., & Eyre, O. (2018). The presentation of depression symptoms in attention‐deficit/hyperactivity disorder: comparing child and parent reports. *Child and Adolescent Mental Health, 23*(3), 243-250. doi:10.1111/camh.12253 |
| Frazier | 2016 | Irritability not appropriately measured | Frazier, E. A., Liu, R. T., Massing-Schaffer, M., Hunt, J., Wolff, J., & Spirito, A. (2016). Adolescent but not parent report of irritability is related to suicidal ideation in psychiatrically hospitalized adolescents. *Archives of Suicide Research, 20*(2), 280-289. doi:10.1080/13811118.2015.1004497 |
| Fristad | 2016 | Mental health outcomes not measured | Fristad, M. A., Wolfson, H., Algorta, G. P., Youngstrom, E. A., Arnold, L. E., Birmaher, B., Horwitz, S., Axelson, D., Kowatch, R. A., Findling, R. L., & Grp, L. (2016). Disruptive mood dysregulation disorder and bipolar disorder not otherwise specified: fraternal or identical twins? *Journal of Child and Adolescent Psychopharmacology, 26*(2), 138-146. doi:10.1089/cap.2015.0062 |
| Froehlich | 2020 | Irritability not appropriately measured | Froehlich, T. E., Brinkman, W. B., Peugh, J. L., Piedra, A. N., Vitucci, D. J., & Epstein, J. N. (2020). Pre-existing comorbid emotional symptoms moderate short-term methylphenidate adverse effects in a randomized trial of children with attention-deficit/hyperactivity disorder. *Journal of Child and Adolescent Psychopharmacology, 30*(3), 137-147. doi:10.1089/cap.2019.0125 |
| Gabbay | 2015 | Irritability not appropriately measured | Gabbay, V., Johnson, A. R., Alonso, C. M., Evans, L. K., Babb, J. S., & Klein, R. G. (2015). Anhedonia, but not irritability, is associated with illness severity outcomes in adolescent major depression. *Journal of Child and Adolescent Psychopharmacology, 25(3), 194-200.*  doi:10.1089/cap.2014.0105 |
| Gadow | 2012 | Irritability not appropriately measured | Gadow, K. D., & Drabick, D. A. (2012). Symptoms of autism and schizophrenia spectrum disorders in clinically referred youth with oppositional defiant disorder. *Research in Developmental Disabilities, 33*(4), 1157-1168. doi:10.1016/j.ridd.2012.01.004 |
| Galera | 2021 | Data duplicated or not suitable | Galera, C., Orri, M., Vergunst, F., Melchior, M., Van der Waerden, J., Bouvard, M. P., Collet, O., Boivin, M., Tremblay, R. E., & Cote, S. M. (2021). Developmental profiles of childhood attention-deficit/hyperactivity disorder and irritability: association with adolescent mental health, functional impairment, and suicidal outcomes. *Journal of Child Psychology and Psychiatry, 62*(2), 232-243. doi.org/10.1111/jcpp.13270 |
| Ghanizadeh | 2006 | Irritability not appropriately measured | Ghanizadeh, A., Khajavian, S., & Ashkani, H. (2006). Prevalence of psychiatric disorders, depression, and suicidal behavior in child and adolescent with thalassemia major. *Journal of Pediatric Hematology/Oncology, 28*(12), 781-784. doi:10.1097/01.mph.0000243665.79303.9e |
| Giller | 2022 | Mental health outcomes not measured | Giller, F., Aggensteiner, P. M., Banaschewski, T., Döpfner, M., Brandeis, D., Roessner, V., & Beste, C. (2022). Affective dysregulation in children is associated with difficulties in response control in emotional ambiguous situations. *Biological Psychiatry: Cognitive Neuroscience and Neuroimaging, 7*(1), 66-75. doi:10.1016/j.bpsc.2021.03.014 |
| Gladstone | 2017 | Irritability not appropriately measured | Gladstone, T. R., Kaushal, S. A., Bertschinger, E. J., Tudor, M. E., & Sukhodolsky, D. G. (2017). Irritability in children with autism referred for treatment of anxiety or aggression. *Journal of the American Academy of Child & Adolescent Psychiatry,* *56*(10), S258. doi:10.1016/j.jaac.2017.09.298 |
| Goh | 2023 | Irritability not appropriately measured | Goh, P. K., Smith, T. E., Lee, C. A., Bansal, P. S., Eng, A. G., & Martel, M. M. (2023). Etiological Networks of Attention-Deficit/Hyperactivity Disorder during Childhood and Adolescence. Journal of Clinical Child and Adolescent Psychology, 52(2), 230–243. doi:10.1080/15374416.2021.1946820 |
| Gotham | 2013 | Irritability not appropriately measured | Gotham, K., Bishop, S. L., Hus, V., Huerta, M., Lund, S., Buja, A., ... & Lord, C. (2013). Exploring the relationship between anxiety and insistence on sameness in autism spectrum disorders. *Autism Research*, *6*(1), 33-41. doi:10.1002/aur.1263 |
| Grabell | 2018 | Mental health outcomes not measured | Grabell, A. S., Li, Y., Barker, J. W., Wakschlag, L. S., Huppert, T. J., & Perlman, S. B. (2018). Evidence of non-linear associations between frustration-related prefrontal cortex activation and the normal: abnormal spectrum of irritability in young children. *Journal of Abnormal Child Psychology, 46*, 137-147. doi:10.1007/s10802-017-0286-5 |
| Green | 2000 | Irritability not appropriately measured | Green, J., Gilchrist, A., Burton, D., & Cox, A. (2000). Social and psychiatric functioning in adolescents with Asperger syndrome compared with conduct disorder. *Journal of Autism and Developmental Disorders, 30*(4), 279-293. doi:10.1023/A:1005523232106 |
| Gustafsson | 2019 | Irritability not appropriately measured | Gustafsson, M. L., Laaksonen, C., Salanterä, S., Löyttyniemi, E., & Aromaa, M. (2019). Associations between daytime sleepiness, psychological symptoms, headache, and abdominal pain in schoolchildren. *The Journal of School Nursing, 35*(4), 279-286. doi:10.1177/1059840518774394 |
| Hafeman | 2023 | Data unavailable | Hafeman, D. M., Merranko, J., Joseph, H. M., Goldstein, T. R., Goldstein, B. I., Levenson, J., Axelson, D., Monk, K., Sakolsky, D., Iyengar, S., & Birmaher, B. (2023). Early indicators of bipolar risk in preschool offspring of parents with bipolar disorder. Journal of Child Psychology and Psychiatry, 64(10), 1492–1500. doi:10.1111/jcpp.13739 |
| Haller | 2020 | Mental health outcomes not measured | Haller, S. P., Kircanski, K., Stringaris, A., Clayton, M., Bui, H., Agorsor, C., Cardenas, S. I., Towbin, K. E., Pine, D. S., Leibenluft, E., & Brotman, M. A. (2020). The clinician affective reactivity index: Validity and reliability of a clinician-rated assessment of irritability. *Behavior Therapy, 51*(2), 283-293. doi:10.1016/j.beth.2019.10.005 |
| Haller | 2021 | Data unavailable | Haller, S. P., Stoddard, J., Pagliaccio, D., Bui, H., MacGillivray, C., Jones, M., & Brotman, M. A. (2021). Computational modeling of attentional impairments in disruptive mood dysregulation and attention-deficit/hyperactivity disorder. *Journal of the American Academy of Child and Adolescent Psychiatry, 60*(5), 637-645. doi:10.1016/j.jaac.2020.08.468 |
| Hameed | 2020 | Mental health outcomes not measured | Hameed, U., Dellasega, C., & Scandinaro, A. (2020). Assessment of irritability in school-aged children by pediatric, family practice, and psychiatric providers. *Clinical Child Psychology and Psychiatry, 25*(2), 333-345. doi:10.1177/1359104519865591 |
| Hartman | 2019 | Irritability not appropriately measured | Hartman, C. A., Rommelse, N., van der Klugt, C. L., Wanders, R. B., & Timmerman, M. E. (2019). Stress exposure and the course of ADHD from childhood to young adulthood: comorbid severe emotion dysregulation or mood and anxiety problems. *Journal of Clinical Medicine, 8*(11), 1824. doi:10.3390/jcm8111824 |
| Hart | 2017 | Data unavailable | Harty, S. C., Gnagy, E. M., Pelham, W. E., & Molina, B. S. G. (2017). Anger-irritability as a mediator of attention deficit hyperactivity disorder risk for adolescent alcohol use and the contribution of coping skills. *Journal of Child Psychology and Psychiatry, 58*(5), 555-563. doi:10.1111/jcpp.12668 |
| Hawks | 2020 | Data unavailable | Hawks, J. L., Kennedy, S. M., Holzman, J. B. W., & Ehrenreich-May, J. (2020). Development and application of an innovative transdiagnostic treatment approach for pediatric irritability. *Behavior Therapy, 51*(2), 334-349. doi:10.1016/j.beth.2019.07.004 |
| Hernandez | 2017 | Irritability not appropriately measured | Hernandez, M., Marangoni, C., C Grant, M., Estrada, J., & Faedda, G. L. (2017). Parental reports of prodromal psychopathology in pediatric bipolar disorder. *Current Neuropharmacology, 15*(3), 380-385. doi:10.2174/1570159x14666160801162046 |
| Hirneth | 2015 | Irritability not appropriately measured | Hirneth, S. J., Hazell, P. L., Hanstock, T. L., & Lewin, T. J. (2015). Bipolar disorder subtypes in children and adolescents: demographic and clinical characteristics from an Australian sample. *Journal of Affective Disorders, 175*, 98-107. doi:10.1016/j.jad.2014.12.021 |
| Hirsch | 2023 | Mental health outcomes not measured | Hirsch, E., Alam, T., Kirk, N., Bevans, K. B., Briggs‐Gowan, M., Wakschlag, L. S., Wiggins, J. L., & Roy, A. K. (2023). Developmentally specified characterization of the irritability spectrum at early school age: Implications for pragmatic mental health screening. International Journal of Methods in Psychiatric Research, 32(S1), e1985–e1985. doi:10.1002/mpr.1985 |
| Humphreys | 2019 | Data unavailable | Humphreys, K. L., Schouboe, S. N., Kircanski, K., Leibenluft, E., Stringaris, A., & Gotlib, I. H. (2019). Irritability, externalizing, and internalizing psychopathology in adolescence: Cross-sectional and longitudinal associations and moderation by sex. *Journal of Clinical Child & Adolescent Psychology, 48*(5), 781-789. doi:10.1080/15374416.2018.1460847 |
| Hunt | 2009 | Irritability not appropriately measured | Hunt, J., Birmaher, B., Leonard, H., Strober, M., Axelson, D., Ryan, N., Yang, M., Gill, M., Dyl, J., Esposito-Smythers, C., Swenson, L., Goldstein, B., Goldstein, T., Stout, R., & Keller, M. (2009). Irritability without elation in a large bipolar youth sample: frequency and clinical description. *Journal of the American Academy of Child & Adolescent Psychiatry, 48*(7), 730-739. doi:10.1097/CHI.0b013e3181a565db |
| Hunt | 2013 | Irritability not appropriately measured | Hunt, J. I., Case, B. G., Birmaher, B., Stout, R. L., Dickstein, D. P., Yen, S., Goldstein, T. R., Goldstein, B. I., Axelson, D. A., Hower, H., Strober, M., Ryan, N., Swenson, L., Topor, D. R., Gill, M. K., Weinstock, L. M., & Keller, M. B. (2013). Irritability and elation in a large bipolar youth sample: relative symptom severity and clinical outcomes over 4 years. *The Journal of Clinical Psychiatry, 74*(1), 9850. doi:10.4088/JCP.12m07874 |
| Hwang | 2021 | Mental health outcomes not measured | Hwang, S., Chung, U., Chang, Y., Kim, E., Suk, J. W., Meffert, H., Kratochvil, C., Leibenluft, E., & Blair, J. (2021). Neural responses to fluoxetine in youths with disruptive behavior and trauma exposure: a pilot study. *Journal of Child and Adolescent Psychopharmacology, 31*(8), 562-571. doi:10.1089/cap.2020.0174 |
| Ishii-Takahashi | 2018 | Mental health outcomes not measured | Ishii-Takahashi, A., Faria, A. V., Mangalmurti, A., Sudre, G., Sharp, W., Choudhury, S., Mori, S., & Shaw, W. P. (2018,). Neural mechanisms of irritability in children with ADHD. *Journal of the American Academy of Child & Adolescent Psychiatry, 57*(10), S144. doi:10.1016/j.jaac.2018.09.042 |
| Johns-Mead | 2023 | Mental health outcomes not measured | Johns-Mead, R., Vijayakumar, N., Mulraney, M., Melvin, G., Youssef, G., Sciberras, E., Anderson, V. A., Nicholson, J. M., Efron, D., Hazel, P., & Silk, T. J. (2023). Categorical and dimensional approaches to the developmental relationship between ADHD and irritability. Journal of Child Psychology and Psychiatry, 64(10), 1422–1431. doi:10.1111/jcpp.13818 |
| Jones | 2013 | Irritability not appropriately measured | Jones, A. M., De Nadai, A. S., Arnold, E. B., McGuire, J. F., Lewin, A. B., Murphy, T. K., & Storch, E. A. (2013). Psychometric properties of the obsessive-compulsive inventory: child version in children and adolescents with obsessive–compulsive disorder. *Child Psychiatry & Human Development, 44*, 137-151. doi:10.1007/s10578-012-0315-0 |
| Joseph | 2019 | Irritability not appropriately measured | Joseph, C. I., Evans, S., Youssef, G. J., Silk, T., Anderson, V., Efron, D., & Sciberras, E. (2019). Characterisation of depressive symptoms in young children with and without attention deficit hyperactivity disorder. *European Child & Adolescent Psychiatry, 28*, 1183-1192. doi:10.1007/s00787-018-01274-5 |
| Junghänel | 2022 | Non-empirical article | Junghänel, M., Thoene, A. K., Ginsberg, C., Goertz-Dorten, A., Frenk, F., Muecke, K., Treier, A. K., Labarga, S. Z., Banaschewski, T., Millenet, S., Fegert, J. M., Bernheim, D., Hanisch, C., Kvolch, M., Schvoller, A., Ravens-Sieberer, U., Kaman, A., Roessner, V., Hinz, J., & Doepfner, M. (2022). Irritability and emotional impulsivity as core feature of ADHD and ODD in children. *Journal of Psychopathology and Behavioral Assessment, 44*(3), 679-697. doi:10.1007/s10862-022-09974-8 |
| Kaat | 2019 | Mental health outcomes not measured | Kaat, A. J., Blackwell, C. K., Estabrook, R., Burns, J. L., Petitclerc, A., Briggs-Gowan, M. J., Gershon, R. C., Cella, D., Perlman, S. B., & Wakschlag, L. S. (2019). Linking the Child Behavior Checklist (CBCL) with the Multidimensional Assessment Profile of Disruptive Behavior (MAP-DB): Advancing a dimensional spectrum approach to disruptive behavior. *Journal of Child and Family Studies, 28*, 343-353. doi:10.1007/s10826-018-1272-4 |
| Karalunas | 2021 | Irritability not appropriately measured | Karalunas, S. L., Antovich, D., Goh, P. K., Martel, M. M., Tipsord, J., Nousen, E. K., & Nigg, J. T. (2021). Longitudinal network model of the co-development of temperament, executive functioning, and psychopathology symptoms in youth with and without ADHD. *Development and Psychopathology, 33*(5), 1803-1820. doi:10.1017/s0954579421000900 |
| Karim | 2017 | Mental health outcomes not measured | Karim, H. T., & Perlman, S. B. (2017). Neurodevelopmental maturation as a function of irritable temperament: Insights from a naturalistic emotional video viewing paradigm. *Human Brain Mapping, 38(*10), 5307-5321. doi:10.1002/hbm.23742 |
| Kaurin | 2024 | Data duplicated or not suitable | Kaurin, A., Wright, A. G. C., Porta, G., Hamilton, E., Poling, K., Bero, K., Brent, D., & Goldstein, T. R. (2024). Weekly links among irritability and suicidal thoughts and behaviors in high-risk youth. Journal of Child Psychology and Psychiatry. doi:10.1111/jcpp.13988 |
| Kazdin | 1987 | Irritability not appropriately measured | Kazdin, A. E., Rodgers, A., Colbus, D., & Siegel, T. (1987). Children's hostility inventory: Measurement of aggression and hostility in psychiatric inpatient children. *Journal of Clinical Child Psychology, 16*(4), 320-328. doi:10.1207/s15374424jccp1604_5 |
| Kennard | 2018 | Irritability not appropriately measured | Kennard, B. D., Mayes, T. L., Chahal, Z., Nakonezny, P. A., Moorehead, A., & Emslie, G. J. (2018). Predictors and moderators of relapse in children and adolescents with major depressive disorder. *The Journal of Clinical Psychiatry, 79*(2), 15121. doi:10.4088/JCP.15m10330 |
| Kessel | 2021 | Mental health outcomes not measured | Kessel, E. M., Dougherty, L. R., Hubachek, S., Chad-Friedman, E., Olino, T., Carlson, G. A., & Klein, D. N. (2021). Early predictors of adolescent irritability. *Child and Adolescent Psychiatric Clinics, 30*(3), 475-490. doi:10.1016/j.chc.2021.04.002 |
| Kiff | 2011 | Non-empirical article | Kiff, C. J., Lengua, L. J., & Zalewski, M. (2011). Nature and nurturing: Parenting in the context of child temperament. *Clinical Child and Family Psychology Review, 14*(3), 251-301. doi:10.1007/s10567-011-0093-4 |
| Kircanski | 2021 | Mental health outcomes not measured | Kircanski, K., Linke, J., & Leibenluft, E. (2021). Brain mechanisms of frustrative non-reward: Implications for pediatric irritability. *Biological Psychiatry, 89*(9), S46-S47. doi:10.1016/j.biopsych.2021.02.133 |
| Kircanski | 2017 | Mental health outcomes not measured | Kircanski, K., White, L., Tseng, W. L., Wiggins, J., Frank, H., Sequeira, S., Zhang, S. S., Towbin, K., Stringaris, A., Pine, D., Leibenluft, E., & Brotman, M. (2017). Computational phenotyping reveals a double dissociation in the neural mechanisms of irritability and anxiety in youth. *Neuropsychopharmacology. 43*(1) S302-S303. doi:10.1038/npp.2017.265 |
| Kircanski | 2018 | Data unavailable | Kircanski, K., White, L. K., Tseng, W. L., Wiggins, J. L., Frank, H. R., Sequeira, S., Zhang, S., Abend, R., Towbin, K. E., Stringaris, A., Pine, D. S., Leibenluft, E., & Brotman, M. A. (2018). A latent variable approach to differentiating neural mechanisms of irritability and anxiety in youth. *JAMA Psychiatry, 75*(6), 631-639. doi:10.1001/jamapsychiatry.2018.0468 |
| Kircanski | 2017 | Data unavailable | Kircanski, K., Zhang, S., Stringaris, A., Wiggins, J. L., Towbin, K. E., Pine, D. S., Leibenluft, E., & Brotman, M. A. (2017). Empirically derived patterns of psychiatric symptoms in youth: A latent profile analysis. *Journal of Affective Disorders, 216*, 109-116. doi:10.1016/j.jad.2016.09.016 |
| Kouros | 2016 | Irritability not appropriately measured | Kouros, C. D., Morris, M. C., & Garber, J. (2016). Within-person changes in individual symptoms of depression predict subsequent depressive episodes in adolescents: a prospective study. *Journal of Abnormal Child Psychology, 44*, 483-494. doi:10.1007/s10802-015-0046-3 |
| Kryza-Lacombe | 2021 | Mental health outcomes not measured | Kryza‐Lacombe, M., Hernandez, B., Owen, C., Reynolds, R. C., Wakschlag, L. S., Dougherty, L. R., & Wiggins, J. L. (2021). Neural mechanisms of reward processing in adolescent irritability. *Developmental Psychobiology, 63*(5), 1241-1254. doi:10.1002/dev.22090 |
| Leadbeater | 2015 | Data unavailable | Leadbeater, B. J., & Homel, J. (2015). Irritable and defiant sub-dimensions of ODD: Their stability and prediction of internalizing symptoms and conduct problems from adolescence to young adulthood. *Journal of Abnormal Child Psychology, 43*(3), 407-421. doi:10.1007/s10802-014-9908-3 |
| Leadbeater | 2023 | Data duplicated or not suitable | Leadbeater, B. J., Merrin, G. J., Contreras, A., & Ames, M. E. (2023). Trajectories of oppositional defiant disorder severity from adolescence to young adulthood and substance use, mental health, and behavioral problems. Journal of the Canadian Academy of Child and Adolescent Psychiatry, 32(4), 224–235. |
| Lecavalier | 2006 | Irritability not appropriately measured | Lecavalier, L. (2006). Behavioral and emotional problems in young people with pervasive developmental disorders: Relative prevalence, effects of subject characteristics, and empirical classification. *Journal of Autism and Developmental Disorders, 36*(8), 1101-1114. doi:10.1007/s10803-006-0147 |
| Lecavalier | 2019 | Irritability not appropriately measured | Lecavalier, L., McCracken, C. E., Aman, M. G., McDougle, C. J., McCracken, J. T., Tierney, E., Smith, T., Johnson, C., King, B., Handen, B., Swiezy, N. B., Arnold, L. E., Bearss, K., Vitiello, B., & Scahill, L. (2019). An exploration of concomitant psychiatric disorders in children with autism spectrum disorder. *Comprehensive Psychiatry, 88*, 57-64. doi:10.1016/j.comppsych.2018.10.012 |
| Lee | 2022 | Mental health outcomes not measured | Lee, K. S., Xiao, J., Liew, Z., Gau, S. S. F., & Tseng, W. L. (2022). Perinatal and birth correlates of childhood irritability in Taiwan's national epidemiological study. *Journal of Affective Disorders, 299*, 273-280. doi:10.1016/j.jad.2021.12.016 |
| Lee | 2017 | Irritability not appropriately measured | Lee, M., Aggen, S. H., Carney, D. M., Hahn, S., Moroney, E., Machlin, L., Brotman, M. A., Towbin, K. E., Leibenluft, E., Pine, D. S., Roberson-Nay, R., & Hettema, J. M. (2017). Latent structure of negative valence measures in childhood. *Depression and Anxiety, 34*(8), 742-751. doi:10.1002/da.22656 |
| Lengua | 2003 | Irritability not appropriately measured | Lengua, L. J. (2003). Associations among emotionality, self-regulation, adjustment problems, and positive adjustment in middle childhood. *Journal of Applied Developmental Psychology, 24*(5), 595-618. doi:10.1016/j.appdev.2003.08.002 |
| Levy | 2018 | Data unavailable | Levy, T., Kronenberg, S., & Schachar, R. (2018). 2.47 ADHD and suicidal ideation among children and adolescents: The mediating role of depression and irritability. *Journal of the American Academy of Child and Adolescent Psychiatry*, *57*(10), S174–S174. doi:10.1016/j.jaac.2018.09.135 |
| Li | 2017 | Mental health outcomes not measured | Li, Y., Grabell, A. S., Wakschlag, L. S., Huppert, T. J., & Perlman, S. B. (2017). The neural substrates of cognitive flexibility are related to individual differences in preschool irritability: A fNIRS investigation. *Developmental Cognitive Neuroscience, 25,* 138-144. doi:10.1016/j.dcn.2016.07.002 |
| Li | 2023 | Data duplicated or not suitable | Li, Y., Tian, W., Liu, P., & Geng, F. (2023). A cross-sectional analysis of the relationships between anxiety sensitivity and youth irritability: the mediated roles of insomnia and selective attention for threat. BMC Psychiatry, 23(1), 1–782. doi:10.1186/s12888-023-05280-z |
| Linke | 2019 | Non-empirical article | Linke, J. O., Jones, E., Pagliaccio, D., Swetlitz, C., Lewis, K. M., Silverman, W. K., Bar-Haim, Y., Pine, D. S., & Brotman, M. A. (2019). Efficacy and mechanisms underlying a gamified attention bias modification training in anxious youth: protocol for a randomized controlled trial. *BMC Psychiatry, 19*(1), 1-12. doi:10.1186/s12888-019-2224-2 |
| Liu | 2023 | Mental health outcomes not measured | Liu, C., Zheng, Y., Ganiban, J. M., & Saudino, K. J. (2023). Genetic and environmental influences on temperament development across the preschool period. Journal of Child Psychology and Psychiatry, 64(1), 59–70. doi:10.1111/jcpp.13667 |
| Liuzzi | 2020 | Mental health outcomes not measured | Liuzzi, M. T., Kryza-Lacombe, M., Christian, I. R., Palumbo, D. E., Amir, N., & Wiggins, J. L. (2020). Neural and behavioral correlates of inhibitory control in youths with varying levels of irritability. *Journal of Affective Disorders, 273*, 567-575. doi:10.1016/j.jad.2020.04.049 |
| Lobato Machado | 2022 | Irritability not appropriately measured | Lobato Machado, M. C., Hibner, M. E. R. B., Nogueira, D. B., Rezende, M. M., Bórem, I. L., da Cunha, L. R., & Marques de Miranda, D. (2022). Irritability in an open-door pediatric psychiatric emergency service in a middle-income country. Neuropsychiatrie de l’enfance et de l’adolescence, 70(6), 336–342. doi:10.1016/j.neurenf.2022.05.007 |
| Lozano | 2019 | Irritability not appropriately measured | Lozano, L. M., Valor-Segura, I., García-Cueto, E., Pedrosa, I., Llanos, A., & Lozano, L. (2019). Relationship between child perfectionism and psychological disorders. *Frontiers in Psychology, 10*, 1855. doi:10.3389/fpsyg.2019.01855 |
| Luby | 2019 | Irritability not appropriately measured | Luby, J. L., Whalen, D., Tillman, R., & Barch, D. M. (2019). Clinical and psychosocial characteristics of young children with suicidal ideation, behaviors, and non-suicidal self-injurious behaviors. *Journal of the American Academy of Child & Adolescent Psychiatry, 58*(1), 117-127. doi:10.1016/j.jaac.2018.06.031 |
| Marsh | 2018 | Data unavailable | Marsh, C. L., Ibrahim, K., Gladstone, T. R., & Sukhodolsky, D. G. (2018). 5.27 Discrepancies between parent ratings and child self-report of anxiety and irritability in children With ASD. *Journal of the American Academy of Child and Adolescent Psychiatry*, *57*(10), S235–S235. doi:10.1016/j.jaac.2018.09.320 |
| Mayes | 2019 | Data unavailable | Mayes, S. D., Calhoun, S. L., Waxmonsky, J. G., Kokotovich, C., Baweja, R., Lockridge, R., & Bixler, E. O. (2019). Demographic differences in disruptive mood dysregulation disorder symptoms in ADHD, autism, and general population samples. *Journal of Attention Disorders, 23*(8), 849-858. doi:10.1177/1087054716664409 |
| Mayes | 2017 | Data unavailable | Mayes, S. D., Kokotovich, C., Mathiowetz, C., Baweja, R., Calhoun, S. L., & Waxmonsky, J. (2017). Disruptive mood dysregulation disorder symptoms by age in autism, ADHD, and general population samples. *Journal of Mental Health Research in Intellectual Disabilities, 10*(4), 345-359. doi:10.1080/19315864.2017.1338804 |
| Mayes | 2016 | Data unavailable | Mayes, S. D., Waxmonsky, J. D., Calhoun, S. L., & Bixler, E. O. (2016). Disruptive mood dysregulation disorder symptoms and association with oppositional defiant and other disorders in a general population child sample*. Journal of Child and Adolescent Psychopharmacology, 26*(2), 101-106. doi:10.1089/cap.2015.0074 |
| Melvin | 2018 | Mental health outcomes not measured | Melvin, G. A., Tonge, B. J., Mulraney, M., Gordon, M., Taffe, J., & Klimkeit, E. (2018). The cranky thermometers: Visual analogue scales measuring irritability in youth. *Journal of Adolescence, 64*, 146-154. doi:10.1016/j.adolescence.2018.02.008 |
| Menezes | 2021 | Irritability not appropriately measured | Menezes, M., & Mazurek, M. O. (2021). Associations between domains of health-related quality of life and co-occurring emotional and behavioral problems in youth with autism spectrum disorder. *Research in Autism Spectrum Disorders, 82*, 101740. doi:10.1016/j.rasd.2021.101740 |
| Mick | 2005 | Mental health outcomes not measured | Mick, E., Spencer, T., Wozniak, J., & Biederman, J. (2005). Heterogeneity of irritability in attention-deficit/hyperactivity disorder subjects with and without mood disorders. *Biological Psychiatry, 58*(7), 576-582. doi:10.1016/j.biopsych.2005.05.037 |
| Mikita | 2015 | Irritability not appropriately measured | Mikita, N., Hollocks, M. J., Papadopoulos, A. S., Aslani, A., Harrison, S., Leibenluft, E., Simonoff, E., & Stringaris, A. (2015). Irritability in boys with autism spectrum disorders: an investigation of physiological reactivity. *Journal of Child Psychology and Psychiatry, 56*(10), 1118-1126. doi:10.1111/jcpp.12382 |
| Miller | 2018 | Mental health outcomes not measured | Miller, L., Hlastala, S. A., Mufson, L., Leibenluft, E., Yenokyan, G., & Riddle, M. (2018). Interpersonal psychotherapy for mood and behavior dysregulation: Pilot randomized trial. *Depression and Anxiety, 35*(6), 574-582. doi:10.1002/da.22761 |
| Mohamed | 2023 | Data duplicated or not suitable | Mohamed, A. K., Croarkin, P. E., Jha, M. K., & Vande Voort, J. L. (2023). Early reduction in irritability is associated with improved outcomes among youth with depression: Findings from the AMOD study. Journal of Affective Disorders, 324, 77–81. doi:10.1016/j.jad.2022.12.031 |
| Mufson | 2002 | Sample outside age range | Mufson, L., Nomura, Y., & Warner, V. (2002). The relationship between parental diagnosis, offspring temperament and offspring psychopathology: A longitudinal analysis. *Journal of Affective Disorders, 71*(1-3), 61-69. doi:10.1016/s0165-0327(01)00375-5 |
| Mulraney | 2016 | Data unavailable | Mulraney, M., Schilpzand, E. J., Hazell, P., Nicholson, J. M., Anderson, V., Efron, D., Silk, T. J., & Sciberras, E. (2016). Comorbidity and correlates of disruptive mood dysregulation disorder in 6-8-year-old children with ADHD. *European Child & Adolescent Psychiatry, 25*(3), 321-330. doi:10.1007/s00787-015-0738-9 |
| Mulraney | 2014 | Sample outside age range | Mulraney, M. A., Melvin, G. A., & Tonge, B. J. (2014). Psychometric properties of the affective reactivity index in Australian adults and adolescents. *Psychological Assessment, 26*(1), 148. doi:10.1037/a0034891 |
| Naim | 2021 | Mental health outcomes not measured | Naim, R., Goodwin, M. S., Dombek, K., Revzina, O., Agorsor, C., Lee, K., Zapp, C., Freitag, G. F., Haller, S. P., Cardinale, E., Jangraw, D., & Brotman, M. A. (2021). Cardiovascular reactivity as a measure of irritability in a transdiagnostic sample of youth: Preliminary associations*. International* *Journal of Methods in Psychiatric Research, 30*(4), e1890. doi:10.1002/mpr.1890 |
| Neuhaus | 2019 | Irritability not appropriately measured | Neuhaus, E., Webb, S. J., & Bernier, R. A. (2019). Linking social motivation with social skill: The role of emotion dysregulation in autism spectrum disorder. *Development and Psychopathology, 31*(3), 931-943. doi:10.1017/s0954579419000361 |
| Nielsen | 2021 | Non-empirical article | Nielsen, A. N., Wakschlag, L. S., & Norton, E. S. (2021). Linking irritability and functional brain networks: A transdiagnostic case for expanding consideration of development and environment in RDoC. *Neuroscience and Biobehavioral Reviews, 129*, 231-244. doi:10.1016/j.neubiorev.2021.07.022 |
| Nili | 2022 | Data unavailable | Nili, A. N., Krogh-Jespersen, S., Perlman, S. B., Estabrook, R., Petitclerc, A., Briggs-Gowan, M. J., Sherlock, P. R., Norton, E. S., & Wakschlag, L. S. (2022). Joint Consideration of Inhibitory Control and Irritability in Young Children: Contributions to Emergent Psychopathology. Journal of Abnormal Child Psychology, 50(11), 1415–1427. doi:10.1007/s10802-022-00945-x |
| Nimmagadda | 2011 | Irritability not appropriately measured | Nimmagadda, S. R., Agrawal, N., Worrall-Davies, A., Markova, I., & Rickards, H. (2011). Determinants of irritability in Huntington's disease. *Acta Neuropsychiatrica, 23*(6), 309-314. doi:10.1111/j.1601-5215.2011.00563.x |
| Olson | 2005 | Irritability not appropriately measured | Olson, S. L., Sameroff, A. J., Kerr, D. C., Lopez, N. L., & Wellman, H. M. (2005). Developmental foundations of externalizing problems in young children: The role of effortful control. *Development and Psychopathology, 17*(1), 25-45. doi:10.1017/s0954579405050029 |
| Orengul | 2019 | Irritability not appropriately measured | Örengül, A. C., Ucuz, İ., Oner Battaloglu, N., Ozek, G., & Gormez, V. (2019). Prevalence of psychiatric disorders and suicidality among children and adolescents with thalassemia major—A Turkish sample. *Children's Health Care, 48*(1), 120-129. doi:10.1080/02739615.2018.1500290 |
| Orri | 2019 | Data unavailable | Orri, M., Galera, C., Turecki, G., Boivin, M., Tremblay, R. E., Geoffroy, M.-C., & Côté, S. M. (2019). Pathways of association between childhood irritability and adolescent suicidality. *Journal of the American Academy of Child and Adolescent Psychiatry, 58*(1), 99-107.e103. doi:10.1016/j.jaac.2018.06.034 |
| Orri | 2018 | Data unavailable | Orri, M., Galera, C., Turecki, G., Forte, A., Renaud, J., Boivin, M., Tremblay, R. E., Côté, S. M., & Geoffroy, M.-C. (2018). Association of childhood irritability and depressive/anxious mood profiles with adolescent suicidal ideation and attempts. *JAMA Psychiatry, 75*(5), 465-473. doi:10.1001/jamapsychiatry.2018.0174 |
| Pagliaccio | 2018 | Data unavailable | Pagliaccio, D., Pine, D. S., Barch, D. M., Luby, J. L., & Leibenluft, E. (2018). Irritability trajectories, cortical thickness, and clinical outcomes in a sample enriched for preschool depression. *Journal of the American Academy of Child and Adolescent Psychiatry, 57*(5), 336. doi:10.1016/j.jaac.2018.02.010 |
| Pagliaccio | 2017 | Mental health outcomes not measured | Pagliaccio, D., Wiggins, J. L., Adleman, N. E., Curhan, A., Zhang, S., Towbin, K. E., Brotman, M. A., Pine, D. S., & Leibenluft, E. (2017). Behavioral and neural sustained attention deficits in disruptive mood dysregulation disorder and attention-deficit/hyperactivity disorder. *Journal of the American Academy of Child & Adolescent Psychiatry, 56*(5), 426-435. doi:10.1016/j.jaac.2017.02.008 |
| Perhamus | 2017 | Mental health outcomes not measured | Perhamus, G., Kircanski, K., Wiggins, J. L., Stiles, K., Curhan, A., Wambach, C., Towbin, K., Pine, D. S., Brotman, M. A., & Leibenluft, E. (2017). Face emotion labeling in pediatric irritability: behavioral and neural correlates. *Journal of the American Academy of Child & Adolescent Psychiatry, 56*(10), S245-S246. doi:10.1016/j.jaac.2017.09.266 |
| Perlman | 2015 | Mental health outcomes not measured | Perlman, S. B., Jones, B. M., Wakschlag, L. S., Axelson, D., Birmaher, B., & Phillips, M. L. (2015). Neural substrates of child irritability in typically developing and psychiatric populations. *Developmental Cognitive Neuroscience, 14*, 71-80. doi:10.1016/j.dcn.2015.07.003 |
| Piguet | 2021 | Mental health outcomes not measured | Piguet, C., Mihailov, A., Grigis, A., Laidi, C., Duchesnay, E., & Houenou, J. (2021). Irritability Is associated with decreased cortical surface area and anxiety with decreased gyrification during brain development. *Frontiers in Psychiatry, 12*, 744419. doi:10.3389/fpsyt.2021.744419 |
| Piotrowska | 2015 | Irritability not appropriately measured | Piotrowska, P. J., Stride, C. B., Maughan, B., Goodman, R., McCaw, L., & Rowe, R. (2015). Income gradients within child and adolescent antisocial behaviours. *The British Journal of Psychiatry, 207*(5), 385-391. doi:10.1192/bjp.bp.113.143636 |
| Piotrowska | 2019 | Mental health outcomes not measured | Piotrowska, P. J., Stride, C. B., Maughan, B., & Rowe, R. (2019). Mechanisms underlying social gradients in child and adolescent antisocial behaviour. *SSM-Population Health, 7*, 100353. doi:10.1016/j.ssmph.2019.100353 |
| Poli | 2003 | Irritability not appropriately measured | Poli, P., Sbrana, B., Marcheschi, M., & Masi, G. (2003). Self-reported depressive symptoms in a school sample of Italian children and adolescents. *Child Psychiatry and Human Development, 33*, 209-226. doi:10.1023/a:1021404613832 |
| Prinzie | 2014 | Irritability not appropriately measured | Prinzie, P., van Harten, L. V., Deković, M., van den Akker, A. L., & Shiner, R. L. (2014). Developmental trajectories of anxious and depressive problems during the transition from childhood to adolescence: personality× parenting interactions. *Development and Psychopathology, 26*(4pt1), 1077-1092. doi:10.1017/s0954579414000510 |
| Quarmley | 2023 | Sample outside age range | Quarmley, M., Vafiadis, A., & Jarcho, J. M. (2023). Irritability and rejection‐elicited aggression in adolescents and young adults. Journal of Child Psychology and Psychiatry, 64(9), 1346–1358. doi:10.1111/jcpp.13804 |
| Rasan | 2013 | Irritability not appropriately measured | Rosan, A. M., & Costea-Barlutiu, C. (2013). Associations between callous-unemotional traits, aggression and psychopathology in detained adolescent males. *Journal of Cognitive and Behavioral Psychotherapies, 13*(2), 397-407. |
| Ravi | 2022 | Mental health outcomes not measured | Ravi, S., Havewala, M., Kircanski, K., Brotman, M. A., Schneider, L., Degnan, K., Almas, A., Fox, N., Pine, D. S., Leibenluft, E., & Filippi, C. (2022). Parenting and childhood irritability: Negative emotion socialization and parental control moderate the development of irritability. *Development and Psychopathology*, 1-10. doi:10.1017/s0954579421001346 |
| Read | 2020 | Data unavailable | Read, N., Mulraney, M., McGillivray, J., & Sciberras, E. (2020). Comorbid anxiety and irritability symptoms and their association with cognitive functioning in children with ADHD. *Journal of Abnormal Child Psychology, 48*(8), 1035-1046. doi:10.1007/s10802-020-00658-z |
| Riglin | 2017 | Irritability not appropriately measured | Riglin, L., Collishaw, S., Richards, A., Thapar, A. K., Maughan, B., O'Donovan, M. C., & Thapar, A. (2017). Schizophrenia risk alleles and neurodevelopmental outcomes in childhood: a population-based cohort study. *The Lancet Psychiatry, 4*(1), 57-62. doi:10.1016/S2215-0366(16)30406-0 |
| Riglin | 2017 | Data unavailable | Riglin, L., Eyre, O., Cooper, M., Collishaw, S., Martin, J., Langley, K., Leibenluft, E., Stringaris, A., Thapar, A. K., Maughan, B., O'Donovan, M. C., & Thapar, A. (2017). Investigating the genetic underpinnings of early-life irritability. *Translational Psychiatry, 7*(9), 1241. doi:10.1038/tp.2017.212 |
| Riglin | 2019 | Mental health outcomes not measured | Riglin, L., Eyre, O., Thapar, A. K., Stringaris, A., Leibenluft, E., Pine, D. S., Tilling, K., Davey Smith, G., O'Donovan, M. C., & Thapar, A. (2019). Identifying novel types of irritability using a developmental genetic approach. *American Journal of Psychiatry, 176*(8), 635-642. doi:10.1176/appi.ajp.2019.18101134 |
| Roberson-Nay | 2015 | Mental health outcomes not measured | Roberson-Nay, R., Leibenluft, E., Brotman, M. A., Myers, J., Larsson, H., Lichtenstein, P., & Kendler, K. S. (2015). Longitudinal stability of genetic and environmental influences on irritability: From childhood to young adulthood. *American Journal of Psychiatry, 172*(7), 657-664. doi:10.1176/appi.ajp.2015.14040509 |
| Rothbart | 2001 | Irritability not appropriately measured | Rothbart, M. K., Ahadi, S. A., Hershey, K. L., & Fisher, P. (2001). Investigations of temperament at three to seven years: The children's behavior questionnaire. *Child Development, 72*(5), 1394-1408. doi:10.1111/1467-8624.00355 |
| Rozanov | 2014 | Irritability not appropriately measured | Rozanov, V. A., Rakhimkulova, A. V., & Ukhanova, A. I. (2014). "Life has no meaning” feelings in adolescents—Relation to suicidal ideation and attempts and mental health. *Suicidology 5*(3), 16. |
| Sachs | 2017 | Mental health outcomes not measured | Sachs, J. F., Filippi, C. A., Kircanski, K., Pacheco, J., Gold, A. L., Sylvester, C. M., Pagliaccio, D., Abend, R., Fox, N.A., Pine, D.S. & Leibenluft, E. (2017). Investigating how irritability across development is related to brain structure in middle childhood. *Journal of the American Academy of Child & Adolescent Psychiatry, 56*(10), S251-S252. doi:10.1016/j.jaac.2017.09.282 |
| Salum | 2017 | Data unavailable | Salum, G. A., Mogg, K., Bradley, B. P., Stringaris, A., Gadelha, A., Pan, P. M., Rohde, L. A., Polanczyk, G. V., Manfro, G. G., Pine, D. S., & Leibenluft, E. (2017). Association between irritability and bias in attention orienting to threat in children and adolescents. *Journal of Child Psychology and Psychiatry, 58*(5), 595-602. doi:10.1111/jcpp.12659 |
| Savage | 2015 | Data unavailable | Savage, J., Verhulst, B., Copeland, W., Althoff, R. R., Lichtenstein, P., & Roberson-Nay, R. (2015). A genetically informed study of the longitudinal relation between irritability and anxious/depressed symptoms*. Journal of the American Academy of Child and Adolescent Psychiatry, 54*(5), 377-384. doi:10.1016/j.jaac.2015.02.010 |
| Scheinost | 2021 | Mental health outcomes not measured | Scheinost, D., Dadashkarimi, J., Finn, E. S., Wambach, C. G., MacGillivray, C., Roule, A. L., Niendam, T. A., Pine, D. S., Brotman, M. A., Leibenluft, E., & Tseng, W. L. (2021). Functional connectivity during frustration: a preliminary study of predictive modeling of irritability in youth. *Neuropsychopharmacology, 46*(7), 1300-1306. doi:10.1038/s41386-020-00954-8 |
| Schettini | 2021 | Irritability not appropriately measured | Schettini, E., Wilson, S., & Beauchaine, T. P. (2021). Internalizing–externalizing comorbidity and regional brain volumes in the ABCD study. *Development and Psychopathology, 33*(5), 1620-1633. doi:10.1017/s0954579421000560 |
| Serra | 2021 | Irritability not appropriately measured | Serra, G., Iannoni, M. E., Trasolini, M., Maglio, G., Frattini, C., Casini, M. P., Baldessarini, R. J., & Vicari, S. (2021). Characteristics associated with depression severity in 270 juveniles in a major depressive episode. *Brain Sciences, 11*(4), 440. doi:10.3390/brainsci11040440 |
| Sevgen | 2017 | Sample outside age range | Sevgen, F. H., & Altun, H. (2017). Presenting complaints and psychiatric diagnoses in children aged 0-5 years presented to a child psychiatry and adolescent clinic. *Psychiatry and Behavioral Sciences, 7*(4), 205. doi:10.5455/jmood.20170806023012 |
| Shaw | 2014 | Non-empirical article | Shaw, P., Stringaris, A., Nigg, J., & Leibenluft, E. (2014). Emotion dysregulation in attention deficit hyperactivity disorder. *The American Journal of Psychiatry, 171*(3), 276-293. doi:10.1176/appi.ajp.2013.13070966 |
| Sherwood | 2021 | Irritability not appropriately measured | Sherwood, S. N., Youngstrom, J. K., Findling, R. L., Youngstrom, E. A., & Freeman, A. J. (2021). Irritability is associated with illness severity and anhedonia is associated with functional impairment among depressed children and adolescents. *Journal of Child and Adolescent Psychopharmacology, 31*(8), 531-537. doi:10.1089/cap.2021.0018 |
| Shimshoni | 2020 | Data unavailable | Shimshoni, Y., Lebowitz, E. R., Brotman, M. A., Pine, D. S., Leibenluft, E., & Silverman, W. K. (2020). Anxious-irritable children: A distinct subtype of childhood anxiety? *Behavior Therapy, 51*(2), 211-222. doi:10.1016/j.beth.2019.06.005 |
| Silver | 2022 | Data duplicated or not suitable | Silver, J., Bufferd, S. J., Dougherty, L. R., Goldstein, B. L., Carlson, G. A., & Klein, D. N. (2022). Is the distinction between tonic and phasic irritability meaningful in 3-year-old children? *European Child & Adolescent Psychiatry, 32*(9), 1755-1763. doi:10.1007/s00787-022-01995-8 |
| Silver | 2024 | Mental health outcomes not measured | Silver, J., Hawes, M., Dougherty, L., Bufferd, S., Kessel, E., Olino, T., Carlson, G., & Klein, D. (2024). Irritability and Temperament: Concurrent and Prospective Relationships in Childhood and Adolescence. Journal of Clinical Child and Adolescent Psychology, 53(2), 156–168. doi:10.1080/15374416.2023.2286586 |
| Silver | 2023 | Data duplicated or not suitable | Silver, J., Mackin, D. M., Bufferd, S. J., Dougherty, L. R., Goldstein, B. L., Carlson, G. A., & Klein, D. N. (2023). Tonic and phasic irritability in 6‐year‐old children: differential correlates and outcomes. Journal of Child Psychology and Psychiatry, 64(2), 234–243. doi:10.1111/jcpp.13688 |
| Smith | 2017 | Mental health outcomes not measured | Smith, T. E., Lee, C. A., Martel, M. M., & Axelrad, M. E. (2017). ODD symptom network during preschool. *Journal of Abnormal Child Psychology, 45*, 743-748. doi:10.1007/s10802-016-0196-y |
| Sorcher | 2024 | Data duplicated or not suitable | Sorcher, L. K., Silver, J., Chad-Friedman, E., Carlson, G. A., Klein, D. N., & Dougherty, L. R. (2024). Early Predictors and Concurrent Correlates of Tonic and Phasic Irritability in Adolescence. Research on Child and Adolescent Psychopathology, 52(7), 1105–1117. doi:10.1007/s10802-024-01185-x |
| Stoddard | 2017 | Mental health outcomes not measured | Stoddard, J., Jones, M., Haller, S., Towbin, K., Pine, D. S., Brotman, M. A., & Leibenluft, E. (2017). 15.2 Identifying the mechanisms of interpretation bias in irritability. *Journal of the American Academy of Child & Adolescent Psychiatry, 56*(10), S324-S325. doi:10.1016/j.jaac.2017.07.664 |
| Stoddard | 2014 | Data duplicated or not suitable | Stoddard, J., Stringaris, A., Brotman, M. A., Montville, D., Pine, D. S., & Leibenluft, E. (2014). Irritability in Child and adolescent anxiety disorders. *Depression and Anxiety, 31*(7), 566-573. doi:10.1002/da.22151 |
| Stringaris | 2009 | Mental health outcomes not measured | Stringaris, A., & Goodman, R. (2009). Three dimensions of oppositionality in youth. *Journal of Child Psychology and Psychiatry, 50*(3), 216-223. doi:10.1111/j.1469-7610.2008.01989.x |
| Stringaris | 2013 | Irritability not appropriately measured | Stringaris, A., Maughan, B., Copeland, W. S., Costello, E. J., & Angold, A. (2013). Irritable mood as a symptom of depression in youth: prevalence, developmental, and clinical correlates in the Great Smoky Mountains Study. *Journal of the American Academy of Child & Adolescent Psychiatry, 52*(8), 831-840. doi:10.1016/j.jaac.2013.05.017 |
| Stupica | 2011 | Sample outside age range | Stupica, B., Sherman, L. J., & Cassidy, J. (2011). Newborn irritability moderates the association between infant attachment security and toddler exploration and sociability. *Child Development, 82*(5), 1381-1389. doi:10.1111/j.1467-8624.2011.01638.x |
| Sugaya | 2022 | Data unavailable | Sugaya, L. S., Kircanski, K., Stringaris, A., Polanczyk, G. V., & Leibenluft, E. (2022). Validation of an irritability measure in preschoolers in school-based and clinical Brazilian samples. *European Child and Adolescent Psychiatry, 31*(4), 577-587. doi:10.1007/s00787-020-01701-6 |
| Theriault | 2014 | Data duplicated or not suitable | Theriault, M. C. G., Lesperance, P., Achim, A., Tellier, G., Diab, S., Rouleau, G. A., Chouinard, S., & Richer, F. (2014). ODD irritability is associated with obsessive-compulsive behavior and not ADHD in chronic tic disorders. *Psychiatry Research, 220*(1-2), 447-452. doi:10.1016/j.psychres.2014.07.039 |
| Towbin | 2020 | Irritability not appropriately measured | Towbin, K., Vidal-Ribas, P., Brotman, M. A., Pickles, A., Miller, K. V., Kaiser, A., Vitale, A. D., Engel, C., Overman, G. P., Davis, M., Lee, B., McNeil, C., Wheeler, W., Yokum, C. H., Haring, C. T., Roule, A., Wambach, C. G., Sharif-Askary, B., Pine, D. S., Leibenluft, E., & Stringaris, A. (2020). A double-blind randomized placebo-controlled trial of citalopram adjunctive to stimulant medication in youth with chronic severe irritability*. Journal of the American Academy of Child & Adolescent Psychiatry, 59*(3), 350-361. doi:10.1016/j.jaac.2019.05.015 |
| Treier | 2024 | Data duplicated or not suitable | Treier, A.-K., Döpfner, M., Ravens-Sieberer, U., Görtz-Dorten, A., Boecker, M., Goldbeck, C., Banaschewski, T., Aggensteiner, P.-M., Hanisch, C., Ritschel, A., Kölch, M., Daunke, A., Roessner, V., Kohls, G., & Kaman, A. (2024). Screening for affective dysregulation in school-aged children: relationship with comprehensive measures of affective dysregulation and related mental disorders. European Child & Adolescent Psychiatry, 33(2), 381–390. doi:10.1007/s00787-023-02166-z |
| Tseliou | 2024 | Data duplicated or not suitable | Tseliou, F., Riglin, L., Thapar, A. K., Heron, J., Dennison, C. A., Armitage, J. M., Thapar, A., Rice, F., & Collishaw, S. (2024). Childhood correlates and young adult outcomes of trajectories of emotional problems from childhood to adolescence. Psychological Medicine, 1–11. doi:10.1017/S0033291724000631 |
| Tseng | 2020 | Mental health outcomes not measured | Tseng, W. L., Abend, R., Gold, A., & Brotman, M. (2020). Parsing distinct and common neural mechanisms of response to learned threat in childhood anxiety and irritability. *Neuropsychopharmacology, 45*(1), 69-70. |
| Tseng | 2021 | Mental health outcomes not measured | Tseng, W. L., Abend, R., Gold, A. L., & Brotman, M. A. (2021). Neural correlates of extinguished threat recall underlying the commonality between pediatric anxiety and irritability. *Journal of Affective Disorders, 295*, 920-929. doi:10.1016/j.jad.2021.08.117 |
| Tseng | 2019 | Mental health outcomes not measured | Tseng, W. L., Deveney, C. M., Stoddard, J., Kircanski, K., Frackman, A. E., Yi, J. Y., Hsu, D., Moroney, E., Machlin, L., Donahue, L., Roule, A., Perhamus, G., Reynolds, R. C., Roberson-Nay, R., Hettema, J. M., Towbin, K. E., Stringaris, A., Pine, D. S., Brotman, M. A., & Leibenluft, E. (2019). Brain mechanisms of attention orienting following frustration: associations with irritability and age in youths. *American Journal of Psychiatry, 176*(1), 67-76. doi:10.1176/appi.ajp.2018.18040491 |
| Tseng | 2015 | Irritability not appropriately measured | Tseng, W. L., Guyer, A. E., Briggs‐Gowan, M. J., Axelson, D., Birmaher, B., Egger, H. L., Helm, J., Stowe, Z., Towbin, K. A., Wakschlag, L. S., Leibenluft, E., & Brotman, M. A. (2015). Behavior and emotion modulation deficits in preschoolers at risk for bipolar disorder. *Depression and Anxiety, 32*(5), 325-334. doi:10.1002/da.22342 |
|  |  |  |  |
| Tseng | 2017 | Mental health outcomes not measured | Tseng, W. L., Moroney, E., Machlin, L., Roberson-Nay, R., Hettema, J. M., Carney, D., Stoddard, J., Towbin, K. A., Pine, D. S., Leibenluft, E., & Brotman, M. A. (2017). Test-retest reliability and validity of a frustration paradigm and irritability measures. *Journal of Affective Disorders, 212*, 38-45. doi:10.1016/j.jad.2017.01.024 |
| Tseng | 2023 | Mental health outcomes not measured | Tseng, W., Naim, R., Chue, A., Shaughnessy, S., Meigs, J., Pine, D. S., Leibenluft, E., Kircanski, K., & Brotman, M. A. (2023). Network analysis of ecological momentary assessment identifies frustration as a central node in irritability. Journal of Child Psychology and Psychiatry, 64(8), 1212–1221. doi:10.1111/jcpp.13794 |
| Tüğen | 2020 | Mental health outcomes not measured | Tüğen, L. E., Göksu, M., & Ayaz, A. B. (2020). Disruptive mood dysregulation disorder in a primary school sample. *Asian Journal of Psychiatry, 48*, 101858. doi:10.1016/j.ajp.2019.101858 |
| Vahl | 2014 | Mental health outcomes not measured | Vahl, P., Colins, O. F., Lodewijks, H. P., Markus, M. T., Doreleijers, T. A., & Vermeiren, R. R. (2014). Psychopathic-like traits in detained adolescents: Clinical usefulness of self-report. *European Child & Adolescent Psychiatry, 23*(8), 691-699. doi:10.1007/s00787-013-0497-4 |
| Van Meter | 2013 | Irritability not appropriately measured | Van Meter, A., Youngstrom, E. A., Demeter, C., & Findling, R. L. (2013). Examining the validity of cyclothymic disorder in a youth sample: replication and extension. *Journal of Abnormal Child Psychology, 41*(3), 367-378. doi:10.1007/s10802-012-9680-1 |
| Vidal-Ribas | 2018 | Sample outside age range | Vidal‐Ribas, P., Brotman, M. A., Salum, G. A., Kaiser, A., Meffert, L., Pine, D. S., Leibenluft, E., & Stringaris, A. (2018). Deficits in emotion recognition are associated with depressive symptoms in youth with disruptive mood dysregulation disorder. *Depression and Anxiety, 35*(12), 1207-1217. doi:10.1002/da.22810 |
| Vidal-Ribas | 2017 | Mental health outcomes not measured | Vidal‐Ribas, P., Pickles, A., Tibu, F., Sharp, H., & Hill, J. (2017). Sex differences in the associations between vagal reactivity and oppositional defiant disorder symptoms. *Journal of Child Psychology and Psychiatry, 58*(9), 988-997. doi:10.1111/jcpp.12750 |
| Vogel | 2021 | Data unavailable | Vogel, A. C., Tillman, R., El-Sayed, N. M., Jackson, J. J., Perlman, S. B., Barch, D. M., & Luby, J. L. (2021). Trajectory of emotion dysregulation in positive and negative affect across childhood predicts adolescent emotion dysregulation and overall functioning. *Development and Psychopathology, 33*(5), 1722-1733, doi:10.1017/s0954579421000705 |
| Wakschlag | 2024 | Mental health outcomes not measured | Wakschlag, L. S., MacNeill, L. A., Pool, L. R., Smith, J. D., Adam, H., Barch, D. M., Norton, E. S., Rogers, C. E., Ahuvia, I., Smyser, C. D., Luby, J. L., & Allen, N. B. (2024). Predictive Utility of Irritability “In Context”: Proof-of-Principle for an Early Childhood Mental Health Risk Calculator. Journal of Clinical Child and Adolescent Psychology, 53(2), 231–245. doi:10.1080/15374416.2023.2188553 |
| Waldman | 2021 | Data unavailable | Waldman, I. D., Rowe, R., Boylan, K., & Burke, J. D. (2021). External validation of a bifactor model of oppositional defiant disorder. *Molecular Psychiatry, 26*(2), 682-693. doi: 10.1038/s41380-018-0294-z |
| Weintraub | 2020 | Irritability not appropriately measured | Weintraub, M. J., Schneck, C. D., & Miklowitz, D. J. (2020). Network analysis of mood symptoms in adolescents with or at high risk for bipolar disorder. *Bipolar Disorders, 22*(2), 128-138. doi:10.1111/bdi.12870 |
| Wiggins | 2021 | Mental health outcomes not measured | Wiggins, J. L., Briggs-Gowan, M. J., Brotman, M. A., Leibenluft, E., & Wakschlag, L. S. (2021). Toward a developmental nosology for disruptive mood dysregulation disorder in early childhood. *Journal of the American Academy of Child & Adolescent Psychiatry, 60*(3), 388-397. doi:10.1016/j.jaac.2020.04.015 |
| Wiggins | 2018 | Data unavailable | Wiggins, J. L., Briggs-Gowan, M. J., Estabrook, R., Brotman, M. A., Pine, D. S., Leibenluft, E., & Wakschlag, L. S. (2018). Identifying clinically significant irritability in early childhood. *Journal of the American Academy of Child and Adolescent Psychiatry, 57*(3), 191-199. doi:10.1016/j.jaac.2017.12.008 |
| Wiggins | 2014 | Mental health outcomes not measured | Wiggins, J. L., Mitchell, C., Stringaris, A., & Leibenluft, E. (2014). Developmental trajectories of irritability and bidirectional associations with maternal depression. *Journal of the American Academy of Child & Adolescent Psychiatry, 53*(11), 1191-1205. doi:10.1016/j.jaac.2014.08.005 |
| Winters | 2018 | Data unavailable | Winters, D. E., Fukui, S., Leibenluft, E., & Hulvershorn, L. A. (2018). Improvements in irritability with open-label methylphenidate treatment in youth with comorbid attention deficit/hyperactivity disorder and disruptive mood dysregulation disorder. *Journal of Child and Adolescent Psychopharmacology, 28*(5), 298-305. doi:10.1089/cap.2017.0124 |
| Wozniak | 2005 | Irritability not appropriately measured | Wozniak, J., Biederman, J., Kwon, A., Mick, E., Faraone, S., Orlovsky, K., Schnare, L., Cargol, C., & van Grondelle, A. (2005). How cardinal are cardinal symptoms in pediatric bipolar disorder? An examination of clinical correlates. *Biological Psychiatry, 58*(7), 583-588. doi:10.1016/j.biopsych.2005.08.014 |
| Xie | 2022 | Sample outside age range | Xie, W., Bathelt, J., Fasman, A., Nelson, C. A., & Bosquet Enlow, M. (2022). Temperament and psychopathology: The “community” to which you belong matters. Child Development, 93(4), 995–1011. doi:10.1111/cdev.13742 |
| Yu | 2023 | Data duplicated or not suitable | Yu, Q., Hernandez, B., Swineford, C., Walker, N., MacNeill, L., Zhang, Y., Wakschlag, L. S., & Wiggins, J. L. (2023). Toward an optimized assessment of adolescent psychopathology risk: Multilevel environmental profiles and child irritability as predictors. JCPP Advances, 3(4), e12180-n/a. doi:10.1002/jcv2.12180 |
| Zaccaria | 2023 | Mental health outcomes not measured | Zaccaria, V., Maggi, S., Bof, A., Tofani, M., Galeoto, G., & Ardizzone, I. (2023). Validation of the Italian version of the clinician affective reactivity index (CL-ARI). Nordic Journal of Psychiatry, 77(5), 475–480. doi:10.1080/08039488.2022.2154837 |
| Zamami | 2021 | Irritability not appropriately measured | Zamami, Y., Shinzato, H., Kurihara, K., Koda, M., Nakamura, A., & Kondo, T. (2021). Prevalence and profile of depressive mixed state in patients with autism spectrum disorder. *Psychiatry Research, 300*, 113932. doi:10.1016/j.psychres.2021.113932 |
| Zhang | 2023 | Mental health outcomes not measured | Zhang, R., Aloi, J., Bajaj, S., Bashford-Largo, J., Lukoff, J., Schwartz, A., Elowsky, J., Dobbertin, M., Blair, K. S., & Blair, R. J. R. (2023). Dysfunction in differential reward-punishment responsiveness in conduct disorder relates to severity of callous-unemotional traits but not irritability. *Psychological Medicine, 53*(5), 1870-1880. doi:10.1017/s0033291721003500 |
| Zhou | 2022 | Mental health outcomes not measured | Zhou, A. M., Morales, S., Youatt, E. A., & Buss, K. A. (2022). Autonomic nervous system activity moderates associations between temperament and externalizing behaviors in early childhood. Developmental Psychobiology, 64(7), e22323-n/a. doi:10.1002/dev.22323 |

**Table S4**

*Funding Declarations for Included Studies*

| **Study** | **Funding** | **Reference** |
| --- | --- | --- |
| Aebi et al. (2016) | No external funding | Aebi, M., Barra, S., Bessler, C., Steinhausen, H. C., Walitza, S., & Plattner, B. (2016). Oppositional defiant disorder dimensions and subtypes among detained male adolescent offenders. *Journal of Child Psychology and Psychiatry*, *57*(6), 729-736. <https://doi.org/10.1111/jcpp.12473> |
| Aebi et al. (2013) | Not declared | Aebi, M., Plattner, B., Metzke, C. W., Bessler, C., & Steinhausen, H. C. (2013). Parent- and self-reported dimensions of oppositionality in youth: construct validity, concurrent validity, and the prediction of criminal outcomes in adulthood. *Journal of Child Psychology and Psychiatry*, *54*(9), 941-949. <https://doi.org/10.1111/jcpp.12039> |
| Ali et al. (2022) | Canadian Institute of Health Research, the Children’s Health Research Institute; Ontario Ministry of College and Education | Ali, O. M., Gabel, L. N., Stanton, K., Kaufman, E. A., Klein, D. N., & Hayden, E. P. (2022). Observational measures of early irritability predict children's psychopathology risk. *Development and Psychopathology*, *34*(4), 1531-1543, <https://doi.org/10.1017/S0954579421000183> |
| Althoff et al. (2014) | National Institute of Mental Health (K08MH082116) | Althoff, R. R., Kuny-Slock, A. V., Verhulst, F. C., Hudziak, J. J., & van der Ende, J. (2014). Classes of oppositional-defiant behavior: concurrent and predictive validity. *Journal of Child Psychology and Psychiatry*, *55*(10), 1162-1171. <https://doi.org/10.1111/jcpp.12233> |
| Ambrosini et al. (2013) | National Institute of Mental Health (K23MH066275-01) | Ambrosini, P. J., Bennett, D. S., & Elia, J. (2013). Attention deficit hyperactivity disorder characteristics: II. Clinical correlates of irritable mood. *Journal Affective Disorders, 145*(1), 70-76. <https://doi.org/10.1016/j.jad.2012.07.014> |
| Arana et al. (2021) | Marie Sklodowska Curie Grant (707404) | Arana, C. C., de Pauw, S. S. W., van IJzendoorn, M. H., de Maat, D. A., Kok, R., & Prinzie, P. (2021). No differential susceptibility or diathesis stress to parenting in early adolescence: Personality facets predicting behaviour problems. *Personality and Individual Differences*, *170*, 110406. <https://doi.org/10.1016/j.paid.2020.110406> |
| Barclay, Silvers, & Lee (2022) | No external funding | Barclay, M. E., Silvers, J. A., & Lee, S. S. (2022). Childhood irritability: predictive validity and mediators of adolescent psychopathology. *Research on Child and Adolescent Psychopathology*, *50*(9), 1165-1177. <https://doi.org/10.1007/s10802-022-00908-2> |
| Barker, & Salekin (2012) | National Institute of Child and Human Development (1R01 HD068437-01A1) | Barker, E. D., & Salekin, R. T. (2012). Irritable oppositional defiance and callous unemotional traits: is the association partially explained by peer victimization? *Journal of Child Psychology and Psychiatry*, *53*(11), 1167-1175. <https://doi.org/10.1111/j.1469-7610.2012.02579.x> |
| Baweja et al (2021) | National Institute of Mental Health (R01 MH083692) | Baweja, R., Waschbusch, D. A., Pelham, W. E., 3rd, Pelham, W. E., Jr., & Waxmonsky, J. G. (2021). The impact of persistent irritability on the medication treatment of paediatric attention deficit hyperactivity disorder. *Frontiers in Psychiatry*, *12*, 699687. <https://doi.org/10.3389/fpsyt.2021.699687> |
| Bell et al. (2023) | No external funding | Bell, E., Pooley, A., Tam, P., Boyce, P., Bryant, R., Porter, R., & Malhi, G. S. (2023). A novel exploration of irritability in adolescent males: A preliminary study. *Australasian Psychiatry: Bulletin of the Royal Australian and New Zealand College of Psychiatrists*, *31*(3), 258–262. <https://doi.org/10.1177/10398562221141362> |
| Benarous et al. (2020a) | Amiens University Hospital (AOL11 No. 2012- A00925-38) | Benarous, X., Bury, V., Lahaye, H., Desrosiers, L., Cohen, D., & Guilé, J. M. (2020a). Sensory processing difficulties in youths with disruptive mood dysregulation disorder. *Frontiers in Psychiatry*, *11*, 164–164. <https://doi.org/10.3389/fpsyt.2020.00164> |
| Benarous et al. (2020b) | Quebec Network on Suicide, Mood Disorders and Related Disorders (ASClin #2) | Benarous, X., Renaud, J., Breton, J. J., Cohen, D., Labelle, R., & Guilé, J.M. (2020b). Are youths with disruptive mood dysregulation disorder different from youths with major depressive disorder or persistent depressive disorder? *Journal of Affective Disorders*, *265*, 207–215. <https://doi.org/10.1016/j.jad.2020.01.020> |
| Bielas et al. (2016) | Not declared | Bielas, H., Barra, S., Skrivanek, C., Aebi, M., Steinhausen, H.C., Bessler, C., & Plattner, B. (2016). The associations of cumulative adverse childhood experiences and irritability with mental disorders in detained male adolescent offenders. *Child and Adolescent Psychiatry and Mental Health*, *10*(1), 34–34. <https://doi.org/10.1186/s13034-016-0122-7> |
| Bolhuis et al. (2017) | European Union Seventh Framework Program (602768) | Bolhuis, K., Lubke, G. H., van der Ende, J., Bartels, M., van Beijsterveldt, C. E. M., Lichtenstein, P., Larsson, H., Jaddoe, V. W. V., Kushner, S. A., Verhulst, F. C., Boomsma, D. I., & Tiemeier, H. (2017). Disentangling heterogeneity of childhood disruptive behavior problems into dimensions and subgroups. *Journal of the American Academy of Child and Adolescent Psychiatry*, *56*(8), 678–686. <https://doi.org/10.1016/j.jaac.2017.05.019> |
| Brandes et al. (2019) | Social Sciences and Humanities Research Council of Canada | Brandes, C. M., Herzhoff, K., Smack, A. J., & Tackett, J. L. (2019). The p factor and the n factor: Associations between the general factors of psychopathology and neuroticism in children. *Clinical Psychological Science*, *7*(6), 1266-1284. <https://doi.org/10.1177/2167702619859332> |
| Burke (2012) | National Institute of Mental Health (MH074148, MH42529) | Burke, J. D. (2012). An affective dimension within oppositional defiant disorder symptoms among boys: personality and psychopathology outcomes into early adulthood. *Journal of Child Psychology & Psychiatry*, *53*(11), 1176-1183. <https://doi.org/10.1111/j.1469-7610.2012.02598.x> |
| Burke et al. (2014) | National Institute of Mental Health (MH095969, R01 MH59111, 2013-JF-FX- 0058); National Institute of Drug Abuse (2RO1DA012237-11) | Burke, J. D., Boylan, K., Rowe, R., Duku, E., Stepp, S. D., Hipwell, A. E., & Waldman, I. D. (2014). Identifying the irritability dimension of ODD: Application of a modified bifactor model across five large community samples of children. *Journal of Abnormal Psychology*, *123*(4), 841-851. <https://doi.org/10.1037/a0037898> |
| Busch et al. (2023) | No external funding | Busch, J., Bleckmann, C., Schettgen, L., Krey, E., & Siefen, G. (2023). A Cross-Sectional Study of Children’s Irritability Determining the Links Between Their Ego-Resilience and Symptoms of Anxiety and Depression. *Journal of Emotional and Behavioral Disorders*, 106342662211493-. <https://doi.org/10.1177/10634266221149361> |
| Caprara et al. (2017) | No external funding | Caprara, G. V., Gerbino, M., Perinelli, E., Alessandri, G., Lenti, C., Walder, M., Preda, C. E., Brunati, E., Marchesini, G., Tiberti, A., Balottin, U., Nonini, L., De Girolamo, G., Meraviglia, C., Gianatti, D., Libera, L., Martinelli, O., Steca, P., Monzani, D., … Nobile, M. (2017). Individual differences in personality associated with aggressive behavior among adolescents referred for externalizing behavior problems. *Journal of Psychopathology and Behavioral Assessment*, *39*(4), 680–692. <https://doi.org/10.1007/s10862-017-9608-8> |
| Cardinale et al. (2021) | National Institute of Mental Health (ZIAMH002781); Bench-to-Bedside Award (479969) | Cardinale, E. M., Freitag, G. F., Brotman, M. A., Pine, D. S., Leibenluft, E., & Kircanski, K. (2021). Phasic versus tonic irritability: differential associations with attention deficit hyperactivity disorder symptoms. *Journal of the American Academy of Child and Adolescent Psychiatry*, *60*(12), 1513–1523. <https://doi.org/10.1016/j.jaac.2020.11.022> |
| Cardinale et al. (2019) | National Institute of Mental Health (15-M-0182) | Cardinale, E. M., Kircanski, K., Brooks, J., Gold, A. L., Towbin, K. E., Pine, D. S., Leibenluft, E., & Brotman, M. A. (2019). Parsing neurodevelopmental features of irritability and anxiety: Replication and validation of a latent variable approach. *Development and Psychopathology*, *31*(3), 917–929. <https://doi.org/10.1017/S095457941900035X> |
| Carter-Leno et al. (2021) | Clothworkers’ Foundation (R011217 Autism M10 2011/12); National Institute for Health Research (RP-PG-1211-20016); Senior Investigator Awards (NF-SI-0514-10073 & NF-SI-0617- 10120) | Carter Leno, V., Forth, G., Chandler, S., White, P., Yorke, I., Charman, T., Pickles, A., & Simonoff, E. (2021). Behavioural and physiological response to frustration in autistic youth: associations with irritability. *Journal of Neurodevelopmental Disorders*, *13*(1), 27–27. <https://doi.org/10.1186/s11689-021-09374-1> |
| Chad-Friedman et al. (2023) | National Institute of Mental Health (R01 MH069942); National Science Foundation Graduate Research Fellowship Program | Chad-Friedman, E., Galano, M. M., Lemay, E. P., Olino, T. M., Klein, D. N., & Dougherty, L. R. (2023). Parsing between- and within-person effects: longitudinal associations between irritability and internalizing and externalizing problems from early childhood through adolescence. *Development & Psychopathology*, *35*(3), 1371-1381. <https://doi.org/10.1017/S0954579421001267> |
| Chad-Friedman et al. (2022) | College of Behavioral and Social Sciences Dean’s Research Initiative Award; UMD Research and Scholars Award. California State University San Marcos Grant Proposal Seed Money Award; California State University San Marcos Professional Development Award. National Science Foundation Graduate Research Fellowship Program | Chad-Friedman, E., Leppert, K. A., Olino, T. M., Bufferd, S. J., & Dougherty, L. R. (2022). Affective dynamics and mean levels of preschool irritability and sadness: predictors of children's psychological functioning two years later. *Child Psychiatry and Human Development*, *53*(2), 244-255. <https://doi.org/10.1007/s10578-021-01121-w> |
| Chen et al. (2021) | Eunice Kennedy Shriver National Institute of Child Health and Human Development (R21HD093912) | Chen, H.W. B., Gardner, E. S., Clarkson, T., Eaton, N. R., Wiggins, J. L., Leibenluft, E., & Jarcho, J. M. (2022). Bullying perpetration and victimization in youth: associations with irritability and anxiety. *Child Psychiatry and Human Development*, *53*(5), 1075–1082. <https://doi.org/10.1007/s10578-021-01192-9> |
| Copeland et al. (2015) | National Institute of Mental Health (MH63970, MH63671, MH48085, MH094605); National Institute on Drug Abuse (DA/MH11301) | Copeland, W. E., Brotman, M. A., & Costello, E. J. (2015). Normative irritability in youth: developmental findings from the Great Smoky Mountains study. *Journal of the American Academy of Child and Adolescent Psychiatry*, *54*(8), 635–642. <https://doi.org/10.1016/j.jaac.2015.05.008> |
| Courbet et al. (2021) | Not declared | Courbet, O., Slama, H., Purper-Ouakil, D., Massat, I., & Villemonteix, T. (2021). Context-dependent irritability in attention deficit/hyperactivity disorder: correlates and stability of family-restricted versus cross-situational temper outbursts. *Child & Adolescent Mental Health*, *26*(2), 122-133. <https://doi.org/10.1111/camh.12399> |
| Craig et al. (2021) | Canadian Institutes of Health Research (54020, 84567, 115617) | Craig, S. G., Sierra Hernandez, C., Moretti, M. M., & Pepler, D. J. (2021). The mediational effect of affect dysregulation on the association between attachment to parents and oppositional defiant disorder symptoms in adolescents. *Child Psychiatry & Human Development*, *52*, 818-828. <https://doi.org/10.1007/s10578-020-01059-5> |
| DeGroot et al. (2024) | National Science Foundation Graduate Research Fellowship Program (NSF 16-588) | DeGroot, H., Silver, J., Klein, D. N., & Carlson, G. A. (2024). Parent and Teacher Ratings of Tonic and Phasic Irritability in a Clinical Sample. *Research on Child and Adolescent Psychopathology*, *52*(6), 891–903. <https://doi.org/10.1007/s10802-023-01151-z> |
| Déry et al. (2017) | Canadian Institutes of Health Research (82694); The Social Sciences and Humanities Research Council (37890) | Déry, M., Lapalme, M., Jagiellowicz, J., Poirier, M., Temcheff, C., & Toupin, J. (2017). Predicting depression and anxiety from oppositional defiant disorder symptoms in elementary school-age girls and boys with conduct problems. *Child Psychiatry & Human Development*, *48*, 53-62. <https://doi.org/10.1007/s10578-016-0652-5> |
| Doerfler et al. (2020) | Assumption College Faculty Grant | Doerfler, L. A., Volungis, A. M., & Connor, D. F. (2020). Co-occurrence and differentiation of oppositional defiant and mood disorders among children and adolescents. *Journal of Child and Family Studies*, *29*(9), 2568-2579. <https://doi.org/10.1007/s10826-020-01756-8> |
| Dougherty et al. (2015) | Bristol Myers Squibb, Otsuka, and Pfizer; General Clinical Research Center (M01RR10710) | Dougherty, L. R., Smith, V. C., Bufferd, S. J., Kessel, E., Carlson, G. A., & Klein, D. N. (2015). Preschool irritability predicts child psychopathology, functional impairment, and service use at age nine. *Journal of Child Psychology and Psychiatry*, *56*(9), 999-1007. <https://doi.org/10.1111/jcpp.12403> |
| Dougherty et al. (2016) | National Institute of Mental Health (R01 MH069942); The General Clinical Research Center (M01 RR10710); Glaxo Smith Kline, Bristol Myers Squibb, Pfizer, and Merck | Dougherty, L. R., Smith, V. C., Bufferd, S. J., Kessel, E. M., Carlson, G. A., & Klein, D. N. (2016). Disruptive mood dysregulation disorder at the age of 6 years and clinical and functional outcomes 3 years later. *Psychological Medicine*, *46*(5), 1103-1114. <https://doi.org/10.1017/S0033291715002809> |
| Dougherty et al. (2013) | National Institute of Mental Health (R01 MH069942); General Clinical Research Center (M01 RR10710) | Dougherty, L. R., Smith, V. C., Bufferd, S. J., Stringaris, A., Leibenluft, E., Carlson, G. A., & Klein, D. N. (2013). Preschool irritability: longitudinal associations with psychiatric disorders at age 6 and parental psychopathology. *Journal of the American Academy of Child and Adolescent Psychiatry*, *52*(12), 1304-1313. <https://doi.org/10.1016/j.jaac.2013.09.007> |
| Drabick & Gadow (2012) | National Institute of Mental Health (1K01MH073717-01A2) | Drabick, D. A., & Gadow, K. D. (2012). Deconstructing oppositional defiant disorder: clinic-based evidence for an anger/irritability phenotype. *Journal of the American Academy of Child and Adolescent Psychiatry*, *51*(4), 384-393. <https://doi.org/10.1016/j.jaac.2012.01.010> |
| Dugre & Potvin (2020) | Eunice Kennedy Shriver National Institute of Child Health and Human Development of the National Institutes of Health (R01HD36916, R01HD39135, R01HD40421) | Dugré, J. R., & Potvin, S. (2020). Developmental multi-trajectory of irritability, anxiety, and hyperactivity as psychological markers of heterogeneity in childhood aggression. *Psychological Medicine*, *52*(2), 241–250. <https://doi.org/10.1017/S0033291720001877> |
| Elvin et al., (2021) | Menzies Health Institute of Queensland | Elvin, O. M., Modecki, K. L., Finch, J., Donnolley, K., Farrell, L. J., & Waters, A. M. (2021). Joining the pieces in childhood irritability: Distinct typologies predict conduct, depressive, and anxiety symptoms. *Behaviour Research & Therapy*, *136*, 103779. <https://doi.org/10.1016/j.brat.2020.103779> |
| Elvin, Waters & Modecki (2023) | Griffith University and the Australian Research Council (DP120101678 and FT130101330). | Elvin, O. M., Waters, A. M., & Modecki, K. L. (2023). Does irritability predict attention biases toward threat among clinically anxious youth? *European Child & Adolescent Psychiatry*, *32*(8), 1435–1442. <https://doi.org/10.1007/s00787-022-01954-3> |
| Evans et al. (2021) | No external funding | Evans, S. C., Abel, M. R., Doyle, R. L., Skov, H., & Harmon, S. L. (2021). Measurement and correlates of irritability in clinically referred youth: Further examination of the Affective Reactivity Index. *Journal of Affective Disorders*, *283*, 420-429. <https://doi.org/10.1016/j.jad.2020.11.002> |
| Evans, Blossom, & Fite (2020a) | Fellowships from The American Psychological Foundation | Evans, S. C., Blossom, J. B., & Fite, P. J. (2020a). Exploring longitudinal mechanisms of irritability in children: implications for cognitive-behavioral intervention. *Behavior Therapy*, *51*(2), 238-252. <https://doi.org/10.1016/j.beth.2019.05.006> |
| Evans et al. (2020b) | John D. and Catherine T. MacArthur Foundation (83,423 and 90,231); Casey Family Programs (FY08-02200). Annie E. Casey Foundation 209.0037 and 211.0004); Norlien Foundation; The Child Health and Development Institute of Connecticut (14DCF6673AA); AIM for Mental Health | Evans, S. C., Bonadio, F. T., Bearman, S. K., Ugueto, A. M., Chorpita, B. F., & Weisz, J. R. (2020b). Assessing the irritable and defiant dimensions of youth oppositional behavior using CBCL and YSR items. *Journal of Clinical Child and Adolescent Psychology*, *49*(6), 804-819. <https://doi.org/10.1080/15374416.2019.1622119> |
| Evans et al. (2020c) | Fellowships from American Psychological Foundation Mental Health (AIM Clinical Science Fellowship, SCE) | Evans, S. C., Cooley, J. L., Blossom, J. B., Pederson, C. A., Tampke, E. C., & Fite, P. J. (2020c). Examining ODD/ADHD symptom dimensions as predictors of social, emotional, and academic trajectories in middle childhood. *Journal of Clinical Child & Adolescent Psychology*, *49*(6), 912-929. <https://doi.org/10.1080/15374416.2019.1644645> |
| Evans et al. (2023) | The Child Health and Development Institute of Connecticut (14DCF6673AA; JRW); Norlien Foundation | Evans, S. C., Corteselli, K. A., Edelman, A., Scott, H., & Weisz, J. R. (2023). Is irritability a top problem in youth mental health care? A multi-informant, multi-method Investigation. *Child Psychiatry and Human Development*, *54*(4), 1027-10421. <https://doi.org/10.1007/s10578-021-01301-8> |
| Evans et al. (2016) | University of Kansas New Faculty General Research Fund; Lillan Jacobey Baur Early Childhood Fellowship. American Psychological Foundation | Evans, S. C., Pederson, C. A., Fite, P. J., Blossom, J. B., & Cooley, J. L. (2016). Teacher-Reported irritable and defiant dimensions of oppositional defiant disorder: Social, behavioral, and academic correlates. *School Mental Health*, *8*(2), 292-304. <https://doi.org/10.1007/s12310-015-9163-y> |
| Eyre et al. (2019) | The UK Medical Research Council and Wellcome (102215/2/13/2) | Eyre, O., Hughes, R. A., Thapar, A. K., Leibenluft, E., Stringaris, A., Davey Smith, G., Stergiakouli, E., Collishaw, S., & Thapar, A. (2019). Childhood neurodevelopmental difficulties and risk of adolescent depression: the role of irritability. *Journal of Child Psychology & Psychiatry*, *60*(8), 866-874. <https://doi.org/10.1111/jcpp.13053> |
| Eyre et al., (2017) | Not declared | Eyre, O., Langley, K., Stringaris, A., Leibenluft, E., Collishaw, S., & Thapar, A. (2017). Irritability in ADHD: Associations with depression liability. *Journal of Affective Disorders*, *215*, 281-287. <https://doi.org/10.1016/j.jad.2017.03.050> |
| Ezpeleta et al. (2012) | Funding was from Spanish Ministry of Science and Innovation grant PSI2009-07542 | Ezpeleta, L., Granero, R., de la Osa, N., Penelo, E., & Domenech, J. M. (2012). Dimensions of oppositional defiant disorder in 3-year-old pre-schoolers. *Journal of Child Psychology & Psychiatry*, *53*(11), 1128-1138. <https://doi.org/10.1111/j.1469-7610.2012.02545.x> |
| Ezpeleta et al. (2019) | Spanish Ministry of Economy and Competitiveness (PSI2012‐32695, PSI2015‐ 63965‐R); Secretaria d’Universitats i Recerca, Departament d’Economia i Coneixement de la Generalitat de Catalunya, (2014 SGR 312) | Ezpeleta, L., Penelo, E., de la Osa, N., Navarro, J. B., & Trepat, E. (2019). Irritability and parenting practices as mediational variables between temperament and affective, anxiety, and oppositional defiant problems. *Aggressive Behavior*, *45*(5), 550-560. <https://doi.org/10.1002/ab.21850> |
| Ezpeleta et al. (2020a) | Spanish Ministry of Science, Innovation and Universities (PGC2018-095239- B-I00) | Ezpeleta, L., Penelo, E., de la Osa, N., Navarro, J. B., & Trepat, E. (2020). How the Affective Reactivity Index (ARI) works for teachers as informants. *Journal of Affective Disorders*, *261*, 40-48. <https://doi.org/10.1016/j.jad.2019.09.080> |
| Ezpeleta et al. (2020b) | Spanish Ministry of Science, Innovation and Universities (PGC2018-095239-B-I00) | Ezpeleta, L., Penelo, E., Navarro, J. B., de la Osa, N., & Trepat, E. (2022). Irritability, defiant and obsessive-compulsive problems development from childhood to adolescence. *Journal of Youth and Adolescence*, *51*(6), 1089–1105. <https://doi.org/10.1007/s10964-021-01528-7> |
| Ezpeleta et al. (2022) | Spanish Ministry of Science, Innovation, and Universities (PGC2018-095239-B-I00) | Ezpeleta, L., Penelo, E., Navarro, J. B., Osa, N., & Trepat, E. (2020). Transdiagnostic trajectories of irritability and oppositional, depression and anxiety problems from preschool to early adolescence. *Behaviour Research and Therapy*, *134*, 103727. <https://doi.org/10.1016/j.brat.2020.103727> |
| Farchione et al. (2007) | National Institute of Mental Health (MH60952) | Farchione, T. R., Birmaher, B., Axelson, D., Kalas, C., Monk, K., Ehmann, M., Iyengar, S., Kupfer, D., & Brent, D. (2007). Aggression, hostility, and irritability in children at risk for bipolar disorder. *Bipolar Disorders*, *9*(5), 496-503. <https://doi.org/10.1111/j.1399-5618.2007.00390.x> |
| Fernandez et al. (2015) | National Institute for Health Research. Biomedical Research Centre for Mental Health at South London; National Health Service; Foundation Trust and the Institute of Psychiatry | Fernandez de la Cruz, L., Simonoff, E., McGough, J. J., Halperin, J. M., Arnold, L. E., & Stringaris, A. (2015). Treatment of children with attention-deficit/hyperactivity disorder (ADHD) and irritability: results from the multimodal treatment study of children with ADHD (MTA). *Journal of the American Academy of Child and Adolescent Psychiatry*, *54*(1), 62-70 e3. <https://doi.org/10.1016/j.jaac.2014.10.006> |
| Filippi et al. (2020) | National Institute of Mental Health (ZIAMH002781-15, NCT00018057; National Institute of Health (U01MH093349) | Filippi, C. A., Subar, A. R., Sachs, J. F., Kircanski, K., Buzzell, G., Pagliaccio, D., Abend, R., Fox, N. A., Leibenluft, E., & Pine, D. S. (2020). Developmental pathways to social anxiety and irritability: The role of the ERN. *Development & Psychopathology*, *34*(3), 1198-1200. <https://doi.org/10.1017/S0954579419001329> |
| Gadow & Drabick (2012) | National Institute of Mental Health (1K01MH073717-01A2) | Gadow, K. D., & Drabick, D. A. (2012). Anger and irritability symptoms among youth with ODD: cross-informant versus source-exclusive syndromes. *Journal of Abnormal Child Psychology*, *40*(7), 1073-1085. <https://doi.org/10.1007/s10802-012-9637-4> |
| Galano et al. (2022) | The Blue Cross Blue Shield of Michigan Foundation | Galano, M. M., Stein, S. F., Clark, H. M., Grogan-Kaylor, A., & Graham-Bermann, S. A. (2023). Eight-year trajectories of behavior problems and resilience in children exposed to early-life intimate partner violence: The overlapping and distinct effects of individual factors, maternal characteristics, and early intervention. *Development & Psychopathology*, *35*(2), 850-862. <https://doi.org/10.1017/S0954579422000104> |
| Grabell et al. (2020) | National Institute of Mental Health (K01 MH094467, R21 MH100189, R01 MH107540, K23 MH111708, R01MH107652) | Grabell, A. S., Jones, H. M., Wilett, A. E., Bemis, L. M., Wakschlag, L. S., & Perlman, S. B. (2020). Children's facial muscular movements and risk for early psychopathology: Assessing clinical utility. *Behavior Therapy*, *51*(2), 253-267. <https://doi.org/10.1016/j.beth.2019.08.004> |
| Guzick et al. (2021) | National Institute of Mental Health (1R01MH093381, 5R01MH093402) | Guzick, A. G., Geller, D. A., Small, B. J., Murphy, T. K., Wilhelm, S., & Storch, E. A. (2021). Irritability in children and adolescents with OCD. *Behavior Therapy*, *52*(4), 883-896. <https://doi.org/10.1016/j.beth.2020.11.001> |
| Harima et al. (2022) | JSPS KAKENHI (JP20K03002) | Harima, Y., Miyawaki, D., Goto, A., Hirai, K., Sakamoto, S., Hama, H., Kadono, S., Nishiura, S., & Inoue, K. (2022). Associations Between Chronic Irritability and Sensory Processing Difficulties in Children and Adolescents. *Frontiers in Psychiatry*, *13*, 860278–860278. <https://doi.org/10.3389/fpsyt.2022.860278> |
| Hawes et al. (2020) | National Institute of Mental Health (MH40501, MH50522, MH52858) | Hawes, M. T., Carlson, G. A., Finsaas, M. C., Olino, T. M., Seely, J. R., & Klein, D. N. (2020). Dimensions of irritability in adolescents: longitudinal associations with psychopathology in adulthood. *Psychological Medicine*, *50*(16), 2759-2767. <https://doi.org/10.1017/S0033291719002903> |
| Kahle et al. (2021) | National Institutes of Mental Health (2 R01 MH 091068, U54 HD079125); The MIND Institute Intellectual and Developmental Disabilities Research Center; Health Resources and Services Administration (T32HP30037) | Kahle, S., Mukherjee, P., Dixon, J. F., Leibenluft, E., Hinshaw, S. P., & Schweitzer, J. B. (2021). Irritability predicts hyperactive/impulsive symptoms across adolescence for females. *Research on Child and Adolescent Psychopathology*, *49*(2), 185-196. <https://doi.org/10.1007/s10802-020-00723-7> |
| Kalvin et al. (2021) | National Institute of Mental Health (R01MH101514); National Institute of Child Health and Human Development (R01HD083881) | Kalvin, C. B., Gladstone, T. R., Jordan, R., Rowley, S., Marsh, C. L., Ibrahim, K., & Sukhodolsky, D. G. (2021). Assessing irritability in children with autism spectrum disorder using the affective reactivity index. *Journal of Autism and Developmental Disorders*, *51*(5), 1496-1507. <https://doi.org/10.1007/s10803-020-04627-9> |
| Kessel et al. (2021) | National Institute of Mental Health (RO1MH069942, F31MH111172) | Kessel, E. M., Frost, A., Goldstein, B. L., Black, S. R., Dougherty, L. R., Carlson, G. A., & Klein, D. N. (2021). Developmental pathways from preschool irritability to multifinality in early adolescence: the role of diurnal cortisol. *Psychological Medicine*, *51*(5), 761-769. <https://doi.org/10.1017/S0033291719003684> |
| Kessel et al., (2017) | National Institute of Mental Health (RO1MH069942, F31MH09530701) | Kessel, E. M., Kujawa, A., Dougherty, L. R., Hajcak, G., Carlson, G. A., & Klein, D. N. (2017). Neurophysiological processing of emotion in children of mothers with a history of depression: the moderating role of preschool persistent irritability. *Journal of Abnormal Child Psychology*, *45*(8), 1599-1608. <https://doi.org/10.1007/s10802-017-0272-y> |
| Kessel et al. (2016) | National Institute of Mental Health (R01 MH069942); Otsuka-BMS, Schering, Pfizer, and Merck | Kessel, E. M., Meyer, A., Hajcak, G., Dougherty, L. R., Torpey-Newman, D. C., Carlson, G. A., & Klein, D. N. (2016). Transdiagnostic factors and pathways to multifinality: The error-related negativity predicts whether preschool irritability is associated with internalizing versus externalizing symptoms at age 9. *Development & Psychopathology*, *28*(4), 913-926. <https://doi.org/10.1017/S0954579416000626> |
| Khurana et al. (2023) | No external funding | Khurana, S., Wei, M. A., Karlovich, A. R., & Evans, S. C. (2023). Irritability and Suicidality in Clinically Referred Youth: Clarifying the Link by Examining the Roles of Age and Hope. *Journal of Psychopathology and Behavioral Assessment*, *45*(3), 640–649. <https://doi.org/10.1007/s10862-023-10049-5> |
| Kishida et al. (2022) | COVID-19 Research Project in Doshisha University; JSPS KAKENHI Grant (19H01765) | Kishida, K., Tsuda, M., Takahashi, F., & Ishikawa, S. I. (2022). Irritability and mental health profiles among children and adolescents: A result of latent profile analysis. *Journal of Affective Disorders*, *300*, 76-83. <https://doi.org/10.1016/j.jad.2021.12.045> |
| Kolko et al., (2007) | National Institute of Mental Health (57727, 66371) | Kolko, D. J., Baumann, B. L., Bukstein, O. G., & Brown, E. J. (2007). Internalizing symptoms and affective reactivity in relation to the severity of aggression in clinically referred, behavior-disordered children. *Journal of Child and Family Studies*, *16*(6), 745-759. <https://doi.org/10.1007/s10826-006-9120-3> |
| Kolko & Pardini (2010) | National Institute of Mental Health Grant (MH 57727, MH 078039) | Kolko, D. J., & Pardini, D. A. (2010). ODD dimensions, ADHD, and callous-unemotional traits as predictors of treatment response in children with disruptive behavior disorders. *Journal of Abnormal Psychology*, *119*(4), 713-725. <https://doi.org/10.1037/a0020910> |
| Krieger et al. (2013) | National Council for Scientific and Technological Development; Coordenacao de Aperfeicoamento de Pessoal de Nivel Superior; Sao Paulo Research Foundation | Krieger, F. V., Polanczyk, V. G., Goodman, R., Rohde, L. A., Graeff-Martins, A. S., Salum, G., Gadelha, A., Pan, P., Stahl, D., & Stringaris, A. (2013). Dimensions of oppositionality in a Brazilian community sample: testing the DSM-5 proposal and etiological links. *Journal of the American Academy of Child and Adolescent Psychiatry*, *52*(4), 389-400.e1. <https://doi.org/10.1016/j.jaac.2013.01.004> |
| Leadbeater & Ames (2017) | Canadian Institutes of Health Research (#43275; #79917; #93533; #130500) | Leadbeater, B. J., & Ames, M. E. (2017). The longitudinal effects of oppositional defiant disorder symptoms on academic and occupational functioning in the transition to young adulthood. *Journal of Abnormal Child Psychology*, *45*(4), 749-763. <https://doi.org/10.1007/s10802-016-0190-4> |
| Lee et al. (2023) | National Institute of Mental Health (R00MH110570); Yale School of Medicine; Yale Center for Clinical Investigation; Yale School of Medicine | Lee, K. S., Lebowitz, E. R., Silverman, W. K., & Tseng, W. (2023). Transactional associations of child irritability and anxiety with parent psychological control in Taiwanese school‐aged children. *JCPP Advances*, *3*(4), e12192. <https://doi.org/10.1002/jcv2.12192> |
| Legenbauer et al. (2018) | The FORUM research funding of the Ruhr University Bochum | Legenbauer, T., Hübner, J., Pinnow, M., Ball, A., Pniewski, B., & Holtmann, M. (2018). Proper emotion recognition, dysfunctional emotion regulation: the mystery of affective dysregulation in adolescent psychiatric inpatients. *Zeitschrift für Kinder- und Jugendpsychiatrie und Psychotherapie*, *46*(1), 7–16. <https://doi.org/10.1024/1422-4917/a000479> |
| Leibenluft et al. (2006) | National Institute of Mental Health (MH 36071, MH 38916, and MH 49191); National Institute on Drug Abuse | Leibenluft, E., Cohen, P., Gorrindo, T., Brook, J. S., & Pine, D. S. (2006). Chronic versus Leibenluft, E., Cohen, P., Gorrindo, T., Brook, J. S., & Pine, D. S. (2006). Chronic versus episodic irritability in youth: a community-based, longitudinal study of clinical and diagnostic associations. *Journal of Child & Adolescent Psychopharmacology*, *16*(4), 456-466. <https://doi.org/10.1089/cap.2006.16.456> |
| Leigh et al. (2020) | Not declared | Leigh, E., Lee, A., Brown, H. M., Pisano, S., & Stringaris, A. (2020). A prospective study of rumination and irritability in youth. *Journal of Abnormal Child Psychology, 48*(12), 1581-1589. <https://doi.org/10.1007/s10802-020-00706-8> |
| Lengua (2006) | National Institute of Mental Health (R29MH57703) | Lengua, L. J. (2006). Growth in temperament and parenting as predictors of adjustment during children's transition to adolescence. *Developmental Psychology*, *42*(5), 819-832. <https://doi.org/10.1037/0012-1649.42.5.819> |
| Lengua & Kovacs (2005) | Royalties Research Fund Award from the University of Washington. | Lengua, L. J., & Kovacs, E. A. (2005). Bidirectional associations between temperament and parenting and the prediction of adjustment problems in middle childhood. *Journal of Applied Developmental Psychology*, *26*(1), 21-38. <https://doi.org/10.1016/j.appdev.2004.10.001> |
| Levy et al. (2020) | Department of Psychiatry at the Hospital for Sick Children., Toronto, Canada | Levy, T., Kronenberg, S., Crosbie, J., & Schachar, R. J. (2020). Attention-deficit/hyperactivity disorder (ADHD) symptoms and suicidality in children: The mediating role of depression, irritability, and anxiety symptoms. *Journal of Affective Disorders*, *265*, 200-206. <https://doi.org/10.1016/j.jad.2020.01.022> |
| Liu et al. (2024) | Major Projects of the National Social Science Foundation of China (Grant No. 19ZDA356) | Liu, W., Guo, X., Liu, F., & Sun, Y. (2024). The Role of Emotion Regulation Strategies in the Relationship Between Temperament and Depression in Preadolescents. *Child Psychiatry and Human Development*, *55*(2), 439–452. https://doi.org/10.1007/s10578-022-01423-7 |
| Loram et al. (2021) | National Health and Medical Research Council Career Development Fellowship (1110688); Medical Research Future Fund Investigator Grant (1194297) | Loram, G., Silk, T., Ling, M. T., Fuller-Tyszkiewicz, M., Hyde, C. S., McGillivray, J., & Sciberras, E. (2021). Associations between sleep, daytime sleepiness and functional outcomes in adolescents with ADHD [Article]. Sleep Medicine, 87, 174-182. <https://doi.org/10.1016/j.sleep.2021.08.021> |
| Maire et al. (2020) | No external funding | Maire, J., Galera, C., Bioulac, S., Bouvard, M., & Michel, G. (2020). Emotional lability and irritability have specific associations with symptomatology in children with attention deficit hyperactivity disorder. *Psychiatry Research*, *285*, 112789. <https://doi.org/10.1016/j.psychres.2020.112789> |
| Martin et al. (2017) | Not declared | Martin, S. E., Hunt, J. I., Mernick, L. R., DeMarco, M., Hunter, H. L., Coutinho, M. T., & Boekamp, J. R. (2017). Temper loss and persistent irritability in pre-schoolers: implications for diagnosing disruptive mood dysregulation disorder in early childhood. *Child Psychiatry and Human Development*, *48*(3), 498-508. <https://doi.org/10.1007/s10578-016-0676-x> |
| Mikolajewski, Taylor, Iacono (2017) | National Institute of Health (DA036216, DA05147, AA09367) | Mikolajewski, A. J., Taylor, J., & Iacono, W. G. (2017). Oppositional defiant disorder dimensions: genetic influences and risk for later psychopathology. *Journal of Child Psychology & Psychiatry*, *58*(6), 702-710. <https://doi.org/10.1111/jcpp.12683> |
| Mulraney, Melvin, & Tonge, (2014) | Not declared | Mulraney, M., Melvin, G., & Tonge, B. (2014). Brief report: can irritability act as a marker of psychopathology? *Journal of Adolescence*, *37*(4), 419-423. <https://doi.org/10.1016/j.adolescence.2014.03.005> |
| Mulraney et al. (2017) | Murdoch Children’s Research Institute; Australian National Health and Medical Research Council Early Career Fellowships in Population Health (#1037159, #1037449); Australian National Health and Medical Research Council Career Development Award (#607351) | Mulraney, M., Zendarski, N., Mensah, F., Hiscock, H., & Sciberras, E. (2017). Do early internalizing and externalizing problems predict later irritability in adolescents with attention-deficit/hyperactivity disorder? *Australian and New Zealand Journal of Psychiatry*, *51*(4), 393-402. <https://doi.org/10.1177/0004867416659365> |
| Naim et al. (2021) | No external funding | Naim, R., Smith, A., Chue, A., Grassie, H., Linke, J., Dombek, K., Shaughnessy, S., McNeil, C., Cardinale, E., Agorsor, C., Cardenas, S., Brooks, J., Subar, A. R., Jones, E. L., Do, Q. B., Pine, D. S., Leibenluft, E., Brotman, M. A., & Kircanski, K. (2021). Using ecological momentary assessment to enhance irritability phenotyping in a transdiagnostic sample of youth. *Development and psychopathology*, *33*(5), 1734-1746. <https://doi.org/10.1017/S0954579421000717> |
| Nelson et al. (2018) | American Pain Society Sharon S. Keller Chronic Pain Research Grant; Cincinnati Children’s Hospital Medical Center Place Outcomes Research Award | Nelson, S., Moorman, E., Farrell, M., & Cunningham, N. (2018). Irritability is common and is related to poorer psychosocial outcomes in youth with functional abdominal pain disorders (FAPD). *Children*, *5*(4), 52. <https://doi.org/10.3390/children5040052> |
| Pan & Yeh (2019) | Tri-Service General Hospital (TSGH-C106-103, TSGH-C107-105); Ministry of Science and Technology, Taiwan (MOST105- 2314-B-016-016-MY3) | Pan, P. Y., & Yeh, C. B. (2019). Irritability and maladaptation among children: the utility of Chinese versions of the affective reactivity index and aberrant behavior checklist-irritability subscale. *Journal of Child & Adolescent Psychopharmacology*, *29*(3), 213-219. <https://doi.org/10.1089/cap.2018.0070> |
| Perhamus & Ostrov (2021) | National Science Foundation (BCS-1450777) | Perhamus, G. R., & Ostrov, J. M. (2021). Emotions and cognitions in early childhood aggression: the role of irritability and hostile attribution biases. *Research on Child and Adolescent Psychopathology*, *49*(1), 63-75. <https://doi.org/10.1007/s10802-020-00707-7> |
| Poznanski et al. (2018) | National Institute of Health (K23MH090247, R01 MH068277) | Poznanski, B., Cornacchio, D., Coxe, S., Pincus, D. B., McMakin, D. L., & Comer, J. S. (2018). The link between anxiety severity and irritability among anxious youth: evaluating the mediating role of sleep problems. *Child Psychiatry and Human Development*, *49*(3), 352-359. <https://doi.org/10.1007/s10578-017-0769-1> |
| Rappaport et al. (2020) | National Institute of Mental Health (R01MH098055, IRP- ziamh002781, T32MH020030); National Center for Research Resources (UL1TR000058) | Rappaport, L. M., Carney, D. M., Brotman, M. A., Leibenluft, E., Pine, D. S., Roberson-Nay, R., & Hettema, J. M. (2020). A population-based twin study of childhood irritability and internalizing syndromes. *Journal of Clinical Child and Adolescent Psychology*, *49*(4), 524-534. <https://doi.org/10.1080/15374416.2018.1514612> |
| Rice et al. (2017) | The Sir Jules Thorn Charitable Trust (JTA/06); Medical Research Council (G0802200); Economic and Social Research Council (ES/J011657/1), British Academy (SG- 50591); Strutt and Harper Grant from the British Medical Association | Rice, F., Sellers, R., Hammerton, G., Eyre, O., Bevan-Jones, R., Thapar, A. K., Collishaw, S., Harold, G. T., & Thapar, A. (2017). Antecedents of new-onset major depressive disorder in children and adolescents at high familial risk. *JAMA Psychiatry*, *74*(2), 153-160. <https://doi.org/10.1001/jamapsychiatry.2016.3140> |
| Rowe et al. (2010) | National Institute of Mental Health (MH63970, MH63671, MH48085); National Institute on Drug Abuse (DA/MH11301); William T. Grant Foundation | Rowe, R., Costello, E. J., Angold, A., Copeland, W. E., & Maughan, B. (2010). Developmental pathways in oppositional defiant disorder and conduct disorder. *Journal of Abnormal Psychology*, *119*(4), 726-738. <https://doi.org/10.1037/a0020798> |
| Rubens et al. (2017) | American Psychological Foundation; National Institute of Mental Health (K23MH108603) | Rubens, S. L., Evans, S. C., Becker, S. P., Fite, P. J., & Tountas, A. M. (2017). Self-reported time in bed and sleep quality in association with internalizing and externalizing symptoms in School-Age Youth. *Child Psychiatry and Human Development*, *48*(3), 455–467. <https://doi.org/10.1007/s10578-016-0672-1> |
| Silver et al. (2021) | Not declared | Silver, J., Carlson, G. A., Olino, T. M., Perlman, G., Mackin, D., Kotov, R., & Klein, D. N. (2021). Differential outcomes of tonic and phasic irritability in adolescent girls. *Journal of Child Psychology & Psychiatry*, *62*(10), 1220-1227. <https://doi.org/10.1111/jcpp.13402> |
| Silver et al. (2024) | National Institute of Mental Health (R01 MH069942); National Science Foundation Graduate Research Fellowship Program (NSF 16-588) | Silver, J., Sorcher, L., Carlson, G. A., Dougherty, L. R., & Klein, D. N. (2024). Irritability across adolescence: Examining longitudinal trajectory, stability, and associations with psychopathology and functioning at age 18. *Journal of Affective Disorders*, *354*, 611–618. <https://doi.org/10.1016/j.jad.2024.03.079> |
| Smith et al. (2019) | National Institute on Drug Abuse (P30DA016110. R01DA027828, U01MH082830, R01MH107652) | Smith, J. D., Wakschlag, L., Krogh-Jespersen, S., Walkup, J. T., Wilson, M. N., Dishion, T. J., & Shaw, D. S. (2019). Dysregulated irritability as a window on young children's psychiatric risk: Transdiagnostic effects via the family check-up. *Development and Psychopathology*, *31*(5), 1887-1899. <https://doi.org/10.1017/S0954579419000816> |
| Sorcher et al. (2022) | National Institute of Mental Health (R01MH069942, R01MH121385, R01MH122487) | Sorcher, L. K., Goldstein, B. L., Finsaas, M. C., Carlson, G. A., Klein, D. N., & Dougherty, L. R. (2022). Preschool irritability predicts adolescent psychopathology and functional impairment: A 12-Year prospective study. *Journal of the American Academy of Child and Adolescent Psychiatry*, *61*(4), 554-564 e1. <https://doi.org/10.1016/j.jaac.2021.08.016> |
| Srinivasan et al. (2024) | Wellcome Trust (grant numbers 211163/Z/18/Z and 209196/Z/17/Z) | Srinivasan, R., Flouri, E., Lewis, G., Solmi, F., Stringaris, A., & Lewis, G. (2024). Changes in early childhood irritability and its association with depressive symptoms and self-harm during adolescence in a nationally representative United Kingdom birth cohort. *Journal of the American Academy of Child and Adolescent Psychiatry, 63*(1), 39–51. <https://doi.org/10.1016/j.jaac.2023.05.027> |
| Stoddard et al. (2017) | The Intramural Program of the National Institute of Mental Health | Stoddard, J., Tseng, W. L., Kim, P., Chen, G., Yi, J., Donahue, L., Brotman, M. A., Towbin, K. E., Pine, D. S., & Leibenluft, E. (2017). Association of irritability and anxiety with the neural mechanisms of implicit face emotion processing in youths with psychopathology. *JAMA Psychiatry*, *74*(1), 95-103. <https://doi.org/10.1001/jamapsychiatry.2016.3282> |
| Stringaris & Goodman (2009b) | British Department of Health. | Stringaris, A., & Goodman, R. (2009a). Three dimensions of oppositionality in youth. *Journal of Child Psychology & Psychiatry*, *50*(3), 216-223. <https://doi.org/10.1111/j.1469-7610.2008.01989.x> |
| Stringaris & Goodman (2009a) | Not declared | Stringaris, A., & Goodman, R. (2009b). Longitudinal outcome of youth oppositionality: irritable, headstrong, and hurtful behaviors have distinctive predictions. *Journal of the American Academy of Child and Adolescent Psychiatry*, *48*(4), 404-412. <https://doi.org/10.1097/CHI.0b013e3181984f30> |
| Stringaris et al. (2012) | No external funding | Stringaris, A., Zavos, H., Leibenluft, E., Maughan, B., & Eley, T. C. (2012). Adolescent irritability: phenotypic associations and genetic links with depressed mood. *The American Journal of Psychiatry*, *169*(1), 47-54. <https://doi.org/10.1176/appi.ajp.2011.10101549> |
| Theriault et al. (2018) | Not declared | Theriault, M. G., Becue, J. C., Lesperance, P., Chouinard, S., Rouleau, G. A., & Richer, F. (2018). Oppositional behavior and longitudinal predictions of early adulthood mental health problems in chronic tic disorders. *Psychiatry Research*, *266*, 301-308. <https://doi.org/10.1016/j.psychres.2018.03.026> |
| Ucar & Vural (2018) | Not declared | Ucar, H. N., & Vural, A. P. (2018). Irritability and parenting styles in adolescents with attention-deficit/hyperactivity disorder: A controlled study. *Journal of Psychosocial Nursing and Mental Health Services*, *56*(9), 33-43. <https://doi.org/10.3928/02793695-20180412-02> |
| Valencia et al. (2021). | Spanish Ministry of Science, Innovation and Universities (PGC2018-095239-B-I00) | Valencia, F., Penelo, E., de la Osa, N., Navarro, J. B., & Ezpeleta, L. (2021). Prospective association of parental and child internalizing symptoms: Mediation of parenting practices and irritability. *British Journal of Developmental Psychology*, *39*(3), 363-379. <https://doi.org/10.1111/bjdp.12367> |
| Vogel et al. (2019) | National Institute of Mental Health (R01 MH064769, MH090786, T32 MH100019) | Vogel, A. C., Jackson, J. J., Barch, D. M., Tillman, R., & Luby, J. L. (2019). Excitability and irritability in pre-schoolers predict later psychopathology: The importance of positive and negative emotion dysregulation. *Development & Psychopathology*, *31*(3), 1067-1083. <https://doi.org/10.1017/S0954579419000609> |
| Wakschlag et al. (2015) | National Institute of Mental Health (R01MH082830, UO1MH090301, 2U01MH082830, K01MH094467); Walden and Jean Young Shaw Foundation. | Wakschlag, L. S., Estabrook, R., Petitclerc, A., Henry, D., Burns, J. L., Perlman, S. B., Voss, J. L., Pine, D. S., Leibenluft, E., & Briggs-Gowan, M. L. (2015). Clinical implications of a dimensional approach: the normal: abnormal spectrum of early irritability. *Journal of the American Academy of Child and Adolescent Psychiatry*, *54*(8), 626-634. <https://doi.org/10.1016/j.jaac.2015.05.016> |
| Wakschlag et al. (2020) | National Institute of Mental Health (R01MH107652, R01 MH107540) | Wakschlag, L. S., Krogh-Jespersen, S., Estabrook, R., Hlutkowsky, C. O., Anderson, E. L., Burns, J., Briggs-Gowan, M. J., Petitclerc, A., & Perlman, S. B. (2020). The early childhood irritability-related impairment interview (E-CRI): A novel method for assessing young children's developmentally impairing irritability. *Behavior Therapy*, *51*(2), 294-309. <https://doi.org/10.1016/j.beth.2019.07.008> |
| Wang et al. (2023) | National Natural Science Foundation of China (32171064) | Wang, F., Wang, M., Wang, X., & Zhao, J. (2023). Child- and family-level factors as predictors of Chinese children’s generalized anxiety disorder symptoms in middle childhood. *Current Psychology (New Brunswick, N.J.)*, *42*(29), 25061–25074. https://doi.org/10.1007/s12144-022-03583-0 |
| Waschbusch et al. (2020) | Nova Scotia Health Research Foundation (304e); Social Sciences and Humanities Research Council of Canada (839- 2000-1061, 410-2004-1272) | Waschbusch, D. A., Baweja, R., Babinski, D. E., Mayes, S. D., & Waxmonsky, J. G. (2020). Irritability and limited prosocial emotions/callous-unemotional traits in elementary-school-age children. *Behavior Therapy*, *51*(2), 223-237. <https://doi.org/10.1016/j.beth.2019.06.007> |
| Waxmonsky et al. (2022) | Pennsylvania Department of Health using Tobacco CURE Funds; Children’s Miracle Network; National Institute of Mental Health (T32 -MH18921) | Waxmonsky, J. G., Fosco, W., Waschbusch, D., Babinski, D., Baweja, R., Pegg, S., Cao, V., Shroff, D., & Kujawa, A. (2022). The impact of irritability and callous unemotional traits on reward positivity in youth with ADHD and conduct problems. *Research on Child and Adolescent Psychopathology*, *50*(8), 1027-1040. <https://doi.org/10.1007/s10802-022-00901-9> |
| Waxmonsky et al. (2017) | National Institutes of Health (RO1 HL063772, MO1 RR10732, CO6 RRO16499) | Waxmonsky, J. G., Mayes, S. D., Calhoun, S. L., Fernandez-Mendoza, J., Waschbusch, D. A., Bendixsen, B. H., & Bixler, E. O. (2017). The association between disruptive mood dysregulation disorder symptoms and sleep problems in children with and without ADHD. *Sleep Medicine*, *37*, 180-186. <https://doi.org/10.1016/j.sleep.2017.02.006> |
| Whelan et al. (2015) | National Institutes of Health (R01HD068437, ES/ J500021/1 W85058B); National Institute of Mental Health Division of Intramural Research Programs; Wellcome Trust; National Institute for Health Research; Department of Health UK | Whelan, Y. M., Leibenluft, E., Stringaris, A., & Barker, E. D. (2015). Pathways from maternal depressive symptoms to adolescent depressive symptoms: the unique contribution of irritability symptoms. *Journal of Child Psychology & Psychiatry, 56*(10), 1092-1100. <https://doi.org/10.1111/jcpp.12395> |
| Wiggins et al. (2023) | National Institute of Mental Health (2U01MH082830, R01MH082830) | Wiggins, J. L., Ureña Rosario, A., MacNeill, L. A., Krogh‐Jespersen, S., Briggs‐Gowan, M., Smith, J. D., & Wakschlag, L. S. (2023). Prevalence, stability, and predictive utility of the Multidimensional Assessment of Preschoolers Scales clinically optimized irritability score: Pragmatic early assessment of mental disorder risk. *International Journal of Methods in Psychiatric Research*, *32*(S1), e1991–e1991. https://doi.org/10.1002/mpr.1991 |
| Wilson et al. (2022) | National Institute of Mental Health (K23 MH090247, F31 112296); Andrew Kukes Foundation for Social Anxiety | Wilson, M. K., Cornacchio, D., Brotman, M. A., & Comer, J. S. (2022). Measuring irritability in early childhood: A psychometric evaluation of the affective reactivity index in a clinical sample of 3- to 8-year-old children. *Assessment*, *29*(7), 1473-1481. <https://doi.org/10.1177/10731911211020078> |
| Zendarski et al. (2023) | Open Access funding enabled and organized by CAUL and its Member Institution | Zendarski, N., Galligan, R., Coghill, D., Payne, J. M., De Luca, C. R., & Mulraney, M. (2023). The Associations between Child Irritability, Parental Distress, Parental Irritability and Family Functioning in Children Accessing Mental Health Services. *Journal of Child and Family Studies*, *32*(1), 288–300. <https://doi.org/10.1007/s10826-022-02390-2> |
| Zhou, Lengua, & Wang, (2009) | American Psychological Association Dissertation Award; National Institute of Mental Health | Zhou, Q., Lengua, L. J., & Wang, Y. (2009). The relations of temperament reactivity and effortful control to children's adjustment problems in China and the United States. *Developmental Psychology*, *45*(3), 724-739. <https://doi.org/10.1037/a0013776> |
| Zik et al. (2022) | Intramural Research Program of the National Institute of Mental Health (NIMH; ZIAMH002786, R25MH125758, K23MH113731); National Institute of Alcohol Abuse and Alcoholism (NIAAA; K23AA026635) | Zik, J., Deveney, C., Ellingson, J., Haller, S. P., Kircanski, K., Cardinale, E., Brotman, M. A., & Stoddard, J. (2022). Irritability interrelations with anger and aggression, and the effects of the informant. *Journal of the American Academy of Child and Adolescent Psychiatry*, *59*(10), S196-S196. <https://doi.org/10.1016/j.jaac.2020.08.221> |

**Table S5**

*Search Strategies and Results for Each Database*

| **Database** | **Fields** | **Search period** | **Number of Studies** | **Search period** | **Number of Studies** |
| --- | --- | --- | --- | --- | --- |
| Scopus | Row 1: All fields  Row 2: All fields  Row 3: All fields | To 2022 | 34 | June 2022 to 2024 | 3 |
| PsycARTICLES | Row 1: All fields  Row 2: All fields  Row 3: All fields | To 2022 | 270 | June 2022 to 2024 | 41 |
| PsycINFO | Row 1: Abstract  Row 2: Abstract  Row 3: Any field | To 2022 | 2987 | June 2022 to 2024 | 402 |
| Web of Science | Row 1: Abstract  Row 2: Abstract  Row 3: Any field | 1965-01-01 to 2022-06-30 | 2529 | 2022-06-30 to 2024-06-02 | 580 |
| PubMed/MEDLINE | Row 1: Title/Abstract  Row 2: Title/Abstract  Row 3: All Fields | To 2022 | 2848 | June 2022 to 2024 | 533 |

The search terms used for each database were:

Row 1*:* irritability [OR temperament OR negative affectivity] AND

Row 2: child [OR preschool OR early years OR adolescence] AND

Row 3: psychological [OR mental health OR psychopathology OR psychological disorder OR depression OR anxiety OR oppositional defiant disorder OR conduct disorder OR ADHD OR suicide OR autism OR internalising OR externalising OR substance OR abuse OR obsessive compulsive disorder OR eating disorder OR social functioning OR emotional functioning].

**Figure S6a**

*Model 1 Concurrent Studies Forrest Plot*

**
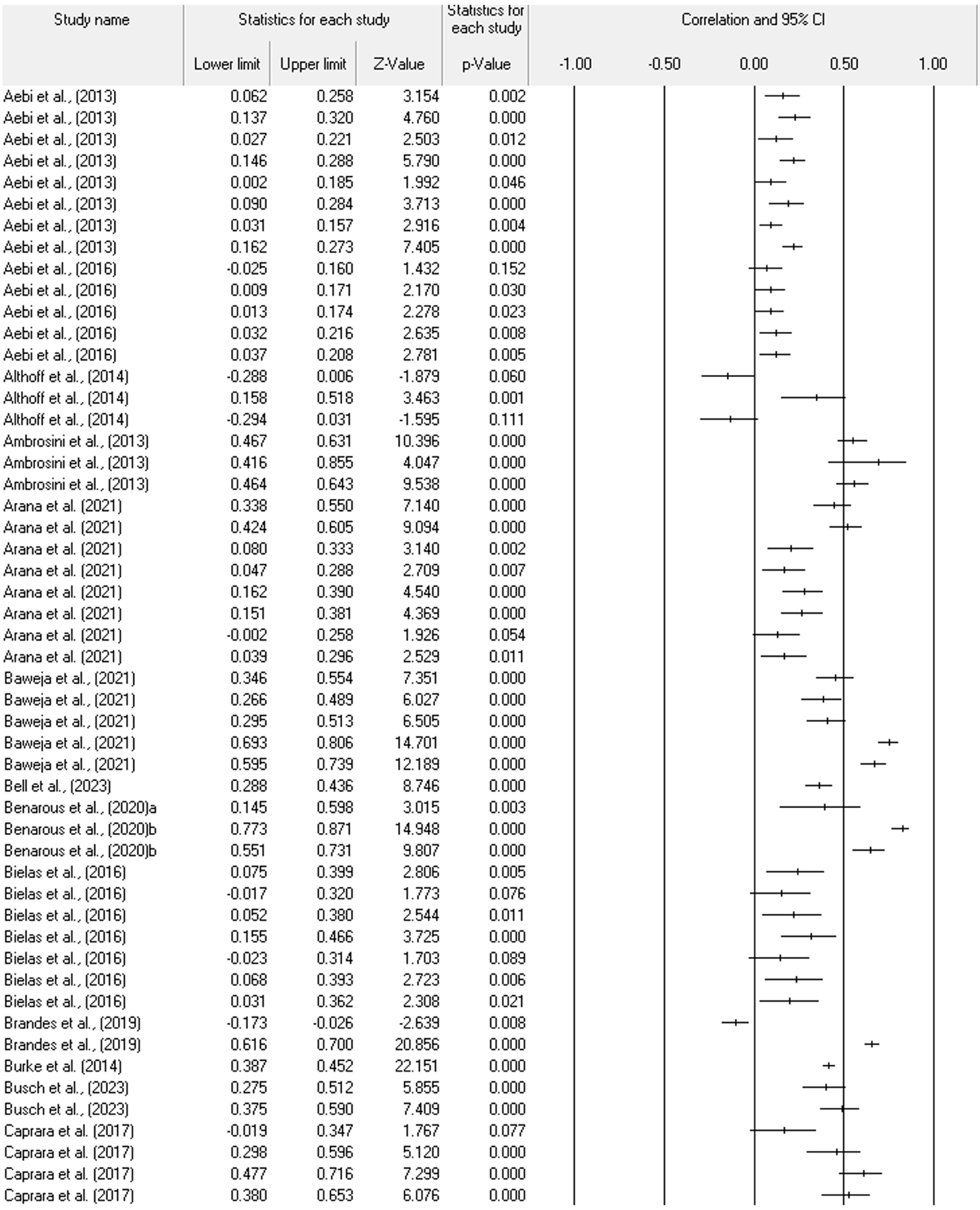
**

**
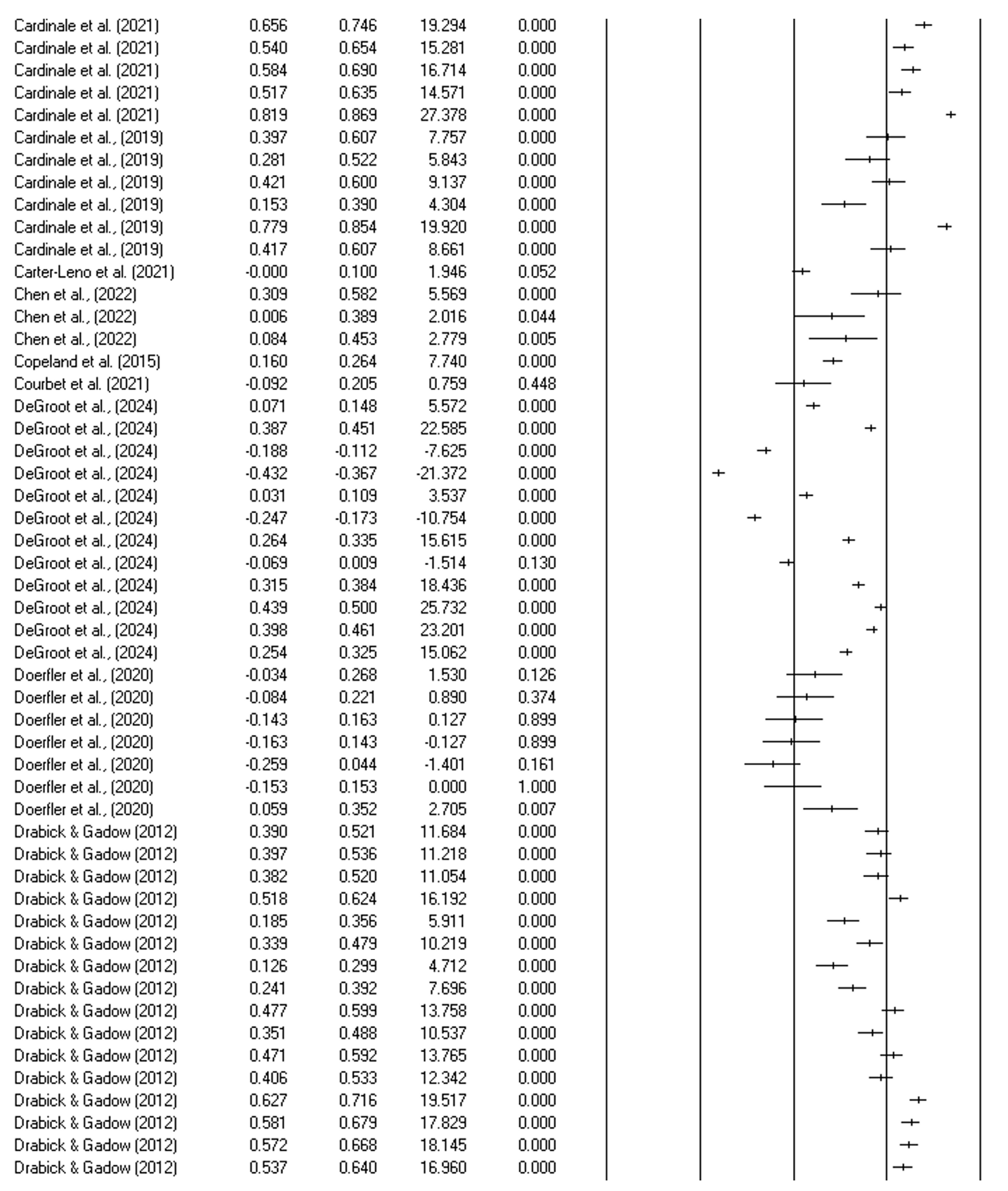
**

**
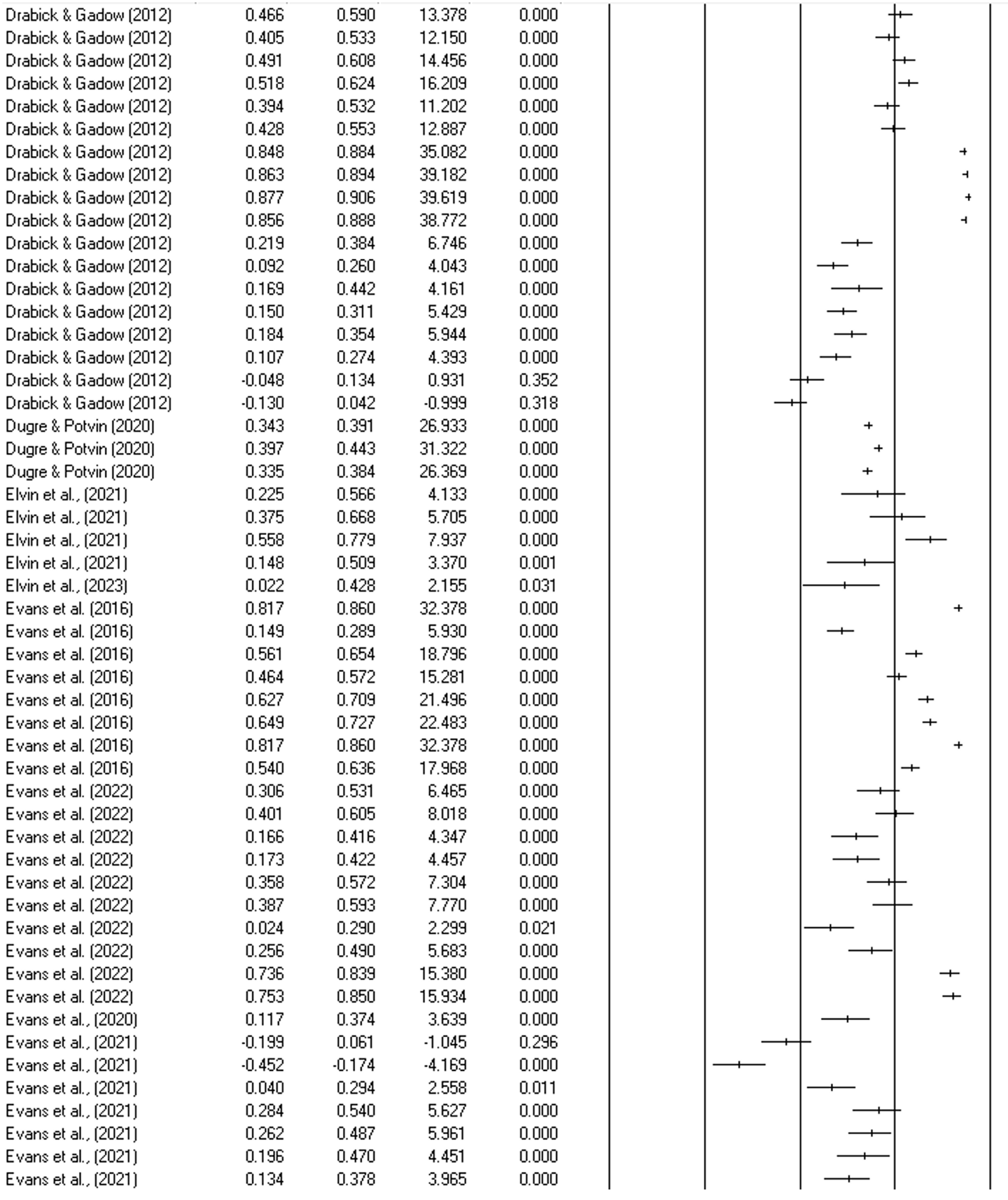
**

**
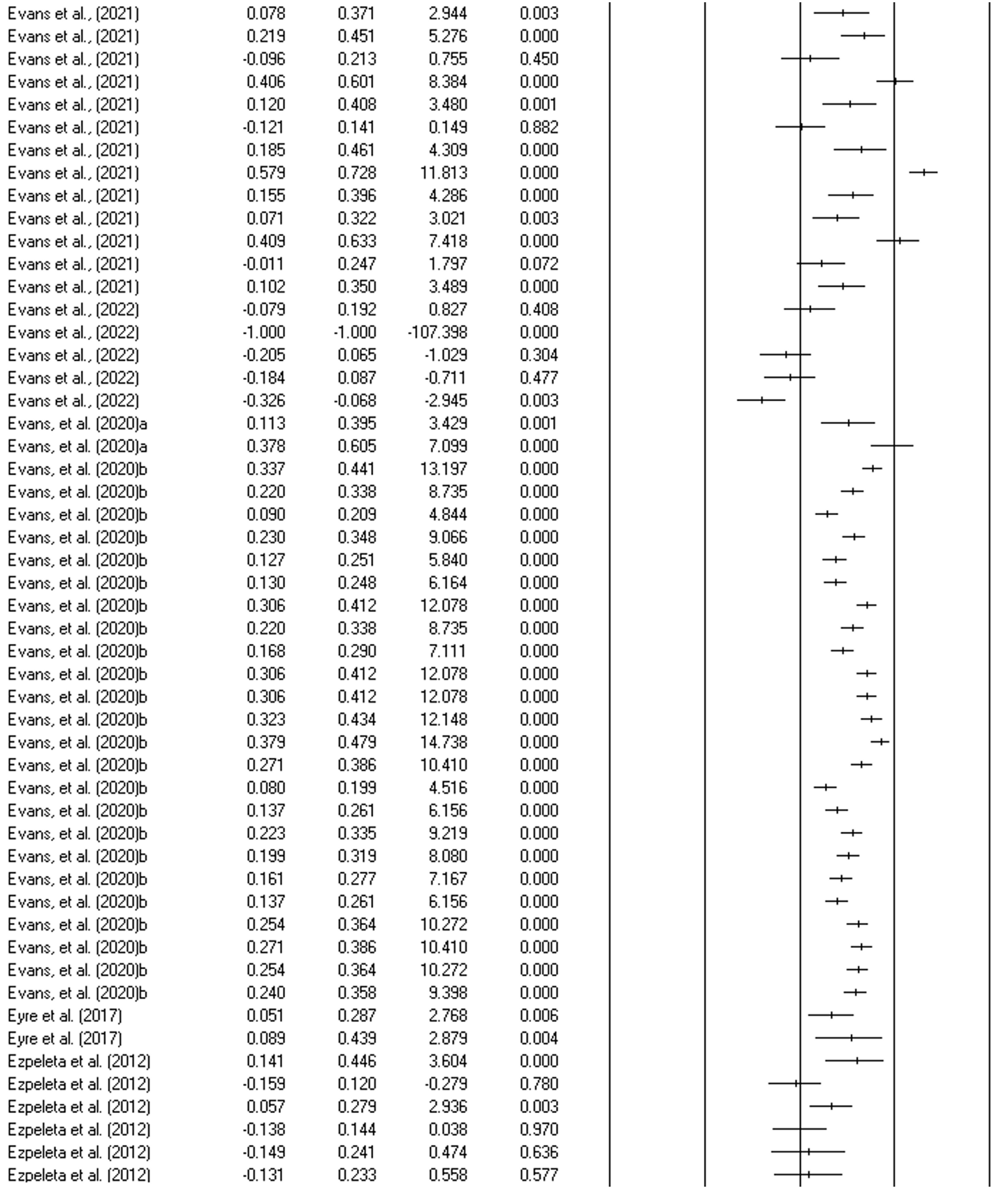
**

**
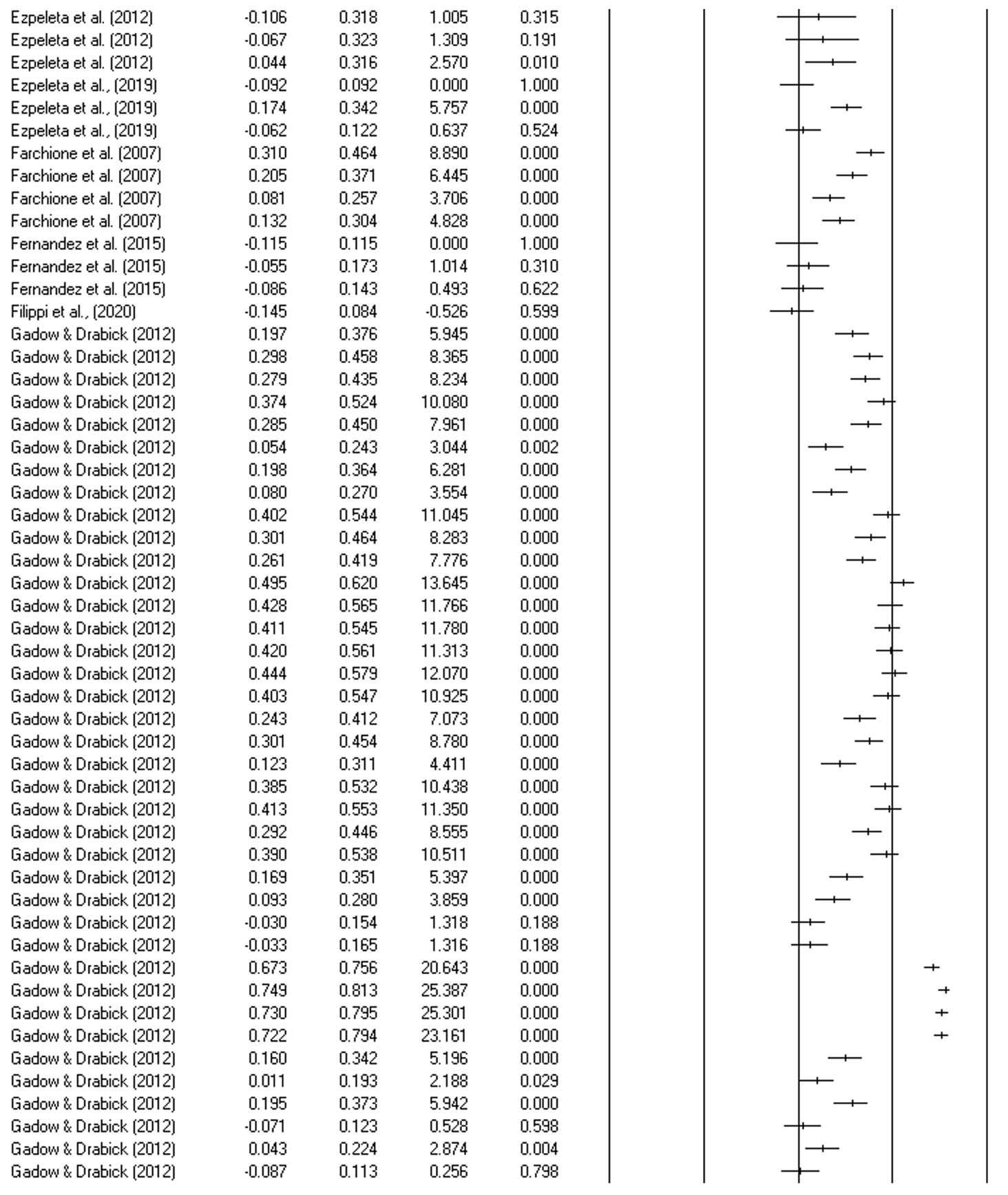
**


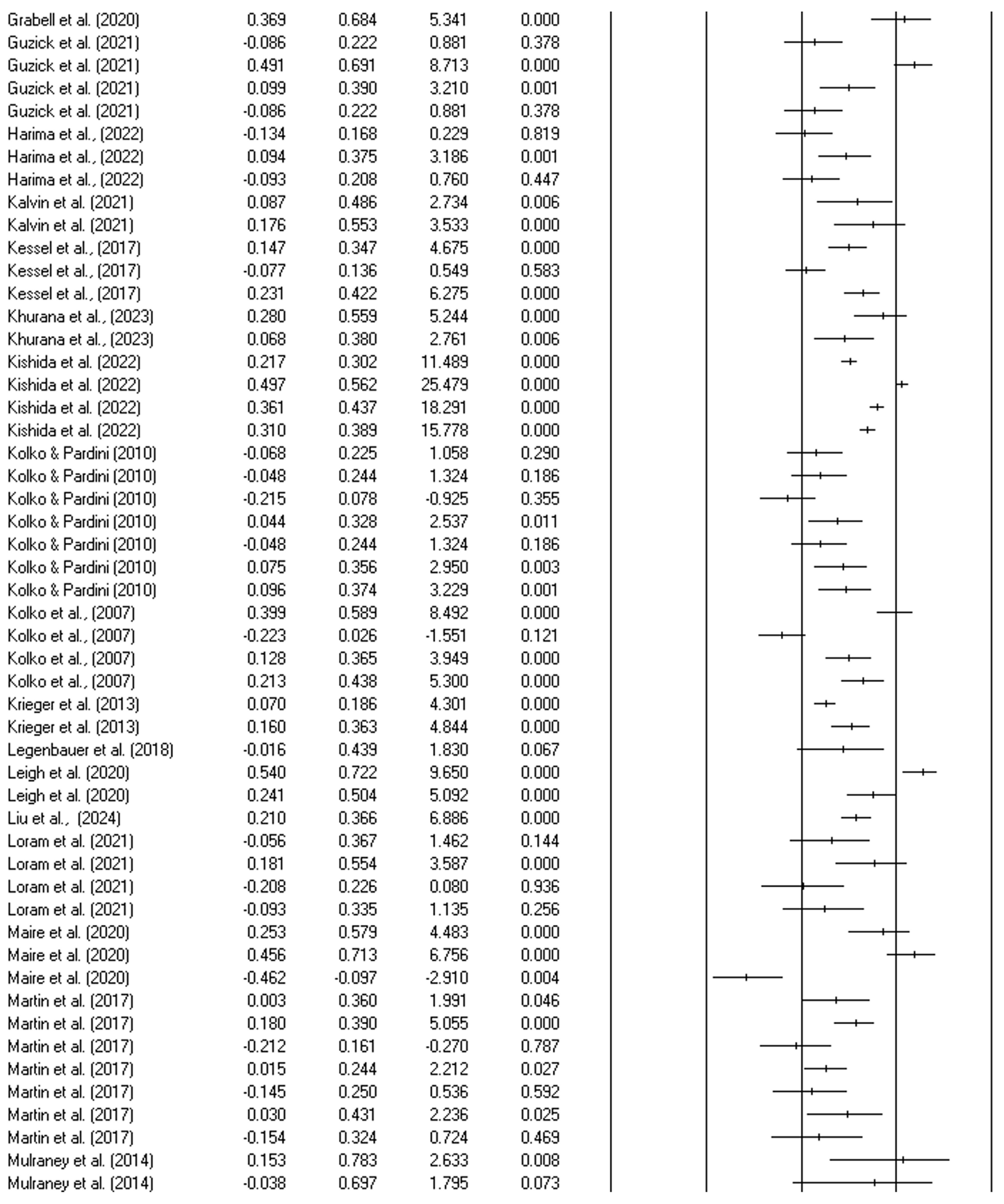


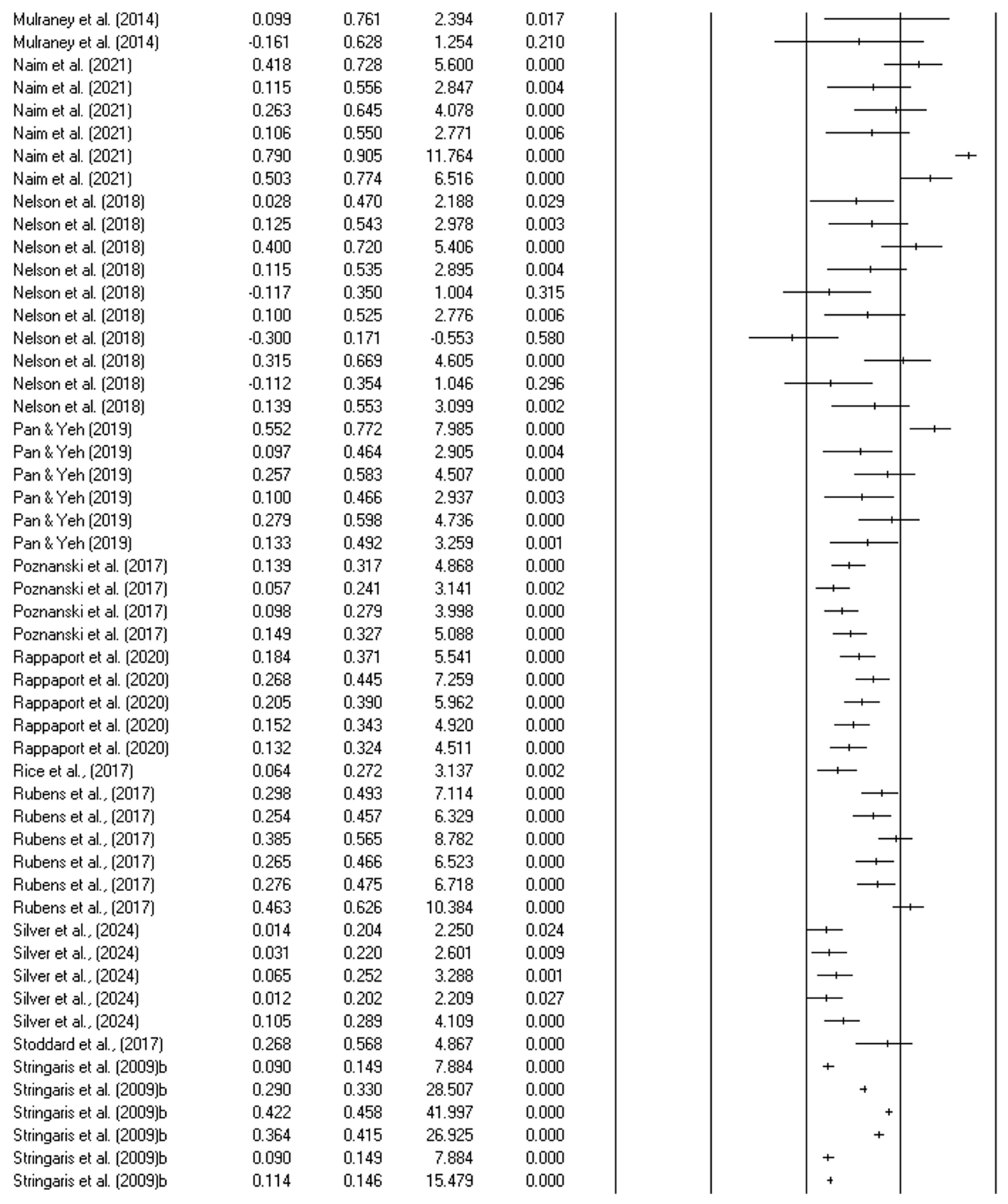


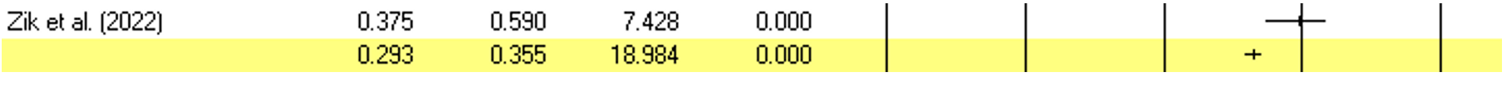

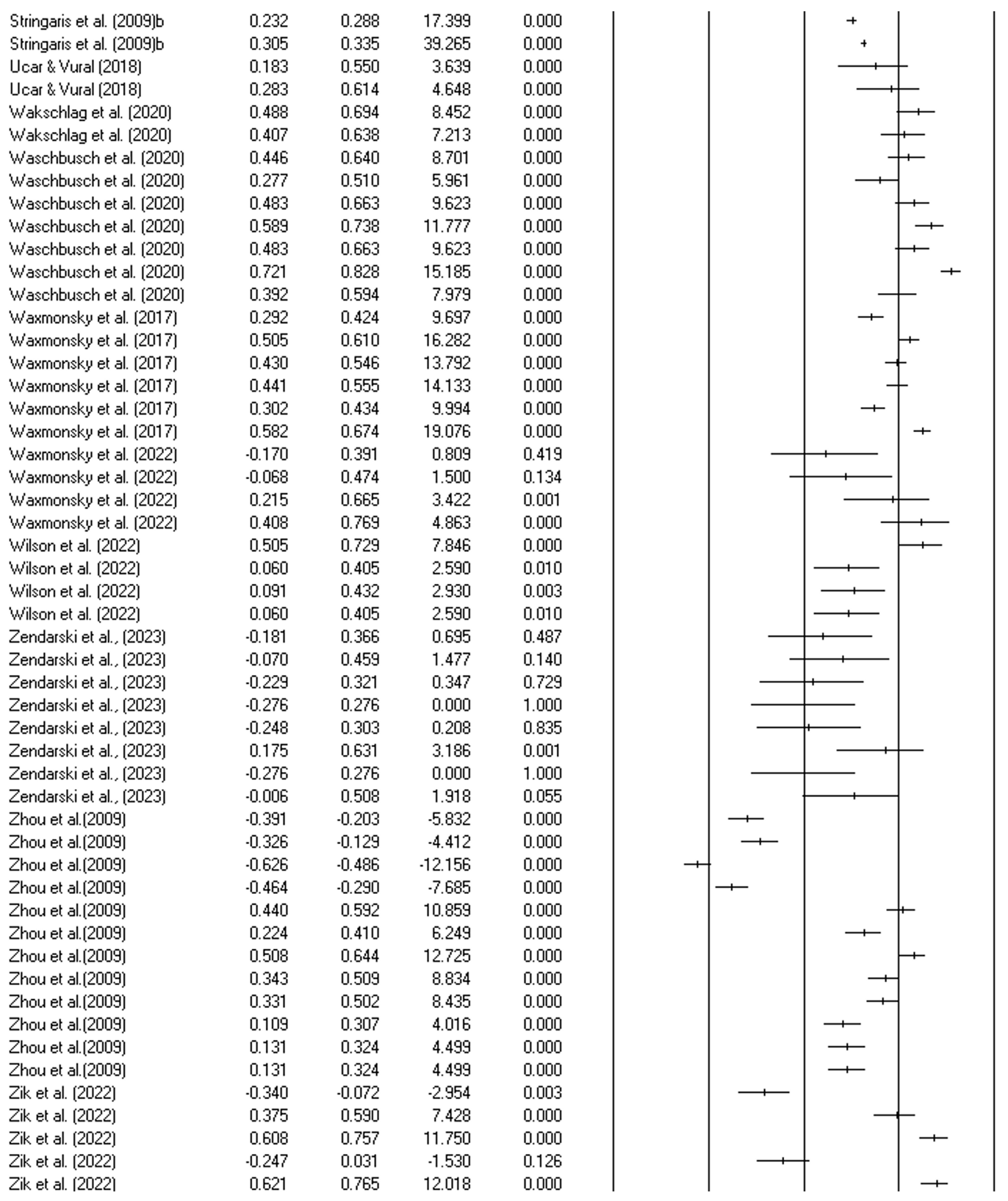


**Figure S6b**

*Model 1 Concurrent Studies Funnel Plot*


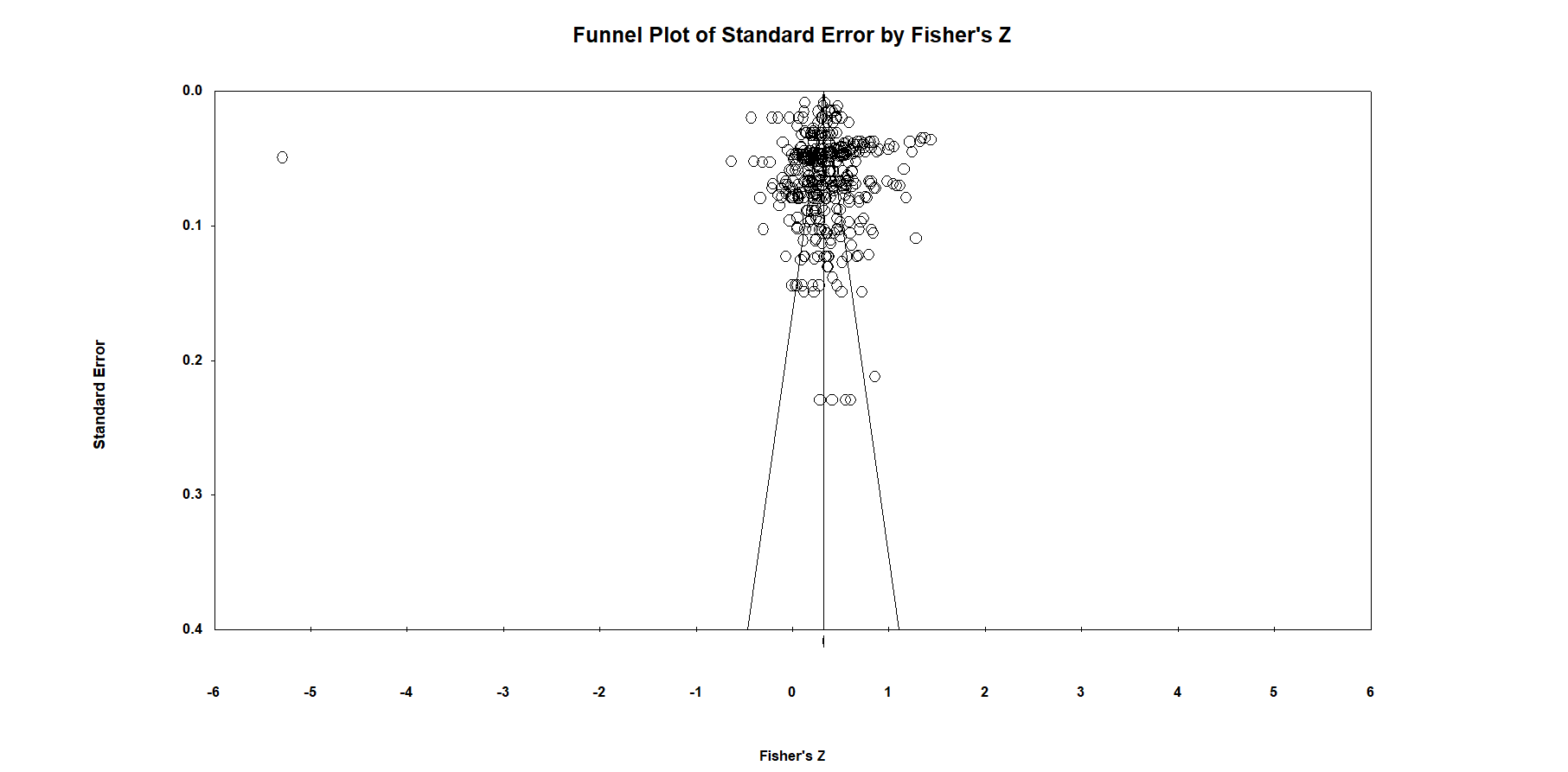


**Figure S6c**

*Model 1 Concurrent Studies Moderation Study Quality Scatterplot*


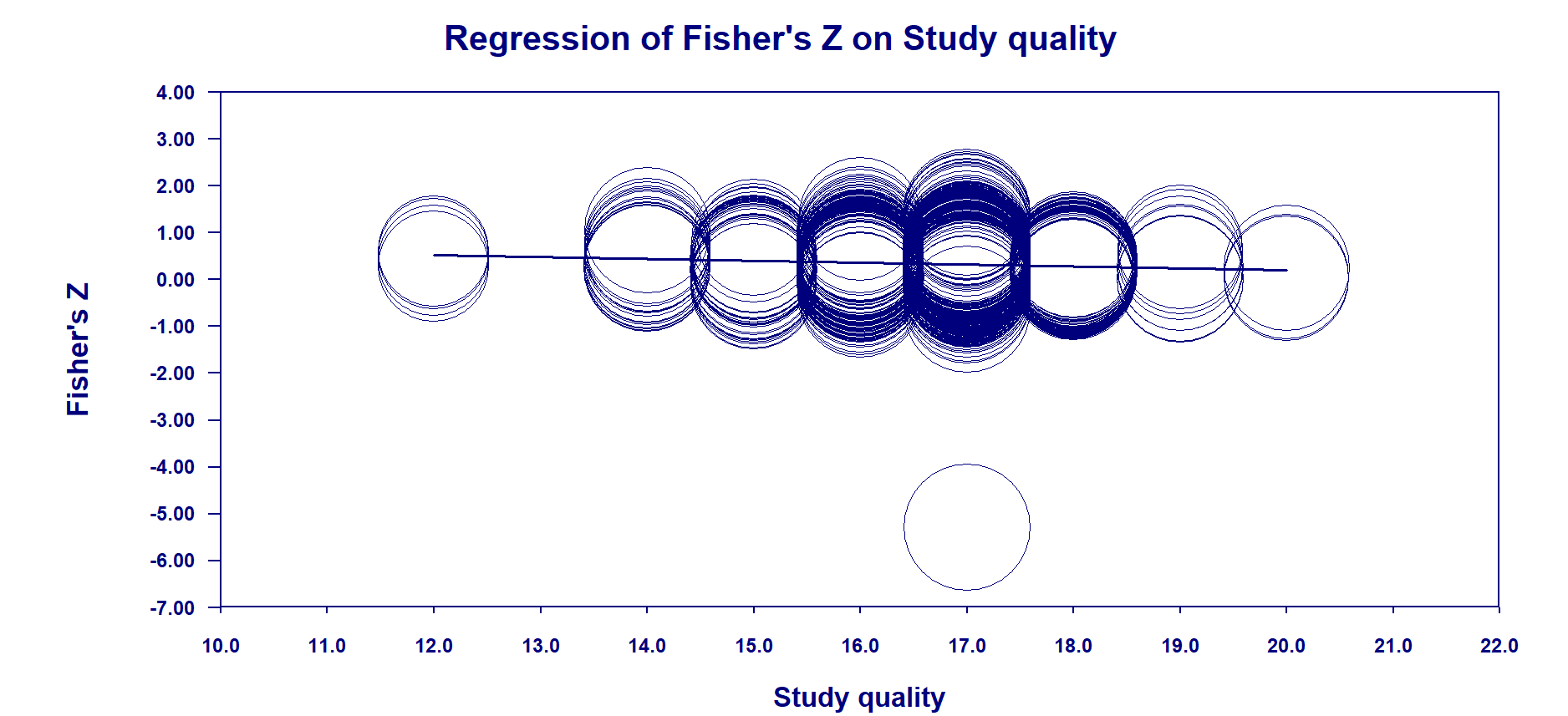


**Figure S6d**

*Model 1 Concurrent Studies Moderation Externalising and Internalising Symptoms Scatterplot*


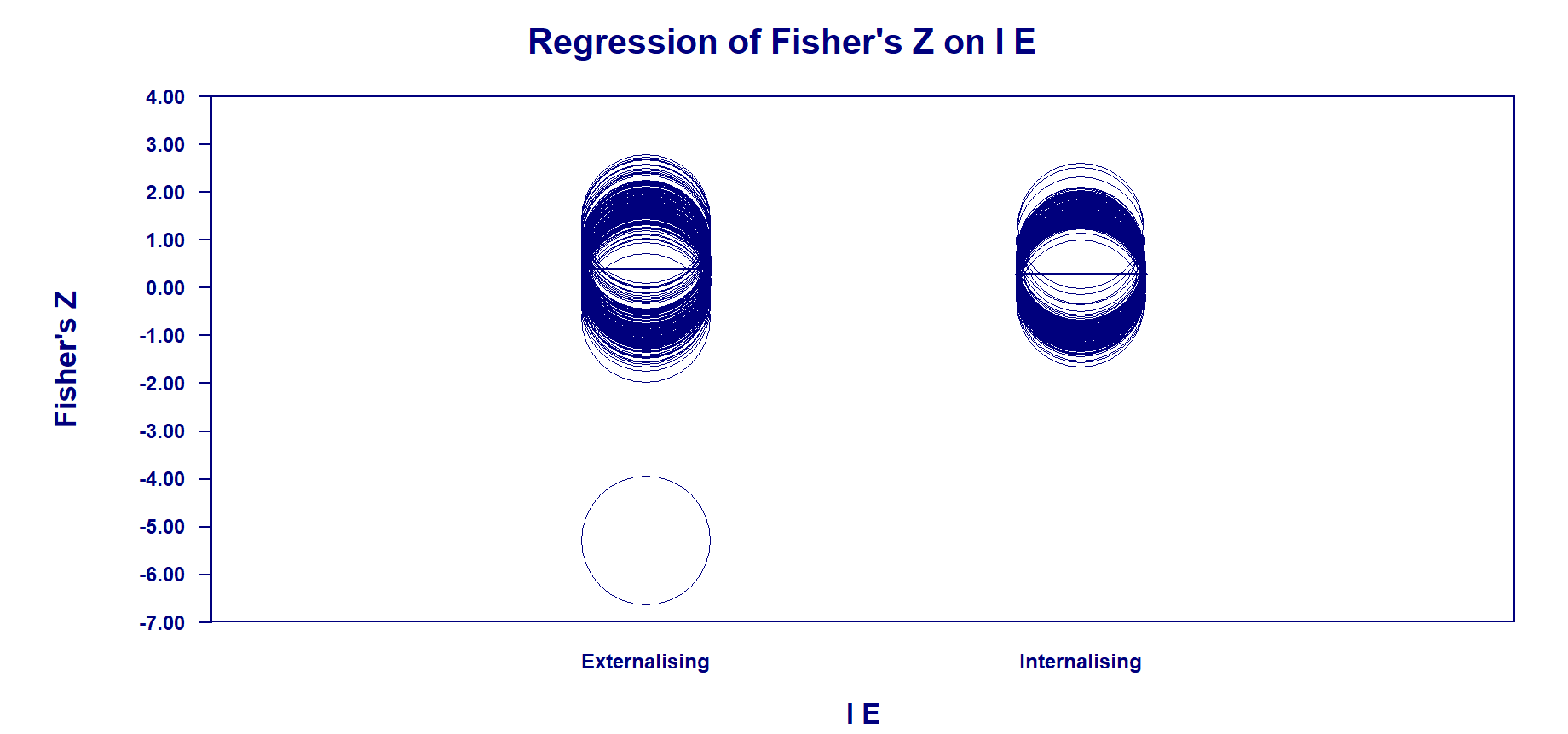


**Figure S6e**

*Model 1 Concurrent Studies Moderation Diagnostic Domains Scatterplot*


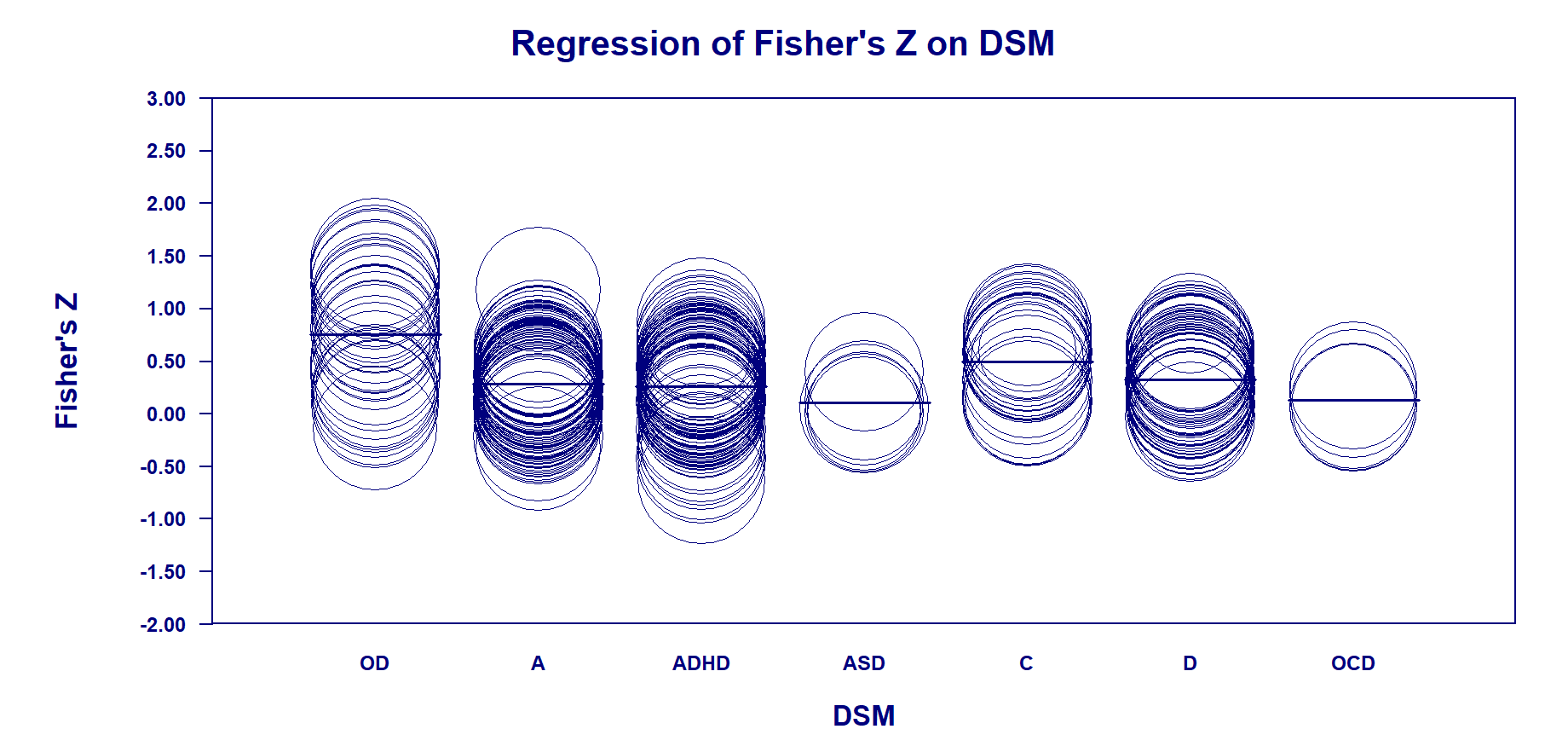


**Figure S6f**

*Model 2 Longitudinal Association Preschool to Middle Childhood Forrest Plot*

**
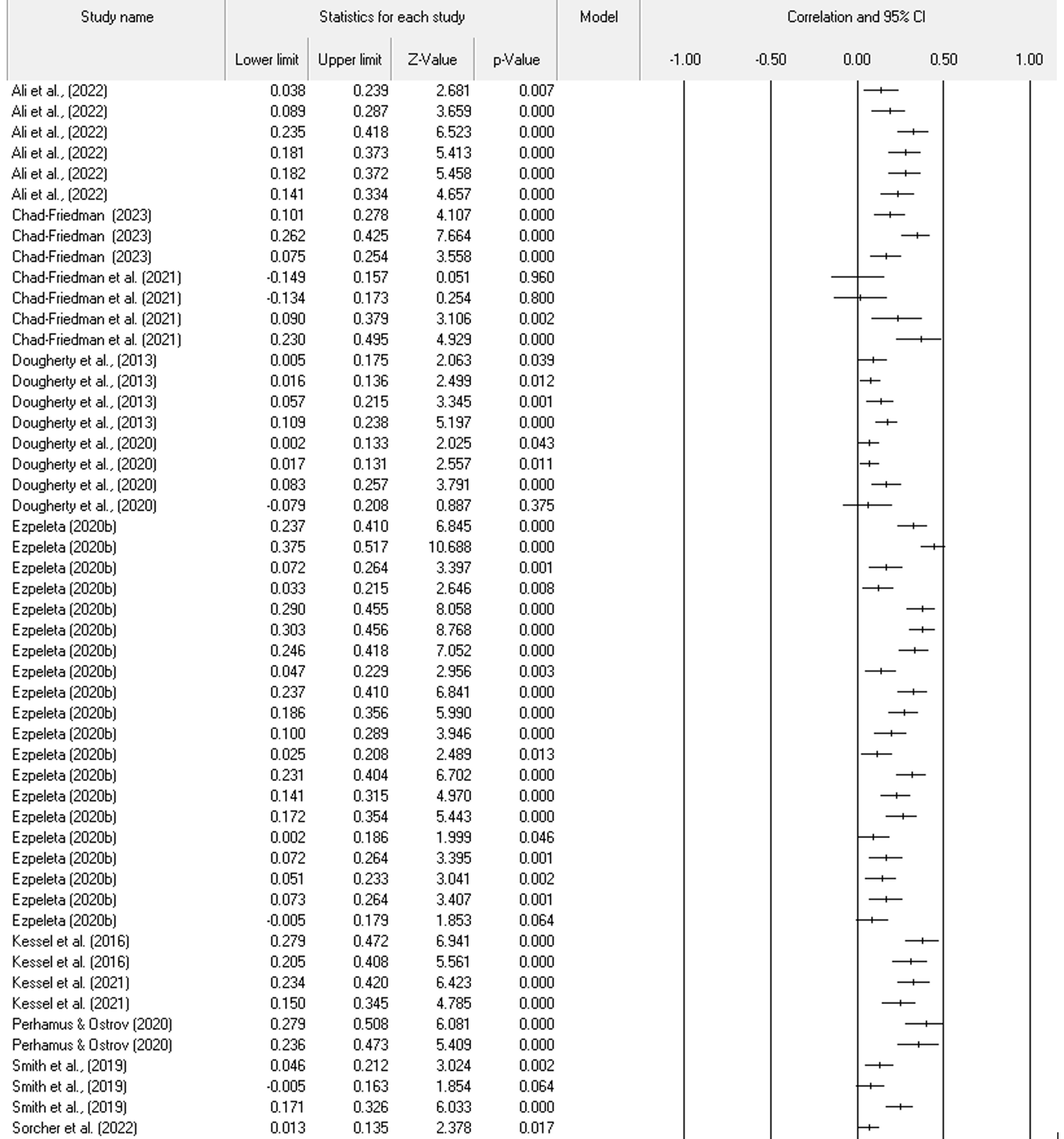
**
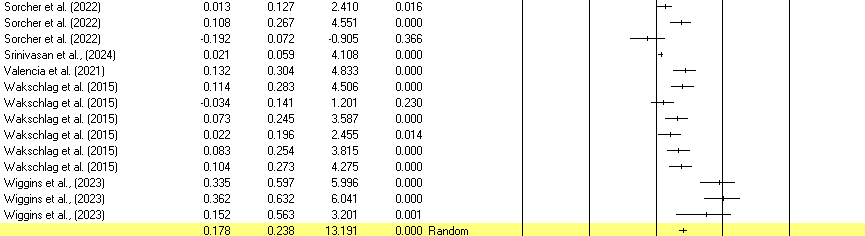


**Figure S6g**

*Model 2 Longitudinal Association: Preschool to Middle Childhood Funnel Plot*

**
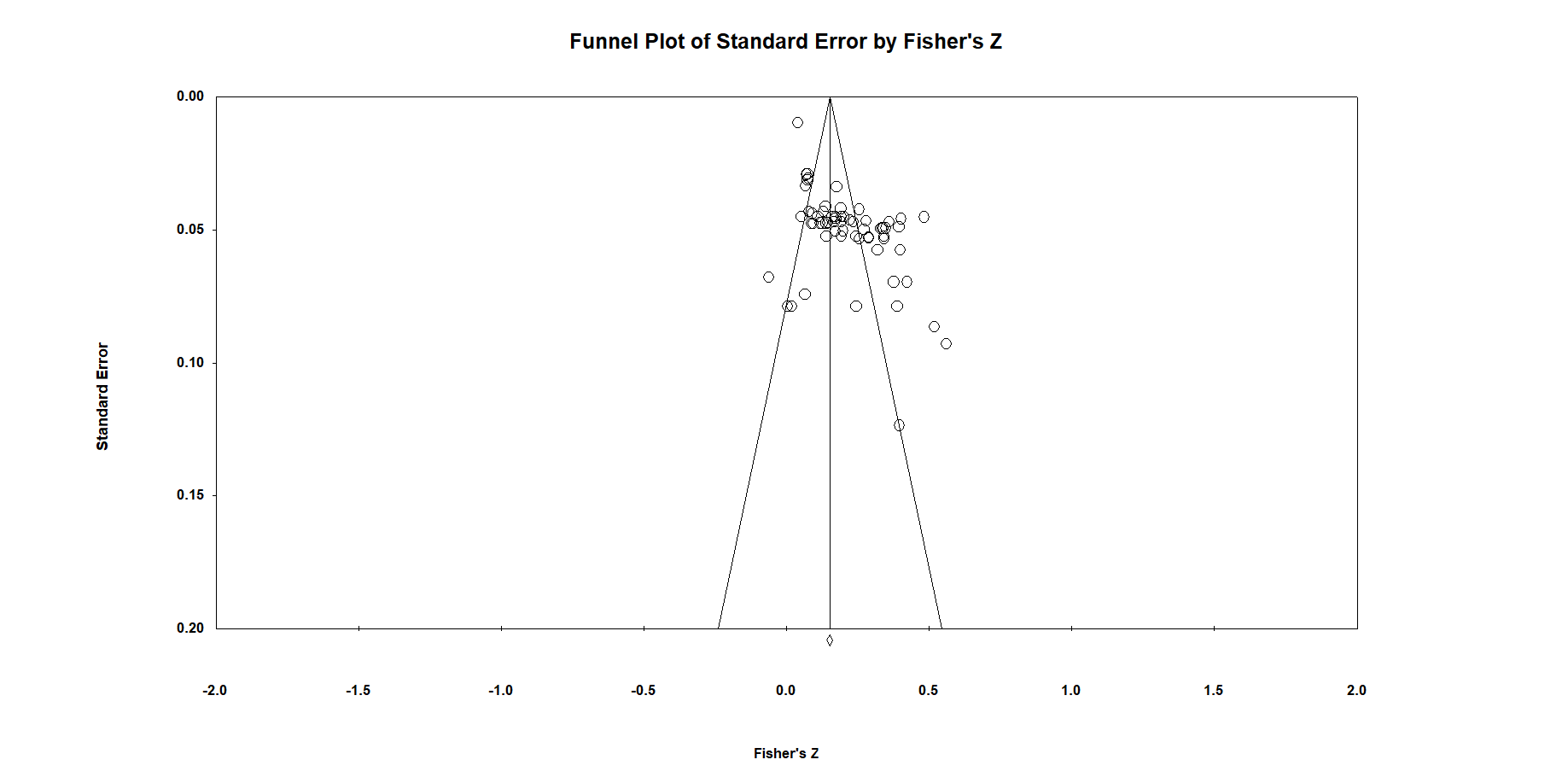
**

**Figure S6h**

*Model 2 Longitudinal Association: Preschool to Middle Childhood Moderation Diagnostic Domains Scatterplot*


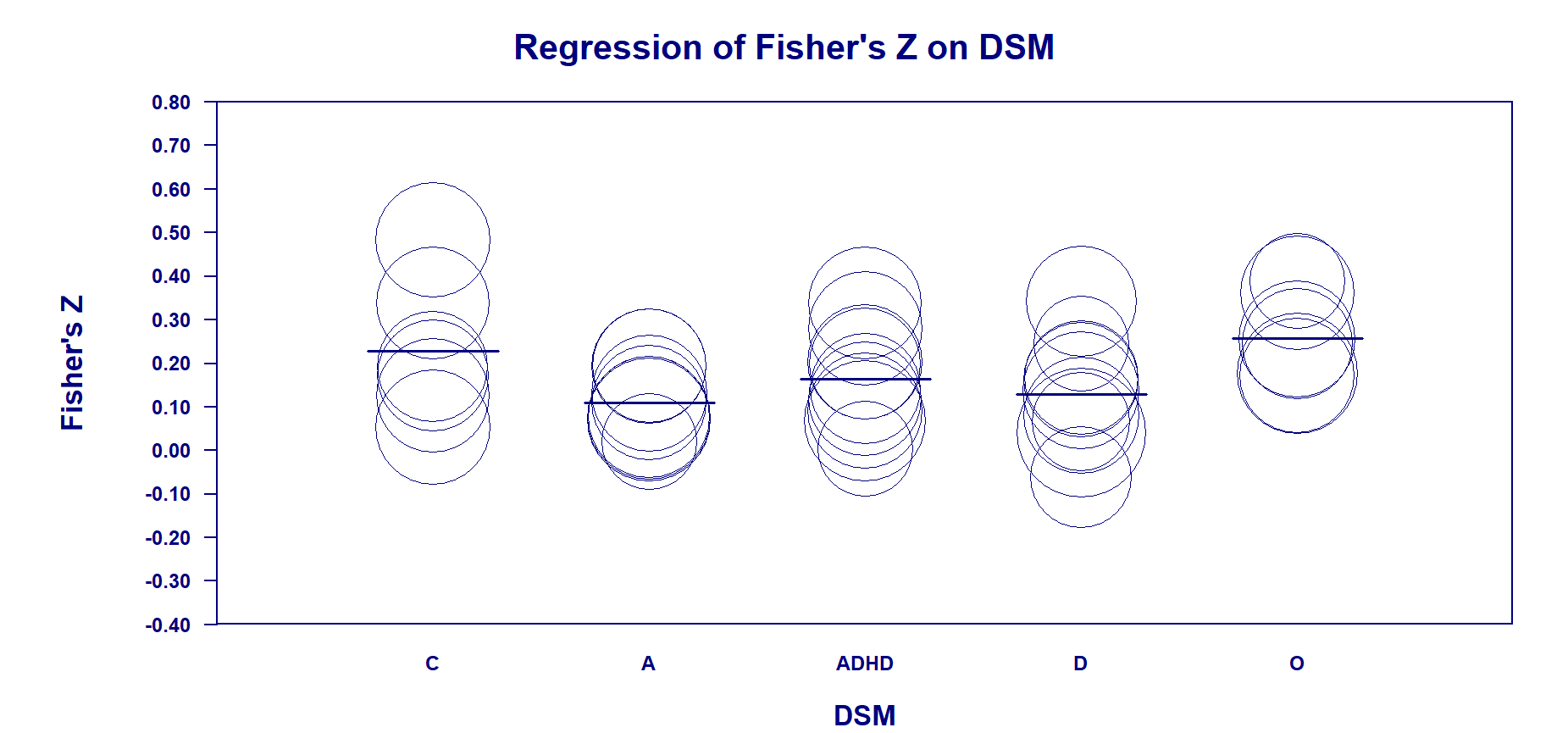


**Figure S6i**

*Model 3 Longitudinal Association: Middle Childhood to Later Childhood/Adolescence Forrest Plot*


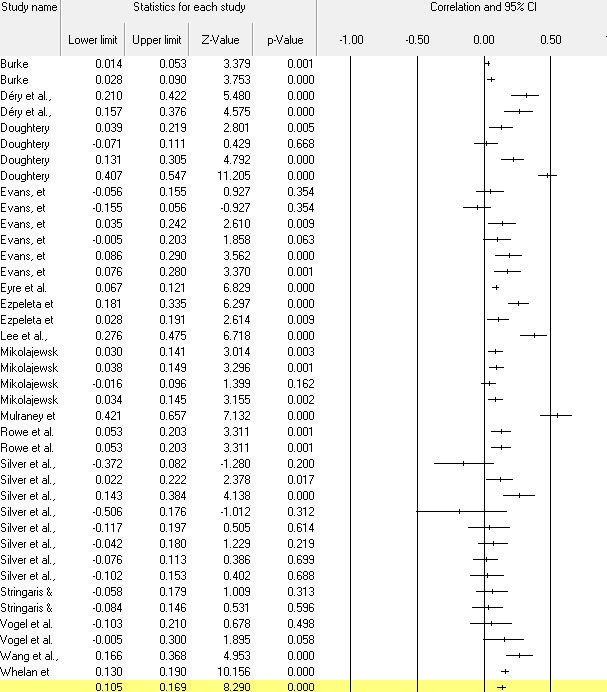


**Figure S6j**

*Model 3 Longitudinal Association: Middle Childhood to Later Childhood/Adolescence Funnel Plot*


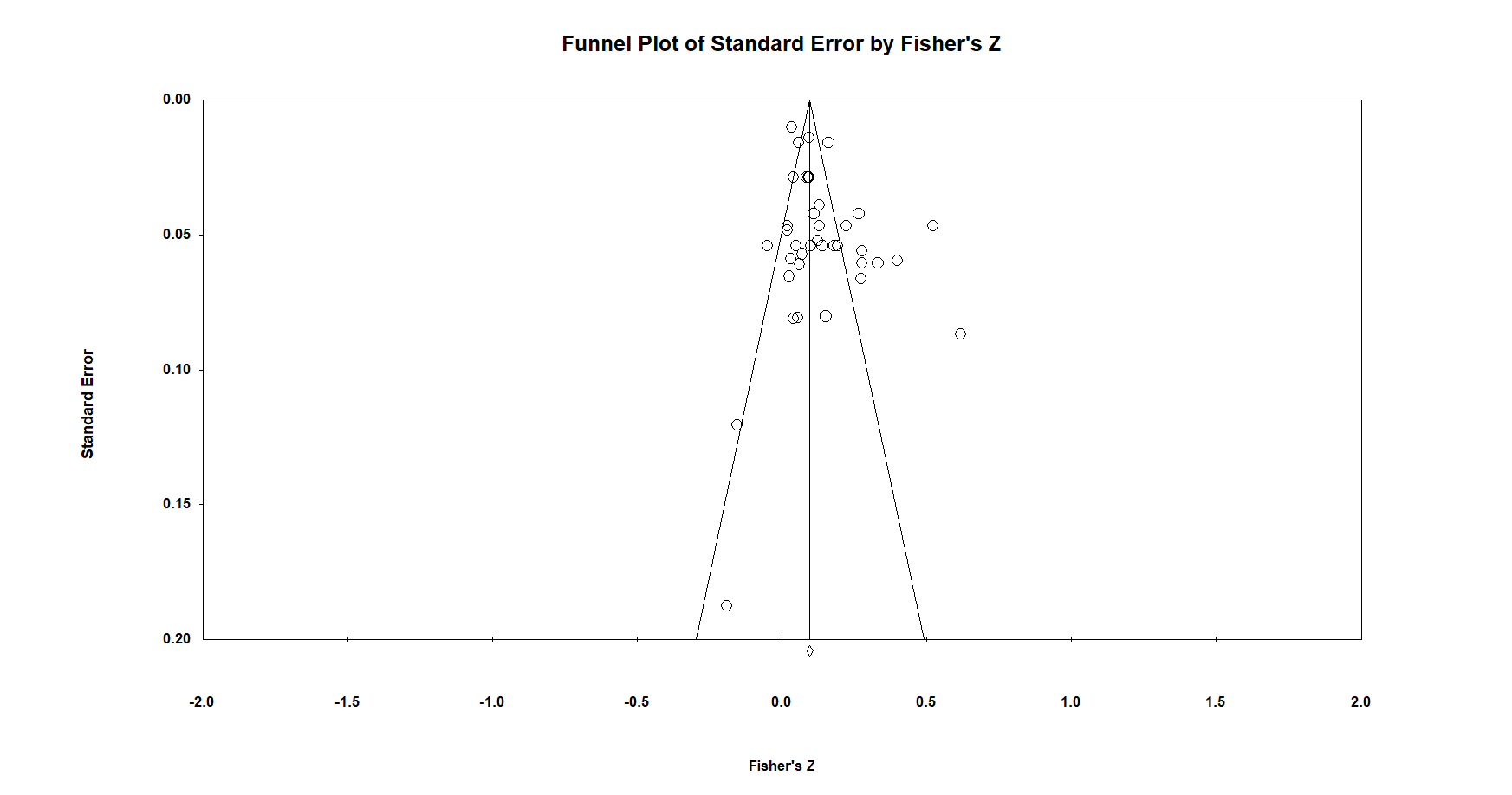


**Figure S6k**

*Model 3 Longitudinal Association: Middle Childhood to Later Childhood/Adolescence Moderation Irritability Measure Scatterplot*


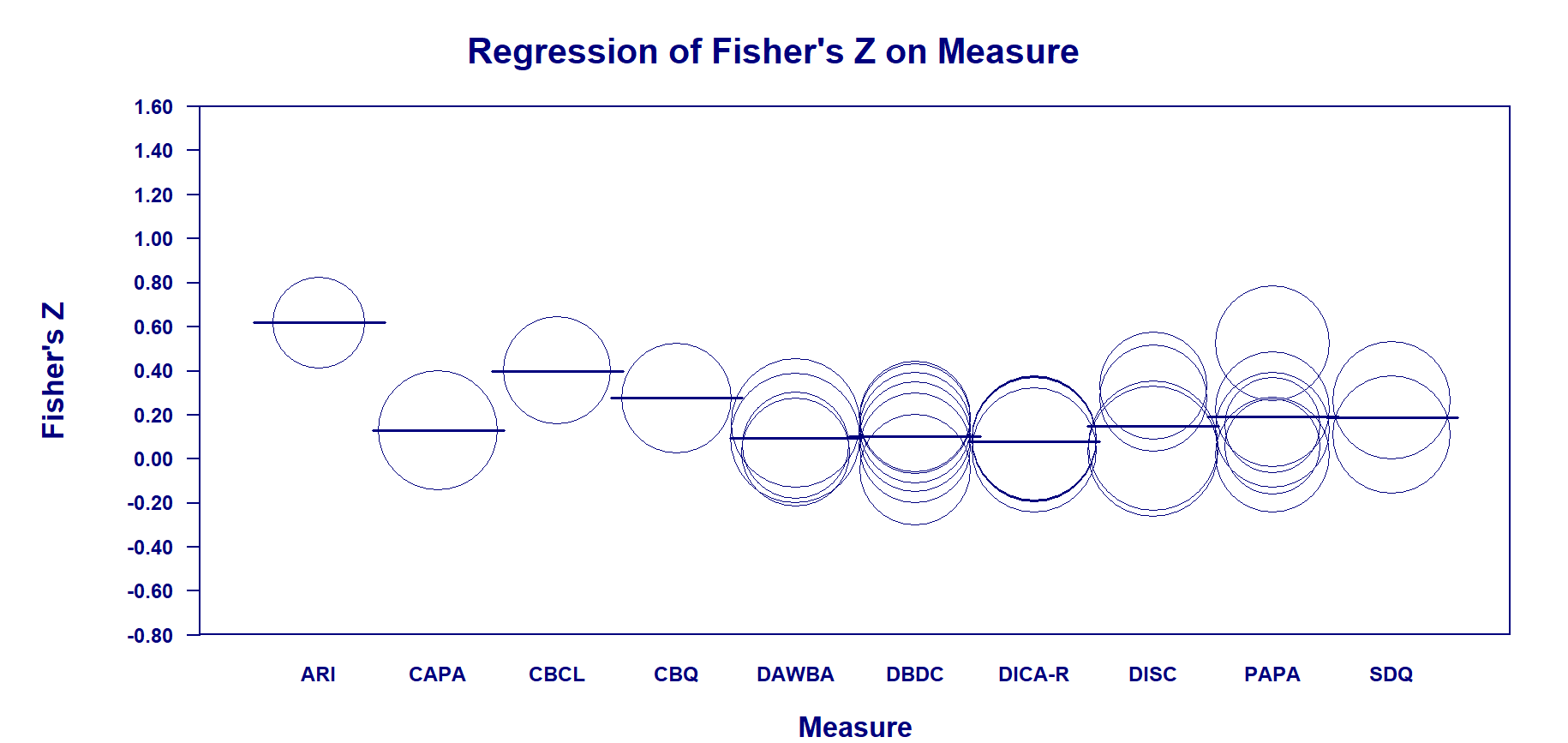


**Figure S6l**

*Model 5 Longitudinal Association Early Adolescence to Late Adolescence Forrest Plot*


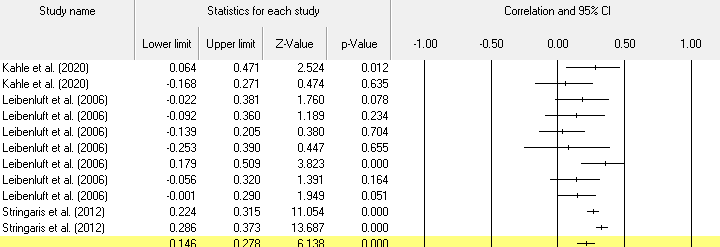


**Figure S6m**

*Model 5 Longitudinal Association Early Adolescence to Late Adolescence Funnel Plot*


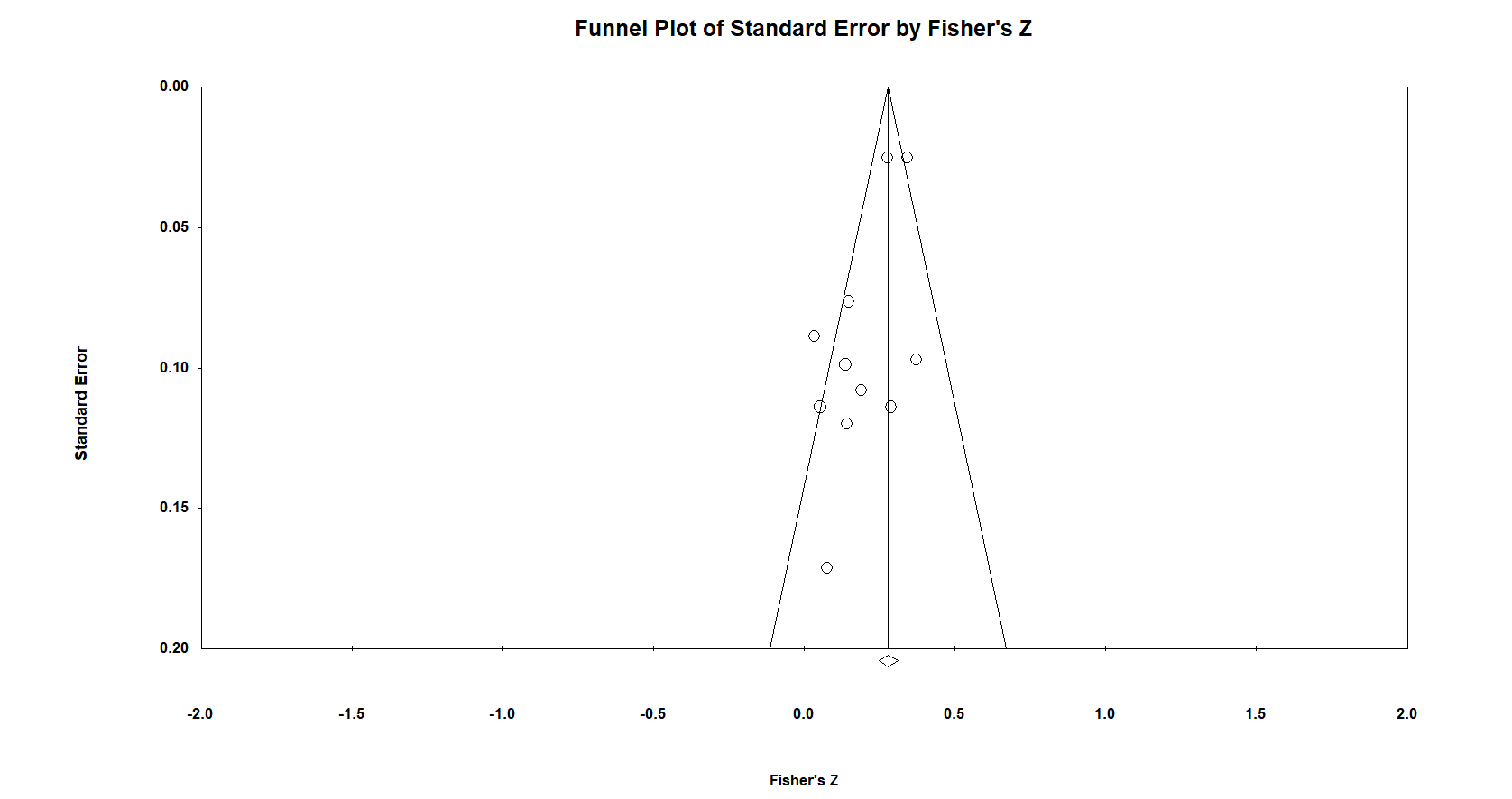


**Figure S6n**

*Model 5 Longitudinal Association Early Adolescence to Late Adolescence Moderation Age Scatterplot*


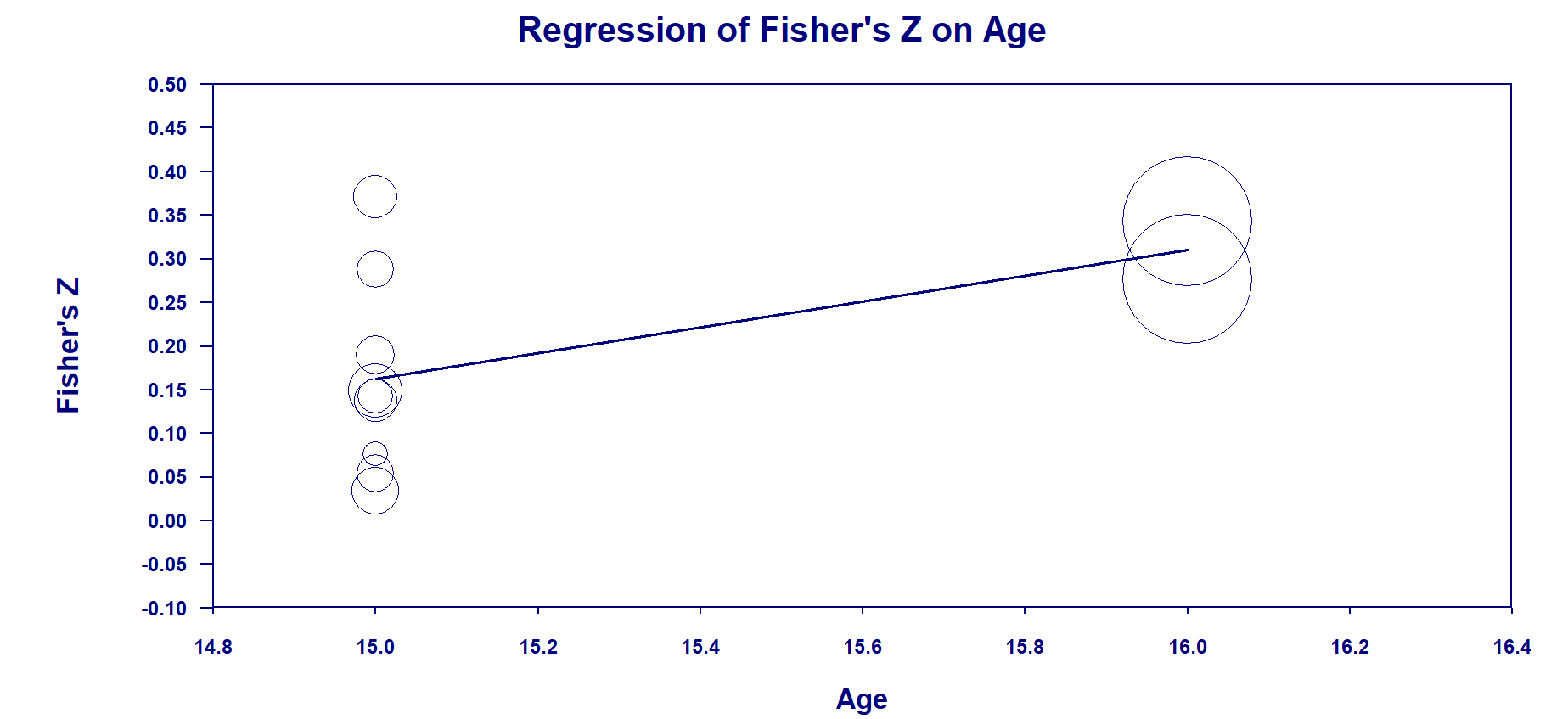


**Figure S6o**

*Model 5 Longitudinal Association Early Adolescence to Late Adolescence Moderation Informant Scatterplot*


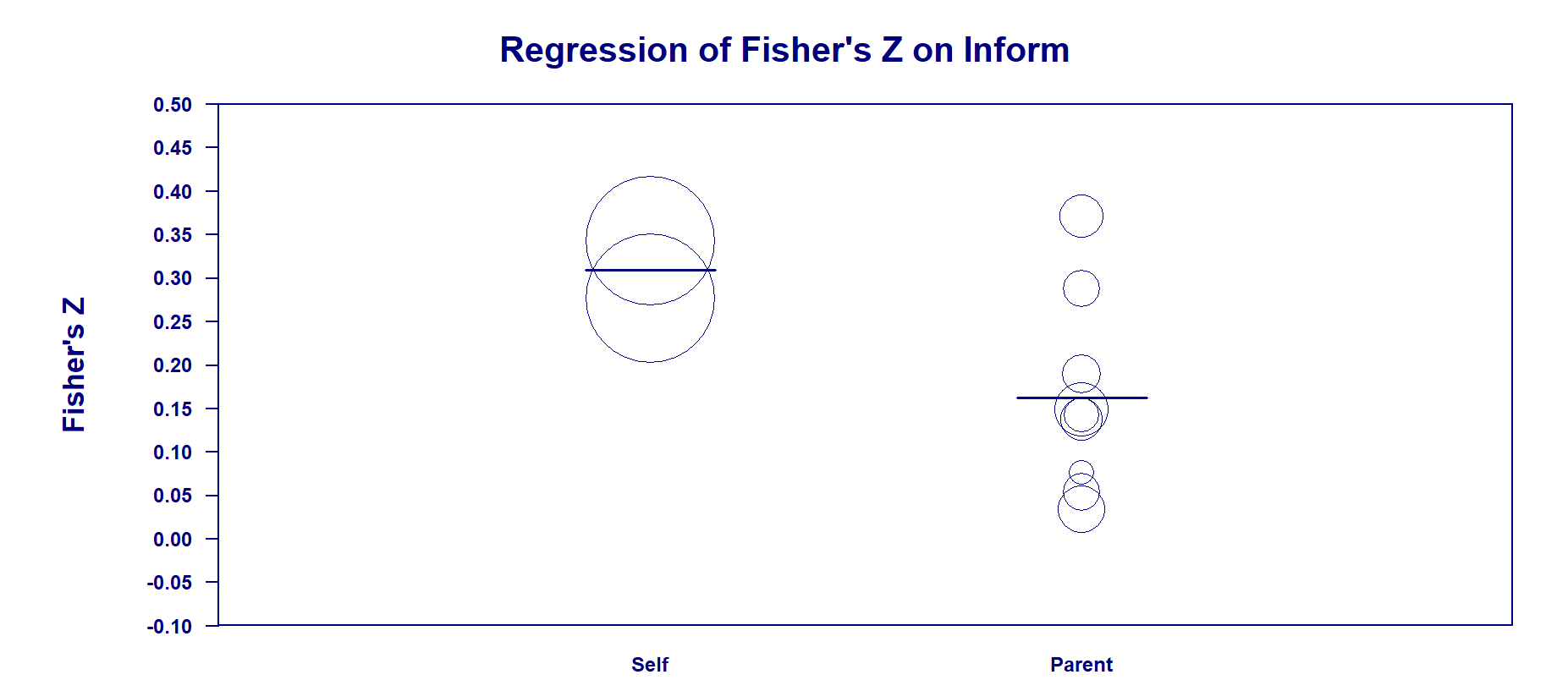


**Figure S6p**

*Model 5 Longitudinal Association Early Adolescence to Late Adolescence Moderation Gender Scatterplot*


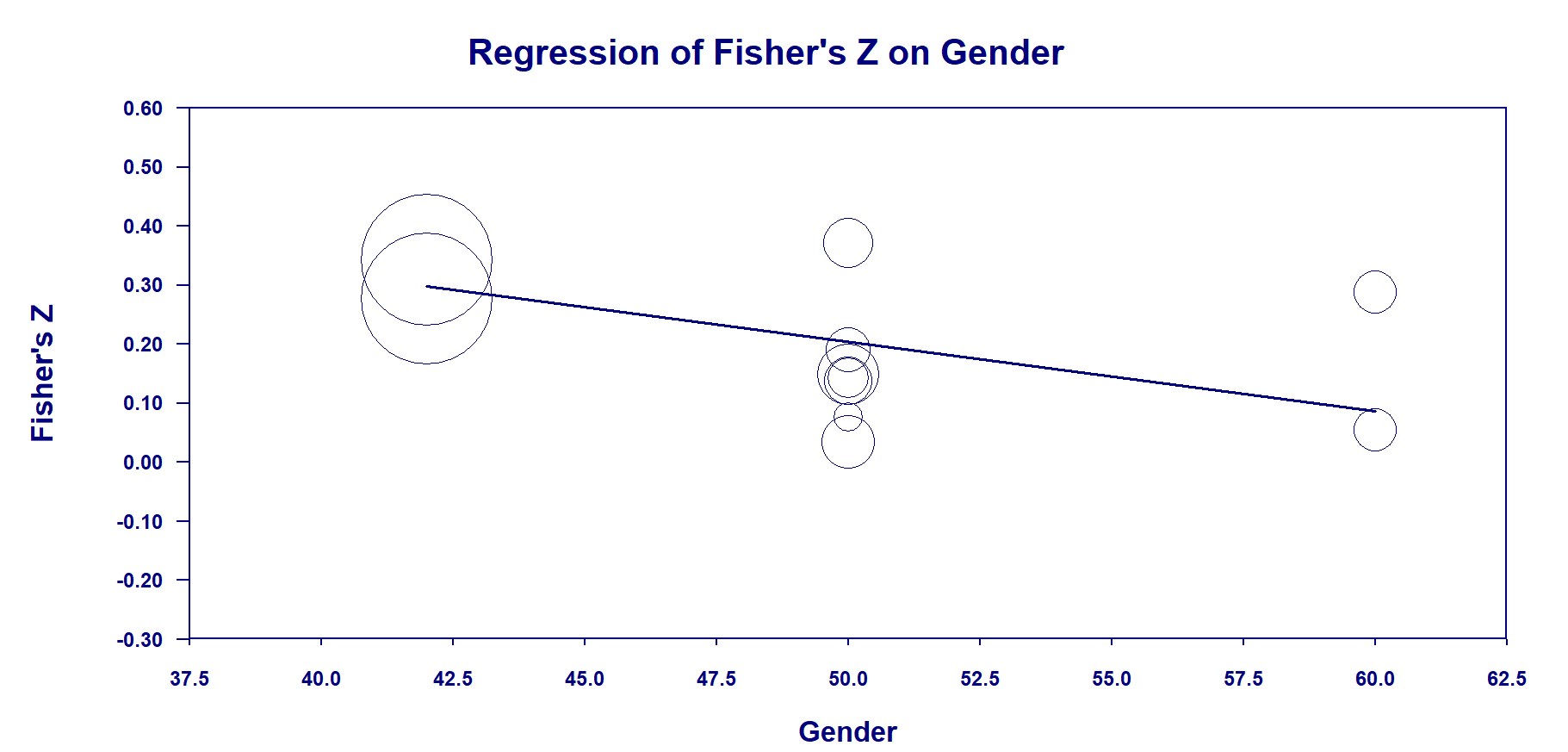


**Figure S6q**

*Model 5 Longitudinal Association Early Adolescence to Late Adolescence Moderation Irritability Measure Scatterplot*


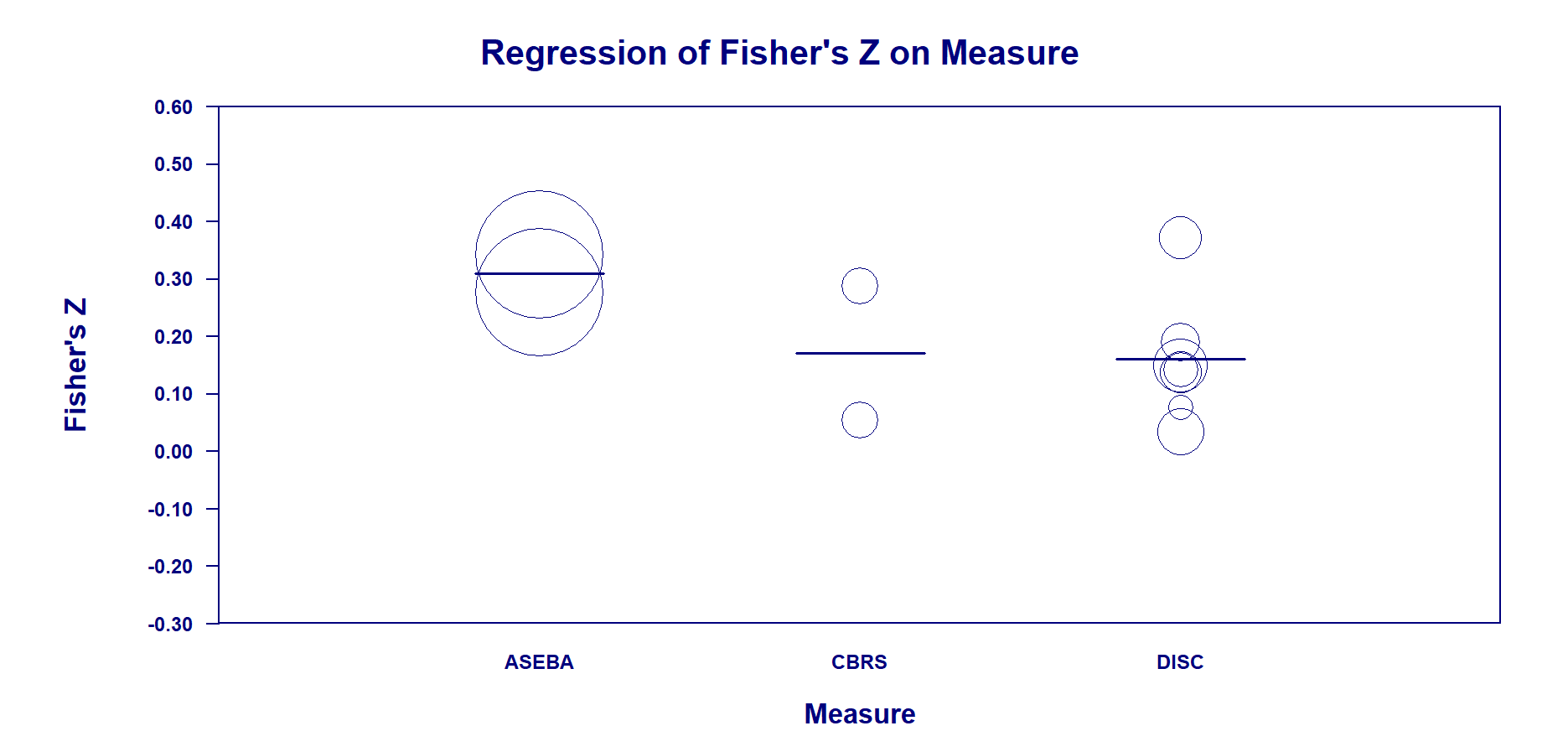


**Figure S6r**

*Model 5 Longitudinal Association Early Adolescence to Late Adolescence Moderation Diagnostic Domains Scatterplot*


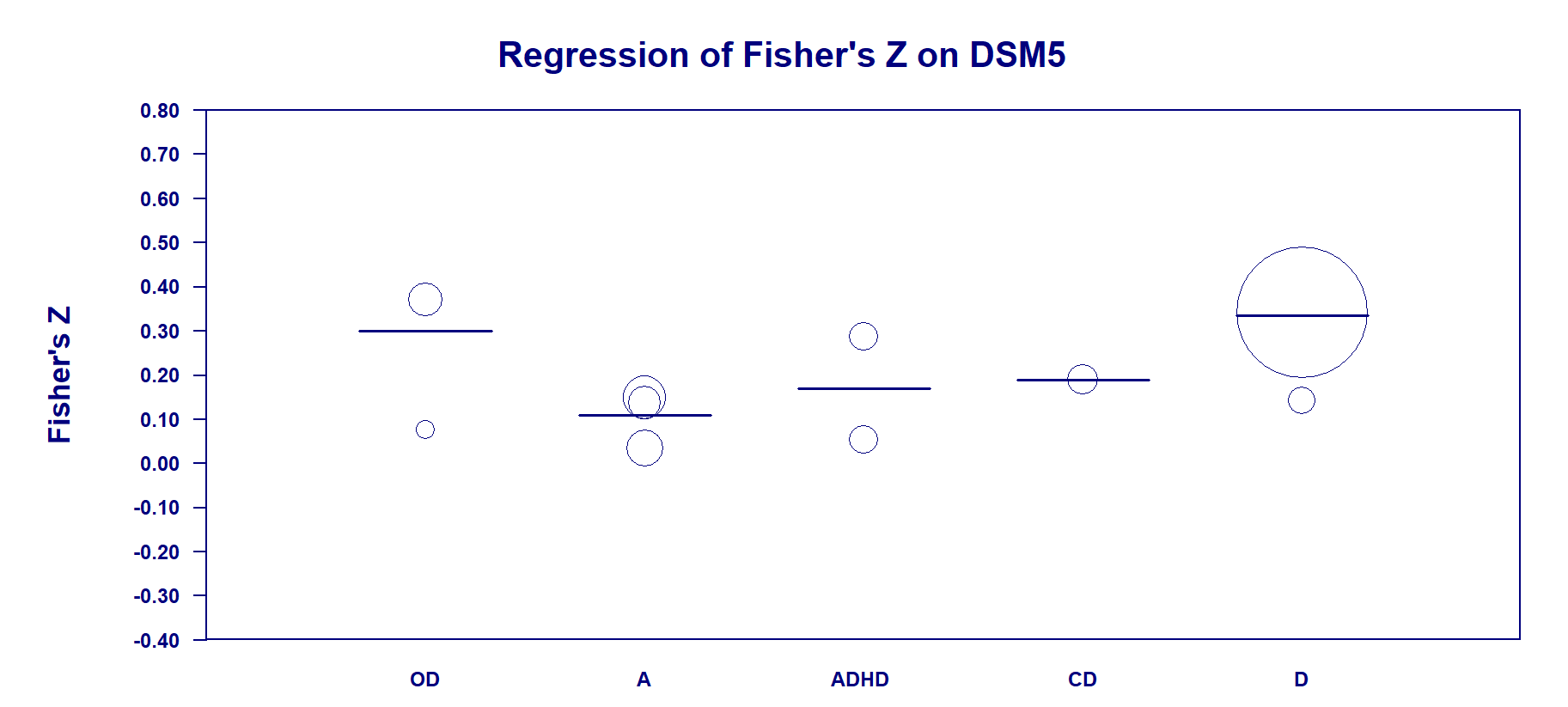


**Figure S6s**

*Model 6 Longitudinal Association Early Adolescence to Adulthood Forrest Plot*


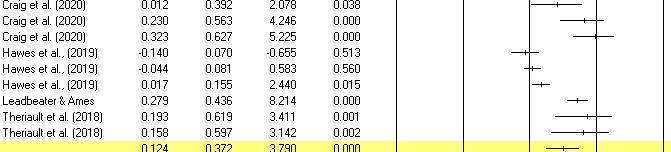


**Figure S6t**

*Model 6 Longitudinal Association Adolescence to Adulthood Funnel Plot*

**
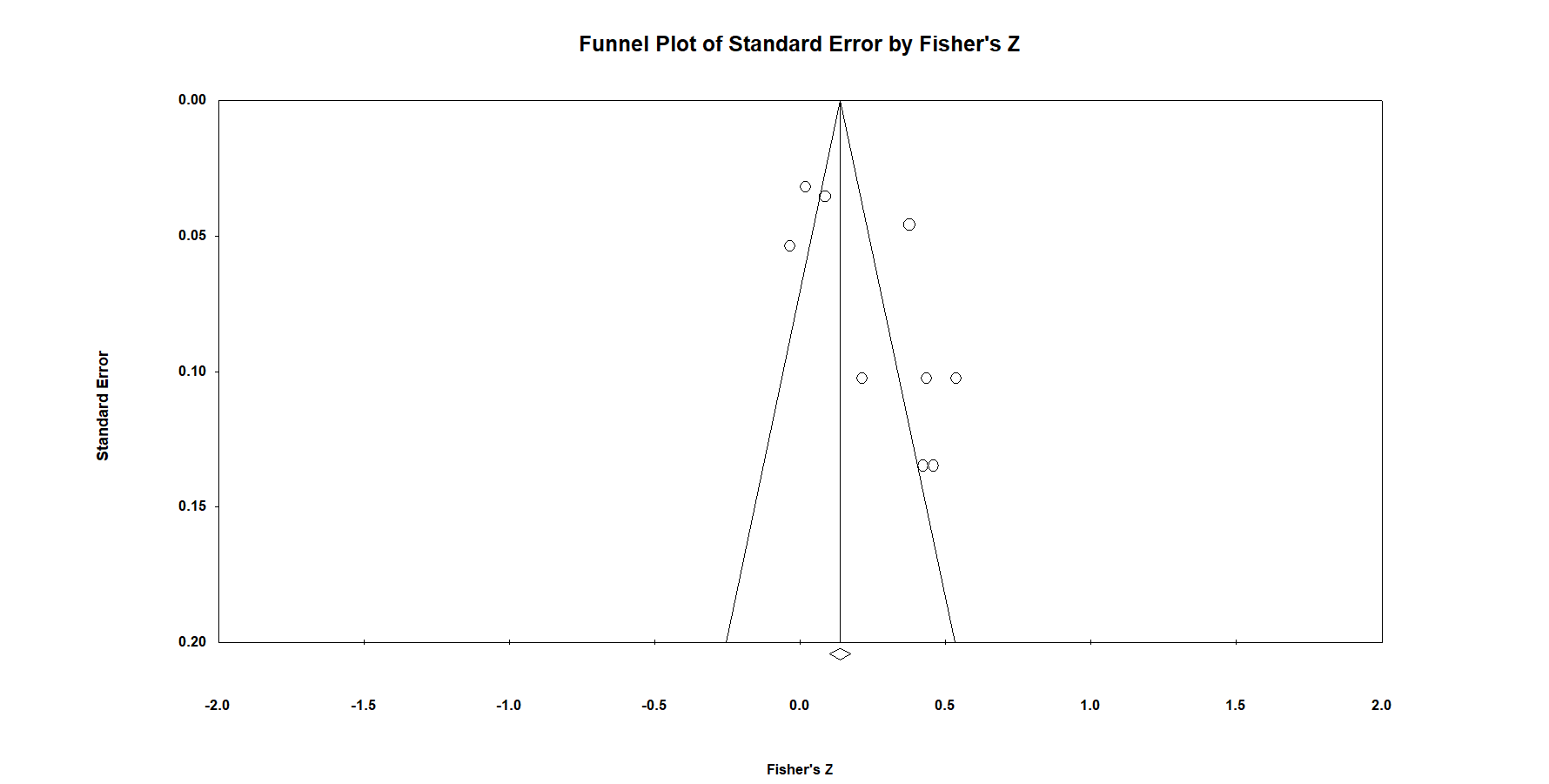
**

**References (Included Studies)**

Aebi, M., Barra, S., Bessler, C., Steinhausen, H. C., Walitza, S., & Plattner, B. (2016). Oppositional defiant disorder dimensions and subtypes among detained male adolescent offenders. *Journal of Child Psychology and Psychiatry*, *57*(6), 729-736. <https://doi.org/10.1111/jcpp.12473>

Aebi, M., Plattner, B., Metzke, C. W., Bessler, C., & Steinhausen, H. C. (2013). Parent- and self-reported dimensions of oppositionality in youth: construct validity, concurrent validity, and the prediction of criminal outcomes in adulthood. *Journal of Child Psychology and Psychiatry*, *54*(9), 941-949. <https://doi.org/10.1111/jcpp.12039>

Ali, O. M., Gabel, L. N., Stanton, K., Kaufman, E. A., Klein, D. N., & Hayden, E. P. (2022). Observational measures of early irritability predict children's psychopathology risk. *Development and Psychopathology*, *34*(4), 1531-1543, <https://doi.org/10.1017/S0954579421000183>

Althoff, R. R., Kuny-Slock, A. V., Verhulst, F. C., Hudziak, J. J., & van der Ende, J. (2014). Classes of oppositional-defiant behavior: concurrent and predictive validity. *Journal of Child Psychology and Psychiatry*, *55*(10), 1162-1171. <https://doi.org/10.1111/jcpp.12233>

Ambrosini, P. J., Bennett, D. S., & Elia, J. (2013). Attention deficit hyperactivity disorder characteristics: II. Clinical correlates of irritable mood. *Journal Affective Disorders, 145*(1), 70-76. <https://doi.org/10.1016/j.jad.2012.07.014>

Arana, C. C., de Pauw, S. S. W., van IJzendoorn, M. H., de Maat, D. A., Kok, R., & Prinzie, P. (2021). No differential susceptibility or diathesis stress to parenting in early adolescence: Personality facets predicting behaviour problems. *Personality and Individual Differences*, *170*, 110406. <https://doi.org/10.1016/j.paid.2020.110406>

Barclay, M. E., Silvers, J. A., & Lee, S. S. (2022). Childhood irritability: predictive validity and mediators of adolescent psychopathology. *Research on Child and Adolescent Psychopathology*, *50*(9), 1165-1177. <https://doi.org/10.1007/s10802-022-00908-2>

Barker, E. D., & Salekin, R. T. (2012). Irritable oppositional defiance and callous unemotional traits: is the association partially explained by peer victimization? *Journal of Child Psychology and Psychiatry*, *53*(11), 1167-1175. <https://doi.org/10.1111/j.1469-7610.2012.02579.x>

Baweja, R., Waschbusch, D. A., Pelham, W. E., 3rd, Pelham, W. E., Jr., & Waxmonsky, J. G. (2021). The impact of persistent irritability on the medication treatment of paediatric attention deficit hyperactivity disorder. *Frontiers in Psychiatry*, *12*, 699687. <https://doi.org/10.3389/fpsyt.2021.699687>

Bell, E., Pooley, A., Tam, P., Boyce, P., Bryant, R., Porter, R., & Malhi, G. S. (2023). A novel exploration of irritability in adolescent males: A preliminary study. *Australasian Psychiatry: Bulletin of the Royal Australian and New Zealand College of Psychiatrists*, *31*(3), 258–262. <https://doi.org/10.1177/10398562221141362>

Benarous, X., Bury, V., Lahaye, H., Desrosiers, L., Cohen, D., & Guilé, J. M. (2020a). Sensory processing difficulties in youths with disruptive mood dysregulation disorder. *Frontiers in Psychiatry*, *11*, 164–164. <https://doi.org/10.3389/fpsyt.2020.00164>

Benarous, X., Renaud, J., Breton, J. J., Cohen, D., Labelle, R., & Guilé, J.M. (2020b). Are youths with disruptive mood dysregulation disorder different from youths with major depressive disorder or persistent depressive disorder? *Journal of Affective Disorders*, *265*, 207–215. <https://doi.org/10.1016/j.jad.2020.01.020>

Bielas, H., Barra, S., Skrivanek, C., Aebi, M., Steinhausen, H.C., Bessler, C., & Plattner, B. (2016). The associations of cumulative adverse childhood experiences and irritability with mental disorders in detained male adolescent offenders. *Child and Adolescent Psychiatry and Mental Health*, *10*(1), 34–34. <https://doi.org/10.1186/s13034-016-0122-7>

Bolhuis, K., Lubke, G. H., van der Ende, J., Bartels, M., van Beijsterveldt, C. E. M., Lichtenstein, P., Larsson, H., Jaddoe, V. W. V., Kushner, S. A., Verhulst, F. C., Boomsma, D. I., & Tiemeier, H. (2017). Disentangling heterogeneity of childhood disruptive behavior problems into dimensions and subgroups. *Journal of the American Academy of Child and Adolescent Psychiatry*, *56*(8), 678–686. <https://doi.org/10.1016/j.jaac.2017.05.019>

Brandes, C. M., Herzhoff, K., Smack, A. J., & Tackett, J. L. (2019). The p factor and the n factor: Associations between the general factors of psychopathology and neuroticism in children. *Clinical Psychological Science*, *7*(6), 1266-1284. <https://doi.org/10.1177/2167702619859332>

Burke, J. D. (2012). An affective dimension within oppositional defiant disorder symptoms among boys: personality and psychopathology outcomes into early adulthood. *Journal of Child Psychology & Psychiatry*, *53*(11), 1176-1183. <https://doi.org/10.1111/j.1469-7610.2012.02598.x>

Burke, J. D., Boylan, K., Rowe, R., Duku, E., Stepp, S. D., Hipwell, A. E., & Waldman, I. D. (2014). Identifying the irritability dimension of ODD: Application of a modified bifactor model across five large community samples of children. *Journal of Abnormal Psychology*, *123*(4), 841-851. <https://doi.org/10.1037/a0037898>

Busch, J., Bleckmann, C., Schettgen, L., Krey, E., & Siefen, G. (2023). A cross-sectional study of children’s irritability determining the links between their ego-resilience and symptoms of anxiety and depression. *Journal of Emotional and Behavioral Disorders*, 106342662211493-. <https://doi.org/10.1177/10634266221149361>

Caprara, G. V., Gerbino, M., Perinelli, E., Alessandri, G., Lenti, C., Walder, M., Preda, C. E., Brunati, E., Marchesini, G., Tiberti, A., Balottin, U., Nonini, L., De Girolamo, G., Meraviglia, C., Gianatti, D., Libera, L., Martinelli, O., Steca, P., Monzani, D., … Nobile, M. (2017). Individual differences in personality associated with aggressive behavior among adolescents referred for externalizing behavior problems. *Journal of Psychopathology and Behavioral Assessment*, *39*(4), 680–692. <https://doi.org/10.1007/s10862-017-9608-8>

Cardinale, E. M., Freitag, G. F., Brotman, M. A., Pine, D. S., Leibenluft, E., & Kircanski, K. (2021). Phasic versus tonic irritability: differential associations with attention deficit hyperactivity disorder symptoms. *Journal of the American Academy of Child and Adolescent Psychiatry*, *60*(12), 1513–1523. <https://doi.org/10.1016/j.jaac.2020.11.022>

Cardinale, E. M., Kircanski, K., Brooks, J., Gold, A. L., Towbin, K. E., Pine, D. S., Leibenluft, E., & Brotman, M. A. (2019). Parsing neurodevelopmental features of irritability and anxiety: Replication and validation of a latent variable approach. *Development and Psychopathology*, *31*(3), 917–929. <https://doi.org/10.1017/S095457941900035X>

Carter Leno, V., Forth, G., Chandler, S., White, P., Yorke, I., Charman, T., Pickles, A., & Simonoff, E. (2021). Behavioural and physiological response to frustration in autistic youth: associations with irritability. *Journal of Neurodevelopmental Disorders*, *13*(1), 27–27. <https://doi.org/10.1186/s11689-021-09374-1>

Chad-Friedman, E., Galano, M. M., Lemay, E. P., Olino, T. M., Klein, D. N., & Dougherty, L. R. (2023). Parsing between- and within-person effects: longitudinal associations between irritability and internalizing and externalizing problems from early childhood through adolescence. *Development & Psychopathology*, *35*(3), 1371-1381. <https://doi.org/10.1017/S0954579421001267>

Chad-Friedman, E., Leppert, K. A., Olino, T. M., Bufferd, S. J., & Dougherty, L. R. (2022). Affective dynamics and mean levels of preschool irritability and sadness: predictors of children's psychological functioning two years later. *Child Psychiatry and Human Development*, *53*(2), 244-255. <https://doi.org/10.1007/s10578-021-01121-w>

Chen, H.W. B., Gardner, E. S., Clarkson, T., Eaton, N. R., Wiggins, J. L., Leibenluft, E., & Jarcho, J. M. (2022). Bullying perpetration and victimization in youth: associations with irritability and anxiety. *Child Psychiatry and Human Development*, *53*(5), 1075–1082. <https://doi.org/10.1007/s10578-021-01192-9>

Copeland, W. E., Brotman, M. A., & Costello, E. J. (2015). Normative irritability in youth: developmental findings from the Great Smoky Mountains study. *Journal of the American Academy of Child and Adolescent Psychiatry*, *54*(8), 635–642. <https://doi.org/10.1016/j.jaac.2015.05.008>

Courbet, O., Slama, H., Purper-Ouakil, D., Massat, I., & Villemonteix, T. (2021). Context-dependent irritability in attention deficit/hyperactivity disorder: correlates and stability of family-restricted versus cross-situational temper outbursts. *Child & Adolescent Mental Health*, *26*(2), 122-133. <https://doi.org/10.1111/camh.12399>

Craig, S. G., Sierra Hernandez, C., Moretti, M. M., & Pepler, D. J. (2021). The mediational effect of affect dysregulation on the association between attachment to parents and oppositional defiant disorder symptoms in adolescents. *Child Psychiatry & Human Development*, *52*, 818-828. <https://doi.org/10.1007/s10578-020-01059-5>

DeGroot, H., Silver, J., Klein, D. N., & Carlson, G. A. (2024). Parent and teacher ratings of tonic and phasic irritability in a clinical sample. *Research on Child and Adolescent Psychopathology*, *52*(6), 891–903. <https://doi.org/10.1007/s10802-023-01151-z>

Déry, M., Lapalme, M., Jagiellowicz, J., Poirier, M., Temcheff, C., & Toupin, J. (2017). Predicting depression and anxiety from oppositional defiant disorder symptoms in elementary school-age girls and boys with conduct problems. *Child Psychiatry & Human Development*, *48*, 53-62. <https://doi.org/10.1007/s10578-016-0652-5>

Doerfler, L. A., Volungis, A. M., & Connor, D. F. (2020). Co-occurrence and differentiation of oppositional defiant and mood disorders among children and adolescents. *Journal of Child and Family Studies*, *29*(9), 2568-2579. <https://doi.org/10.1007/s10826-020-01756-8>

Dougherty, L. R., Smith, V. C., Bufferd, S. J., Kessel, E. M., Carlson, G. A., & Klein, D. N. (2016). Disruptive mood dysregulation disorder at the age of 6 years and clinical and functional outcomes 3 years later. *Psychological Medicine*, *46*(5), 1103-1114. <https://doi.org/10.1017/S0033291715002809>

Dougherty, L. R., Smith, V. C., Bufferd, S. J., Kessel, E., Carlson, G. A., & Klein, D. N. (2015). Preschool irritability predicts child psychopathology, functional impairment, and service use at age nine. *Journal of Child Psychology and Psychiatry*, *56*(9), 999-1007. <https://doi.org/10.1111/jcpp.12403>

Dougherty, L. R., Smith, V. C., Bufferd, S. J., Stringaris, A., Leibenluft, E., Carlson, G. A., & Klein, D. N. (2013). Preschool irritability: longitudinal associations with psychiatric disorders at age 6 and parental psychopathology. *Journal of the American Academy of Child and Adolescent Psychiatry*, *52*(12), 1304-1313. <https://doi.org/10.1016/j.jaac.2013.09.007>

Drabick, D. A., & Gadow, K. D. (2012). Deconstructing oppositional defiant disorder: clinic-based evidence for an anger/irritability phenotype. *Journal of the American Academy of Child and Adolescent Psychiatry*, *51*(4), 384-393. <https://doi.org/10.1016/j.jaac.2012.01.010>

Dugré, J. R., & Potvin, S. (2020). Developmental multi-trajectory of irritability, anxiety, and hyperactivity as psychological markers of heterogeneity in childhood aggression. *Psychological Medicine*, *52*(2), 241–250. <https://doi.org/10.1017/S0033291720001877>

Elvin, O. M., Modecki, K. L., Finch, J., Donnolley, K., Farrell, L. J., & Waters, A. M. (2021). Joining the pieces in childhood irritability: Distinct typologies predict conduct, depressive, and anxiety symptoms. *Behaviour Research & Therapy*, *136*, 103779. <https://doi.org/10.1016/j.brat.2020.103779>

Elvin, O. M., Waters, A. M., & Modecki, K. L. (2023). Does irritability predict attention biases toward threat among clinically anxious youth? *European Child & Adolescent Psychiatry*, *32*(8), 1435–1442. <https://doi.org/10.1007/s00787-022-01954-3>

Evans, S. C., Abel, M. R., Doyle, R. L., Skov, H., & Harmon, S. L. (2021). Measurement and correlates of irritability in clinically referred youth: Further examination of the Affective Reactivity Index. *Journal of Affective Disorders*, *283*, 420-429. <https://doi.org/10.1016/j.jad.2020.11.002>

Evans, S. C., Blossom, J. B., & Fite, P. J. (2020a). Exploring longitudinal mechanisms of irritability in children: implications for cognitive-behavioral intervention. *Behavior Therapy*, *51*(2), 238-252. <https://doi.org/10.1016/j.beth.2019.05.006>

Evans, S. C., Bonadio, F. T., Bearman, S. K., Ugueto, A. M., Chorpita, B. F., & Weisz, J. R. (2020b). Assessing the irritable and defiant dimensions of youth oppositional behavior using CBCL and YSR items. *Journal of Clinical Child and Adolescent Psychology*, *49*(6), 804-819. <https://doi.org/10.1080/15374416.2019.1622119>

Evans, S. C., Cooley, J. L., Blossom, J. B., Pederson, C. A., Tampke, E. C., & Fite, P. J. (2020c). Examining ODD/ADHD symptom dimensions as predictors of social, emotional, and academic trajectories in middle childhood. *Journal of Clinical Child & Adolescent Psychology*, *49*(6), 912-929. <https://doi.org/10.1080/15374416.2019.1644645>

Evans, S. C., Corteselli, K. A., Edelman, A., Scott, H., & Weisz, J. R. (2023). Is irritability a top problem in youth mental health care? A multi-informant, multi-method Investigation. *Child Psychiatry and Human Development*, *54*(4), 1027-1041. <https://doi.org/10.1007/s10578-021-01301-8>

Evans, S. C., Pederson, C. A., Fite, P. J., Blossom, J. B., & Cooley, J. L. (2016). Teacher-Reported irritable and defiant dimensions of oppositional defiant disorder: Social, behavioral, and academic correlates. *School Mental Health*, *8*(2), 292-304. <https://doi.org/10.1007/s12310-015-9163-y>

Eyre, O., Hughes, R. A., Thapar, A. K., Leibenluft, E., Stringaris, A., Davey Smith, G., Stergiakouli, E., Collishaw, S., & Thapar, A. (2019). Childhood neurodevelopmental difficulties and risk of adolescent depression: the role of irritability. *Journal of Child Psychology & Psychiatry*, *60*(8), 866-874. <https://doi.org/10.1111/jcpp.13053>

Eyre, O., Langley, K., Stringaris, A., Leibenluft, E., Collishaw, S., & Thapar, A. (2017). Irritability in ADHD: Associations with depression liability. *Journal of Affective Disorders*, *215*, 281-287. <https://doi.org/10.1016/j.jad.2017.03.050>

Ezpeleta, L., Granero, R., de la Osa, N., Penelo, E., & Domenech, J. M. (2012). Dimensions of oppositional defiant disorder in 3-year-old pre-schoolers. *Journal of Child Psychology & Psychiatry*, *53*(11), 1128-1138. <https://doi.org/10.1111/j.1469-7610.2012.02545.x>

Ezpeleta, L., Penelo, E., de la Osa, N., Navarro, J. B., & Trepat, E. (2019). Irritability and parenting practices as mediational variables between temperament and affective, anxiety, and oppositional defiant problems. *Aggressive Behavior*, *45*(5), 550-560. <https://doi.org/10.1002/ab.21850>

Ezpeleta, L., Penelo, E., de la Osa, N., Navarro, J. B., & Trepat, E. (2020). How the Affective Reactivity Index (ARI) works for teachers as informants. *Journal of Affective Disorders*, *261*, 40-48. <https://doi.org/10.1016/j.jad.2019.09.080>

Ezpeleta, L., Penelo, E., Navarro, J. B., de la Osa, N., & Trepat, E. (2022). Irritability, defiant and obsessive-compulsive problems development from childhood to adolescence. *Journal of Youth and Adolescence*, *51*(6), 1089–1105. <https://doi.org/10.1007/s10964-021-01528-7>

Ezpeleta, L., Penelo, E., Navarro, J. B., Osa, N., & Trepat, E. (2020). Transdiagnostic trajectories of irritability and oppositional, depression and anxiety problems from preschool to early adolescence. *Behaviour Research and Therapy*, *134*, 103727. <https://doi.org/10.1016/j.brat.2020.103727>

Farchione, T. R., Birmaher, B., Axelson, D., Kalas, C., Monk, K., Ehmann, M., Iyengar, S., Kupfer, D., & Brent, D. (2007). Aggression, hostility, and irritability in children at risk for bipolar disorder. *Bipolar Disorders*, *9*(5), 496-503. <https://doi.org/10.1111/j.1399-5618.2007.00390.x>

Fernandez de la Cruz, L., Simonoff, E., McGough, J. J., Halperin, J. M., Arnold, L. E., & Stringaris, A. (2015). Treatment of children with attention-deficit/hyperactivity disorder (ADHD) and irritability: results from the multimodal treatment study of children with ADHD (MTA). *Journal of the American Academy of Child and Adolescent Psychiatry*, *54*(1), 62-70 e3. <https://doi.org/10.1016/j.jaac.2014.10.006>

Filippi, C. A., Subar, A. R., Sachs, J. F., Kircanski, K., Buzzell, G., Pagliaccio, D., Abend, R., Fox, N. A., Leibenluft, E., & Pine, D. S. (2020). Developmental pathways to social anxiety and irritability: The role of the ERN. *Development & Psychopathology*, *34*(3), 1198-1200. <https://doi.org/10.1017/S0954579419001329>

Gadow, K. D., & Drabick, D. A. (2012). Anger and irritability symptoms among youth with ODD: cross-informant versus source-exclusive syndromes. *Journal of Abnormal Child Psychology*, *40*(7), 1073-1085. <https://doi.org/10.1007/s10802-012-9637-4>

Galano, M. M., Stein, S. F., Clark, H. M., Grogan-Kaylor, A., & Graham-Bermann, S. A. (2023). Eight-year trajectories of behavior problems and resilience in children exposed to early-life intimate partner violence: The overlapping and distinct effects of individual factors, maternal characteristics, and early intervention. *Development & Psychopathology*, *35*(2), 850-862. <https://doi.org/10.1017/S0954579422000104>

Grabell, A. S., Jones, H. M., Wilett, A. E., Bemis, L. M., Wakschlag, L. S., & Perlman, S. B. (2020). Children's facial muscular movements and risk for early psychopathology: Assessing clinical utility. *Behavior Therapy*, *51*(2), 253-267. <https://doi.org/10.1016/j.beth.2019.08.004>

Guzick, A. G., Geller, D. A., Small, B. J., Murphy, T. K., Wilhelm, S., & Storch, E. A. (2021). Irritability in children and adolescents with OCD. *Behavior Therapy*, *52*(4), 883-896. <https://doi.org/10.1016/j.beth.2020.11.001>

Harima, Y., Miyawaki, D., Goto, A., Hirai, K., Sakamoto, S., Hama, H., Kadono, S., Nishiura, S., & Inoue, K. (2022). Associations between chronic irritability and sensory processing difficulties in children and adolescents. *Frontiers in Psychiatry*, *13*, 860278–860278. <https://doi.org/10.3389/fpsyt.2022.860278>

Hawes, M. T., Carlson, G. A., Finsaas, M. C., Olino, T. M., Seely, J. R., & Klein, D. N. (2020). Dimensions of irritability in adolescents: longitudinal associations with psychopathology in adulthood. *Psychological Medicine*, *50*(16), 2759-2767. <https://doi.org/10.1017/S0033291719002903>

Kahle, S., Mukherjee, P., Dixon, J. F., Leibenluft, E., Hinshaw, S. P., & Schweitzer, J. B. (2021). Irritability predicts hyperactive/impulsive symptoms across adolescence for females. *Research on Child and Adolescent Psychopathology*, *49*(2), 185-196. <https://doi.org/10.1007/s10802-020-00723-7>

Kalvin, C. B., Gladstone, T. R., Jordan, R., Rowley, S., Marsh, C. L., Ibrahim, K., & Sukhodolsky, D. G. (2021). Assessing irritability in children with autism spectrum disorder using the affective reactivity index. *Journal of Autism and Developmental Disorders*, *51*(5), 1496-1507. <https://doi.org/10.1007/s10803-020-04627-9>

Kessel, E. M., Frost, A., Goldstein, B. L., Black, S. R., Dougherty, L. R., Carlson, G. A., & Klein, D. N. (2021). Developmental pathways from preschool irritability to multifinality in early adolescence: the role of diurnal cortisol. *Psychological Medicine*, *51*(5), 761-769. <https://doi.org/10.1017/S0033291719003684>

Kessel, E. M., Kujawa, A., Dougherty, L. R., Hajcak, G., Carlson, G. A., & Klein, D. N. (2017). Neurophysiological processing of emotion in children of mothers with a history of depression: the moderating role of preschool persistent irritability. *Journal of Abnormal Child Psychology*, *45*(8), 1599-1608. <https://doi.org/10.1007/s10802-017-0272-y>

Kessel, E. M., Meyer, A., Hajcak, G., Dougherty, L. R., Torpey-Newman, D. C., Carlson, G. A., & Klein, D. N. (2016). Transdiagnostic factors and pathways to multifinality: The error-related negativity predicts whether preschool irritability is associated with internalizing versus externalizing symptoms at age 9. *Development & Psychopathology*, *28*(4pt1), 913-926. <https://doi.org/10.1017/S0954579416000626>

Khurana, S., Wei, M. A., Karlovich, A. R., & Evans, S. C. (2023). Irritability and suicidality in clinically referred youth: Clarifying the link by examining the roles of age and hope. *Journal of Psychopathology and Behavioral Assessment*, *45*(3), 640–649. <https://doi.org/10.1007/s10862-023-10049-5>

Kishida, K., Tsuda, M., Takahashi, F., & Ishikawa, S. I. (2022). Irritability and mental health profiles among children and adolescents: A result of latent profile analysis. *Journal of Affective Disorders*, *300*, 76-83. <https://doi.org/10.1016/j.jad.2021.12.045>

Kolko, D. J., & Pardini, D. A. (2010). ODD dimensions, ADHD, and callous-unemotional traits as predictors of treatment response in children with disruptive behavior disorders. *Journal of Abnormal Psychology*, *119*(4), 713-725. <https://doi.org/10.1037/a0020910>

Kolko, D. J., Baumann, B. L., Bukstein, O. G., & Brown, E. J. (2007). Internalizing symptoms and affective reactivity in relation to the severity of aggression in clinically referred, behavior-disordered children. *Journal of Child and Family Studies*, *16*(6), 745-759. <https://doi.org/10.1007/s10826-006-9120-3>

Krieger, F. V., Polanczyk, V. G., Goodman, R., Rohde, L. A., Graeff-Martins, A. S., Salum, G., Gadelha, A., Pan, P., Stahl, D., & Stringaris, A. (2013). Dimensions of oppositionality in a Brazilian community sample: testing the DSM-5 proposal and etiological links. *Journal of the American Academy of Child and Adolescent Psychiatry*, *52*(4), 389-400.e1. <https://doi.org/10.1016/j.jaac.2013.01.004>

Leadbeater, B. J., & Ames, M. E. (2017). The longitudinal effects of oppositional defiant disorder symptoms on academic and occupational functioning in the transition to young adulthood. *Journal of Abnormal Child Psychology*, *45*(4), 749-763. <https://doi.org/10.1007/s10802-016-0190-4>

Lee, K. S., Lebowitz, E. R., Silverman, W. K., & Tseng, W. (2023). Transactional associations of child irritability and anxiety with parent psychological control in Taiwanese school‐aged children. *JCPP Advances*, *3*(4), e12192-n/a. <https://doi.org/10.1002/jcv2.12192>

Legenbauer, T., Hübner, J., Pinnow, M., Ball, A., Pniewski, B., & Holtmann, M. (2018). Proper emotion recognition, dysfunctional emotion regulation: the mystery of affective dysregulation in adolescent psychiatric inpatients. *Zeitschrift für Kinder- und Jugendpsychiatrie und Psychotherapie*, *46*(1), 7–16. <https://doi.org/10.1024/1422-4917/a000479>

Leibenluft, E., Cohen, P., Gorrindo, T., Brook, J. S., & Pine, D. S. (2006). Chronic versus episodic irritability in youth: a community-based, longitudinal study of clinical and diagnostic associations. *Journal of Child & Adolescent Psychopharmacology*, *16*(4), 456-466. <https://doi.org/10.1089/cap.2006.16.456>

Leigh, E., Lee, A., Brown, H. M., Pisano, S., & Stringaris, A. (2020). A prospective study of rumination and irritability in youth. *Journal of Abnormal Child Psychology, 48*(12), 1581-1589. <https://doi.org/10.1007/s10802-020-00706-8>

Lengua, L. J. (2006). Growth in temperament and parenting as predictors of adjustment during children's transition to adolescence. *Developmental Psychology*, *42*(5), 819-832. <https://doi.org/10.1037/0012-1649.42.5.819>

Lengua, L. J., & Kovacs, E. A. (2005). Bidirectional associations between temperament and parenting and the prediction of adjustment problems in middle childhood. *Journal of Applied Developmental Psychology*, *26*(1), 21-38. <https://doi.org/10.1016/j.appdev.2004.10.001>

Levy, T., Kronenberg, S., Crosbie, J., & Schachar, R. J. (2020). Attention-deficit/hyperactivity disorder (ADHD) symptoms and suicidality in children: The mediating role of depression, irritability, and anxiety symptoms. *Journal of Affective Disorders*, *265*, 200-206. <https://doi.org/10.1016/j.jad.2020.01.022>

Liu, W., Guo, X., Liu, F., & Sun, Y. (2024). The role of emotion regulation strategies in the relationship between temperament and depression in preadolescents. *Child Psychiatry and Human Development*, *55*(2), 439–452. <https://doi.org/10.1007/s10578-022-01423-7>

Loram, G., Silk, T., Ling, M. T., Fuller-Tyszkiewicz, M., Hyde, C. S., McGillivray, J., & Sciberras, E. (2021). Associations between sleep, daytime sleepiness and functional outcomes in adolescents with ADHD [Article]. Sleep Medicine, 87, 174-182. <https://doi.org/10.1016/j.sleep.2021.08.021>

Maire, J., Galera, C., Bioulac, S., Bouvard, M., & Michel, G. (2020). Emotional lability and irritability have specific associations with symptomatology in children with attention deficit hyperactivity disorder. *Psychiatry Research*, *285*, 112789. <https://doi.org/10.1016/j.psychres.2020.112789>

Martin, S. E., Hunt, J. I., Mernick, L. R., DeMarco, M., Hunter, H. L., Coutinho, M. T., & Boekamp, J. R. (2017). Temper loss and persistent irritability in pre-schoolers: implications for diagnosing disruptive mood dysregulation disorder in early childhood. *Child Psychiatry and Human Development*, *48*(3), 498-508. <https://doi.org/10.1007/s10578-016-0676-x>

Mikolajewski, A. J., Taylor, J., & Iacono, W. G. (2017). Oppositional defiant disorder dimensions: genetic influences and risk for later psychopathology. *Journal of Child Psychology & Psychiatry*, *58*(6), 702-710. <https://doi.org/10.1111/jcpp.12683>

Mulraney, M., Melvin, G., & Tonge, B. (2014). Brief report: can irritability act as a marker of psychopathology? *Journal of Adolescence*, *37*(4), 419-423. <https://doi.org/10.1016/j.adolescence.2014.03.005>

Mulraney, M., Zendarski, N., Mensah, F., Hiscock, H., & Sciberras, E. (2017). Do early internalizing and externalizing problems predict later irritability in adolescents with attention-deficit/hyperactivity disorder? *Australian and New Zealand Journal of Psychiatry*, *51*(4), 393-402. <https://doi.org/10.1177/0004867416659365>

Naim, R., Smith, A., Chue, A., Grassie, H., Linke, J., Dombek, K., Shaughnessy, S., McNeil, C., Cardinale, E., Agorsor, C., Cardenas, S., Brooks, J., Subar, A. R., Jones, E. L., Do, Q. B., Pine, D. S., Leibenluft, E., Brotman, M. A., & Kircanski, K. (2021). Using ecological momentary assessment to enhance irritability phenotyping in a transdiagnostic sample of youth. *Development and psychopathology*, *33*(5), 1734-1746. <https://doi.org/10.1017/S0954579421000717>

Nelson, S., Moorman, E., Farrell, M., & Cunningham, N. (2018). Irritability is common and is related to poorer psychosocial outcomes in youth with functional abdominal pain disorders (FAPD). *Children*, *5*(4), 52. <https://doi.org/10.3390/children5040052>

Pan, P. Y., & Yeh, C. B. (2019). Irritability and maladaptation among children: the utility of Chinese versions of the affective reactivity index and aberrant behavior checklist-irritability subscale. *Journal of Child & Adolescent Psychopharmacology*, *29*(3), 213-219. <https://doi.org/10.1089/cap.2018.0070>

Perhamus, G. R., & Ostrov, J. M. (2021). Emotions and cognitions in early childhood aggression: the role of irritability and hostile attribution biases. *Research on Child and Adolescent Psychopathology*, *49*(1), 63-75. <https://doi.org/10.1007/s10802-020-00707-7>

Poznanski, B., Cornacchio, D., Coxe, S., Pincus, D. B., McMakin, D. L., & Comer, J. S. (2018). The link between anxiety severity and irritability among anxious youth: evaluating the mediating role of sleep problems. *Child Psychiatry and Human Development*, *49*(3), 352-359. <https://doi.org/10.1007/s10578-017-0769-1>

Rappaport, L. M., Carney, D. M., Brotman, M. A., Leibenluft, E., Pine, D. S., Roberson-Nay, R., & Hettema, J. M. (2020). A population-based twin study of childhood irritability and internalizing syndromes. *Journal of Clinical Child and Adolescent Psychology*, *49*(4), 524-534. <https://doi.org/10.1080/15374416.2018.1514612>

Rice, F., Sellers, R., Hammerton, G., Eyre, O., Bevan-Jones, R., Thapar, A. K., Collishaw, S., Harold, G. T., & Thapar, A. (2017). Antecedents of new-onset major depressive disorder in children and adolescents at high familial risk. *JAMA Psychiatry*, *74*(2), 153-160. <https://doi.org/10.1001/jamapsychiatry.2016.3140>

Rowe, R., Costello, E. J., Angold, A., Copeland, W. E., & Maughan, B. (2010). Developmental pathways in oppositional defiant disorder and conduct disorder. *Journal of Abnormal Psychology*, *119*(4), 726-738. <https://doi.org/10.1037/a0020798>

Rubens, S. L., Evans, S. C., Becker, S. P., Fite, P. J., & Tountas, A. M. (2017). Self-reported time in bed and sleep quality in association with internalizing and externalizing symptoms in School-Age Youth. *Child Psychiatry and Human Development*, *48*(3), 455–467. <https://doi.org/10.1007/s10578-016-0672-1>

Silver, J., Carlson, G. A., Olino, T. M., Perlman, G., Mackin, D., Kotov, R., & Klein, D. N. (2021). Differential outcomes of tonic and phasic irritability in adolescent girls. *Journal of Child Psychology & Psychiatry*, *62*(10), 1220-1227. <https://doi.org/10.1111/jcpp.13402>

Silver, J., Sorcher, L., Carlson, G. A., Dougherty, L. R., & Klein, D. N. (2024). Irritability across adolescence: Examining longitudinal trajectory, stability, and associations with psychopathology and functioning at age 18. *Journal of Affective Disorders*, *354*, 611–618. <https://doi.org/10.1016/j.jad.2024.03.079>

Smith, J. D., Wakschlag, L., Krogh-Jespersen, S., Walkup, J. T., Wilson, M. N., Dishion, T. J., & Shaw, D. S. (2019). Dysregulated irritability as a window on young children's psychiatric risk: Transdiagnostic effects via the family check-up. *Development and Psychopathology*, *31*(5), 1887-1899. <https://doi.org/10.1017/S0954579419000816>

Sorcher, L. K., Goldstein, B. L., Finsaas, M. C., Carlson, G. A., Klein, D. N., & Dougherty, L. R. (2022). Preschool irritability predicts adolescent psychopathology and functional impairment: A 12-Year prospective study. *Journal of the American Academy of Child and Adolescent Psychiatry*, *61*(4), 554-564 e1. <https://doi.org/10.1016/j.jaac.2021.08.016>

Srinivasan, R., Flouri, E., Lewis, G., Solmi, F., Stringaris, A., & Lewis, G. (2024). Changes in early childhood irritability and its association with depressive symptoms and self-harm during adolescence in a nationally representative United Kingdom birth cohort. *Journal of the American Academy of Child and Adolescent Psychiatry, 63*(1), 39–51. <https://doi.org/10.1016/j.jaac.2023.05.027>

Stoddard, J., Tseng, W. L., Kim, P., Chen, G., Yi, J., Donahue, L., Brotman, M. A., Towbin, K. E., Pine, D. S., & Leibenluft, E. (2017). Association of irritability and anxiety with the neural mechanisms of implicit face emotion processing in youths with psychopathology. *JAMA Psychiatry*, *74*(1), 95-103. <https://doi.org/10.1001/jamapsychiatry.2016.3282>

Stringaris, A., & Goodman, R. (2009a). Three dimensions of oppositionality in youth. *Journal of Child Psychology & Psychiatry*, *50*(3), 216-223. <https://doi.org/10.1111/j.1469-7610.2008.01989.x>

Stringaris, A., & Goodman, R. (2009b). Longitudinal outcome of youth oppositionality: irritable, headstrong, and hurtful behaviors have distinctive predictions. *Journal of the American Academy of Child and Adolescent Psychiatry*, *48*(4), 404-412. <https://doi.org/10.1097/CHI.0b013e3181984f30>

Stringaris, A., Zavos, H., Leibenluft, E., Maughan, B., & Eley, T. C. (2012). Adolescent irritability: phenotypic associations and genetic links with depressed mood. *The American Journal of Psychiatry*, *169*(1), 47-54. <https://doi.org/10.1176/appi.ajp.2011.10101549>

Theriault, M. G., Becue, J. C., Lesperance, P., Chouinard, S., Rouleau, G. A., & Richer, F. (2018). Oppositional behavior and longitudinal predictions of early adulthood mental health problems in chronic tic disorders. *Psychiatry Research*, *266*, 301-308. <https://doi.org/10.1016/j.psychres.2018.03.026>

Ucar, H. N., & Vural, A. P. (2018). Irritability and parenting styles in adolescents with attention-deficit/hyperactivity disorder: A controlled study. *Journal of Psychosocial Nursing and Mental Health Services*, *56*(9), 33-43. <https://doi.org/10.3928/02793695-20180412-02>

Valencia, F., Penelo, E., de la Osa, N., Navarro, J. B., & Ezpeleta, L. (2021). Prospective association of parental and child internalizing symptoms: Mediation of parenting practices and irritability. *British Journal of Developmental Psychology*, *39*(3), 363-379. <https://doi.org/10.1111/bjdp.12367>

Vogel, A. C., Jackson, J. J., Barch, D. M., Tillman, R., & Luby, J. L. (2019). Excitability and irritability in pre-schoolers predict later psychopathology: The importance of positive and negative emotion dysregulation. *Development & Psychopathology*, *31*(3), 1067-1083. <https://doi.org/10.1017/S0954579419000609>

Wakschlag, L. S., Estabrook, R., Petitclerc, A., Henry, D., Burns, J. L., Perlman, S. B., Voss, J. L., Pine, D. S., Leibenluft, E., & Briggs-Gowan, M. L. (2015). Clinical implications of a dimensional approach: the normal: abnormal spectrum of early irritability. *Journal of the American Academy of Child and Adolescent Psychiatry*, *54*(8), 626-634. <https://doi.org/10.1016/j.jaac.2015.05.016>

Wakschlag, L. S., Krogh-Jespersen, S., Estabrook, R., Hlutkowsky, C. O., Anderson, E. L., Burns, J., Briggs-Gowan, M. J., Petitclerc, A., & Perlman, S. B. (2020). The early childhood irritability-related impairment interview (E-CRI): A novel method for assessing young children's developmentally impairing irritability. *Behavior Therapy*, *51*(2), 294-309. <https://doi.org/10.1016/j.beth.2019.07.008>

Wang, F., Wang, M., Wang, X., & Zhao, J. (2023). Child- and family-level factors as predictors of Chinese children’s generalized anxiety disorder symptoms in middle childhood. *Current Psychology 42*(29), 25061–25074. <https://doi.org/10.1007/s12144-022-03583-0>

Waschbusch, D. A., Baweja, R., Babinski, D. E., Mayes, S. D., & Waxmonsky, J. G. (2020). Irritability and limited prosocial emotions/callous-unemotional traits in elementary-school-age children. *Behavior Therapy*, *51*(2), 223-237. <https://doi.org/10.1016/j.beth.2019.06.007>

Waxmonsky, J. G., Fosco, W., Waschbusch, D., Babinski, D., Baweja, R., Pegg, S., Cao, V., Shroff, D., & Kujawa, A. (2022). The impact of irritability and callous unemotional traits on reward positivity in youth with ADHD and conduct problems. *Research on Child and Adolescent Psychopathology*, *50*(8), 1027-1040. <https://doi.org/10.1007/s10802-022-00901-9>

Waxmonsky, J. G., Mayes, S. D., Calhoun, S. L., Fernandez-Mendoza, J., Waschbusch, D. A., Bendixsen, B. H., & Bixler, E. O. (2017). The association between disruptive mood dysregulation disorder symptoms and sleep problems in children with and without ADHD. *Sleep Medicine*, *37*, 180-186. <https://doi.org/10.1016/j.sleep.2017.02.006>

Whelan, Y. M., Leibenluft, E., Stringaris, A., & Barker, E. D. (2015). Pathways from maternal depressive symptoms to adolescent depressive symptoms: the unique contribution of irritability symptoms. *Journal of Child Psychology & Psychiatry, 56*(10), 1092-1100. <https://doi.org/10.1111/jcpp.12395>

Wiggins, J. L., Ureña Rosario, A., MacNeill, L. A., Krogh‐Jespersen, S., Briggs‐Gowan, M., Smith, J. D., & Wakschlag, L. S. (2023). Prevalence, stability, and predictive utility of the Multidimensional Assessment of Preschoolers Scales clinically optimized irritability score: Pragmatic early assessment of mental disorder risk. *International Journal of Methods in Psychiatric Research*, *32*(S1), e1991–e1991. <https://doi.org/10.1002/mpr.1991>

Wilson, M. K., Cornacchio, D., Brotman, M. A., & Comer, J. S. (2022). Measuring irritability in early childhood: A psychometric evaluation of the affective reactivity index in a clinical sample of 3- to 8-year-old children. *Assessment*, *29*(7), 1473-1481. <https://doi.org/10.1177/10731911211020078>

Zendarski, N., Galligan, R., Coghill, D., Payne, J. M., De Luca, C. R., & Mulraney, M. (2023). The associations between child irritability, parental distress, parental irritability and family functioning in children accessing mental health services. *Journal of Child and Family Studies*, *32*(1), 288–300. <https://doi.org/10.1007/s10826-022-02390-2>

Zhou, Q., Lengua, L. J., & Wang, Y. (2009). The relations of temperament reactivity and effortful control to children's adjustment problems in China and the United States. *Developmental Psychology*, *45*(3), 724-739. <https://doi.org/10.1037/a0013776>

Zik, J., Deveney, C., Ellingson, J., Haller, S. P., Kircanski, K., Cardinale, E., Brotman, M. A., & Stoddard, J. (2022). Irritability interrelations with anger and aggression, and the effects of the informant. *Journal of the American Academy of Child and Adolescent Psychiatry*, *59*(10), S196-S196. <https://doi.org/10.1016/j.jaac.2020.08.221>
